# Integrated analysis of metabolome and transcriptome reveals the mechanism of divergent acute heat stress responses in mango cultivars

**DOI:** 10.3389/fpls.2026.1805686

**Published:** 2026-06-12

**Authors:** Wenju Luo, Xianbin Hou, Lanjie Wei, Shiheng Liu, Ruixia Li, Zheng Teng, Cuifeng Yang, Yufeng Li, Zhengjie Zhu

**Affiliations:** 1 Guangxi Key Laboratory of Biology for Mango, College of Agriculture and Food Engineering, Baise University, Baise, China; 2 University Engineering Research Center for Preservation and Comprehensive Utilization of Subtropical Characteristic Agricultural Products in Guangxi, College of Agriculture and Food Engineering, Baise University, Baise, China; 3 Guangxi Old Revolutionary Area Revitalization Development Research Base, Baise, China

**Keywords:** 5’-AMP-activated protein kinase (AMPK), copper chaperone, cytochrome c, heat stress, *Mangifera indica* L.

## Abstract

**Background:**

Mango (*Mangifera indica* L.) growth is affected by temperature, and heat stress often occurs in southern China, leading to a sharp decline in mango quality and yield. Understanding the response mechanism of mangoes and conducting research on their acute heat stress response and genetic breeding are urgently needed.

**Results and discussion:**

Heat stress induces multiple physiological impairments in mango seedlings through oxidative stress. Metabolome analysis identified 12 major metabolite classes, with amino acids and derivatives (AADs), phenolic acids (PAs), flavonoids (FLs), and lignans and coumarins (Lc) being the most responsive to acute heat stress, which provides a solid basis for subsequent research. RNA-seq analysis revealed that 12,635 differentially expressed genes (DEGs) were related to photosynthesis, homeostasis regulation, stomatal regulation, and other key plant hormone pathways. Weighted gene co-expression network analysis identified that copper chaperone, cytochrome c, and 5’-AMP-activated protein kinase (AMPK) were the key genes involved in acute heat stress response. Integrated Pearson correlation analysis across 36 samples revealed a strong, statistically significant gene–metabolite network (|r| > 0.7, p < 0.001), with AMPK showing the most extensive associations to lipids, flavonoids, and amino acid derivatives. These findings provide a unique theoretical basis for studying early heat-stress response mechanisms and for genetic breeding of mango, and offer valuable information for breeding heat−tolerant mango varieties.

## 1 Introduction

Mango (*Mangifera indica* L.), often referred to as the “king of tropical fruits,” plays a significant economic and agricultural role in tropical regions (Hon et al., 2025). India leads global production, accounting for 41.5% of the world’s mango output (Akshatha et al., 2024). China ranks third globally in mango production, with a long cultivation history and over 400 introduced varieties, primarily grown in southern provinces such as Hainan, Guangdong, and Guangxi (Kong et al., 2024). The optimal growth temperature range is 24 °C to 26 °C. However, based on the ground meteorological observation from Baise City (the major mango production area in Guangxi), extreme heat events occur frequently, with daily maximum temperatures often exceeding 40 °C during critical phenological stages; a record high temperature of 42.1 °C was recorded on May 7, 2020, with nearby stations recording values as high as 47 °C during the 2024 heatwave (Kushwaha et al., 2025). Climate risk zoning studies define the threshold for mild heat injury as daily maximum temperature ≥ 35 °C. In a systematic survey of 41 orchards over 25,774 apical shoots, heat stress was statistically associated with reductions in the fruit-bearing shoot percentage (68.01%; p < 0.05) and the normal fruit set rate (30.76%). In parallel, *in vitro* assays on four mango cultivars under controlled temperature conditions (14 °C to 36 °C) confirmed that high temperature significantly reduces pollen germination rate (p < 0.001) and pollen tube growth, with cardinal maximum temperatures ranging from 30.4 °C to 34.3 °C depending on genotype (Liu et al., 2023). These physiological disruptions caused by heat stress ultimately undermine flowering and fruit retention, leading to yield losses of up to 50% in severely affected production areas (Sukhvibul et al., 2000).

Under heat stress, reproductive processes are notably impaired. Hebbar et al. (2018) observed reduced pollen germination and pollen tube growth in peanut and coconut genotypes under heat stress. Similarly, Liu et al. (2023) reported that heat stress negatively affected mango pollen germination rate, as well as superoxide dismutase and catalase activities and malondialdehyde content. Typically, mango exhibits embryo abortion, asynchrony between pollen development and stigma receptivity, and inhibited pollen germination and pollen tube growth under heat stress (Sorkheh et al., 2018). These physiological disruptions compromise fertilization, flowering, and fruit retention, ultimately reducing fruit quality and yield (Khanum et al., 2020).

At the physiological and biochemical levels, heat stress disrupts cellular metabolism, reduces membrane stability, slows respiration, impairs stomatal movement, lowers relative water content, and alters photosynthetic metabolite accumulation (Abd El-Samad et al., 2022; Yazied et al., 2024). In mango leaves, heat stress affects photosynthetic pigments, water status, nutrient homeostasis (Shaaban et al., 2022), leading to excessive accumulation of antioxidant enzymes and other antioxidants (Zhao et al., 2020). The resulting oxidative stress damages photosystem II (Yu et al., 2020), disrupts cell and mitochondrial membranes, and ultimately curtails plant growth (Richter et al., 2010; Alghamdi et al., 2023). Samad and Shaaban (2024) further demonstrated that without treatments such as fulvic acid or salicylic acid, mango trees under heat stress show reduced water status, photosynthetic performance, and biochemical activity, ultimately impairing growth, yield, and fruit quality.

At the molecular level, extreme temperatures interfere with heat-sensing and signal transduction pathways involving calcium, nitric oxide (NO), reactive oxygen species (ROS), and various kinases (He et al., 2023). Heat shock proteins (HSPs) and transcription factor families such as MYB, bHLH, and NAC are also adversely affected (Zhou et al., 2022; Li et al., 2021; Ashraf et al., 2012), collectively undermining mango productivity and quality.

Heat shock proteins are particularly important in the regulatory response to heat stress. The classic heat stress response involves heat shock transcription factors (HSFs) and HSPs, which recognize conserved heat shock elements (HSEs) in promoter regions to activate heat-responsive genes. Eukaryotic adaptation to hyperthermia entails complex regulatory networks formed by HSFs and multiple signaling pathways, enabling plants to adjust gene expression and acclimatize to elevated temperatures (Driedonks et al., 2015). Therefore, elucidating the mechanisms of the acute heat stress response and the molecular responses in mango leaves is essential for breeding heat-tolerant varieties and sustaining high yield and quality under heat stress. These are key steps toward ensuring crop productivity and resilience.

Although heat signaling and tolerance mechanisms have been well characterized in some plant species, studies on the molecular responses of mango leaves under heat stress are still limited. In this study, ‘Tainong No.1’ (TN), ‘Jinhuang’ (JH), and ‘Guiqi’ (GQ) belong to the same species *Mangifera indica* L., but exhibit divergent acute heat stress responses, with JH being the most tolerant, followed by TN, and GQ the most sensitive. We investigated the physiological, biochemical, and molecular mechanisms of three mango cultivars exposed to different durations of heat stress. The three cultivars were selected because they represent major commercial varieties in Guangxi with divergent acute heat stress responses: ‘Jinhuang’ is heat-tolerant, ‘Guiqi’ is sensitive, and ‘Tainong No.1’ is intermediate (based on field observations). This intraspecific divergence provides a powerful comparative system to identify key determinants of acute heat stress response without interspecific variation. To our knowledge, this study is the first to combine metabolomic and transcriptomic approaches across heat-contrasting mango cultivars. Seedlings were subjected to 40 °C for 2 h (W2), 4 h (W4), and 8 h (W8) to examine the associated physiological, biochemical, metabolomic, and transcriptomic changes. Using metabolomic and transcriptomic approaches, we analyzed the responses of the three cultivars to heat stress. Weighted gene co-expression network analysis (WGCNA) was employed to identify key genes involved in the acute heat stress response. This work aims to uncover potential mechanisms underlying plant adaptation to heat stress and to provide a theoretical foundation for understanding the acute heat stress response and supporting genetic breeding efforts. The findings may advance our understanding of mango heat resistance and offer novel insights for developing adaptable, sustainable mango varieties.

## 2 Materials and methods

### 2.1 Plant materials and heat stress treatment

Three mango (*Mangifera indica* L.) cultivars, ‘Tainong No.1’ (TN, accession No. ASX671FT), ‘Jinhuang’ (JH, ASX681FT), and ‘Guiqi’ (GQ, ASX911FT), were obtained from the Baise Mango Germplasm Resources Base (Baise, China). Their acute heat stress response order (JH > TN > GQ) was preliminarily determined based on field observations (Hou et al., 2024). Two−year−old, healthy, uniform seedlings were used.

The potting medium was a mixture of local garden soil and river sand (4:1, v/v). Seedlings were grown in plastic pots (50 cm diameter × 50 cm height; Huaxing Plastic Co., Baise, China) and maintained in a controlled−environment growth chamber (RXZ−500D, Ningbo Jiangnan Instrument Factory, Ningbo, China).

For heat stress treatment, the chamber was set to 40 °C (± 1 °C) with a relative humidity of 40-50% and a light intensity of 86.4-108.0 μmol·m^−2^·s^−1^ provided by LED lamps (Model T5, Philips, Amsterdam, The Netherlands). The durations of heat exposure were 2 h (W2), 4 h (W4), and 8 h (W8), following previously described heat stress protocols in mango and other fruit crops (**Table 1**; Liu et al., 2023). Plants were irrigated with tap water every two hours to maintain consistent soil moisture. The control group (CK) was maintained at 26 °C under otherwise identical conditions. A total of 10 plants per cultivar were used for each treatment. For each treatment (CK, W2, W4, W8) and each cultivar, three biological replicates were collected. Each biological replicate consisted of leaves taken from a single randomly selected plant. From each plant, the top five fully expanded leaves were sampled, and a 5 cm segment from the leaf tip was excised. The three samples (one per plant) were immediately frozen in liquid nitrogen and stored at −80 °C until further analysis. No tissue pooling was used; each biological replicate was processed individually.

**Table 1:** Layout of heat stress treatment experiment.

Cultivar	Heat stress treatment time			
	CK (26 °C) (C)	40 °C+2 h (W2)	40 °C+4 h (W4)	40 °C+8 h (W8)
Tainong No.1 (TN)	TC	TW2	TW4	TW8
Jinhuang (JH)	JC	JW2	JW4	JW8
Guiqi (GQ)	GC	GW2	GW4	GW8

### 2.2 Determination of physiological indices

The activities of catalase (CAT) and superoxide dismutase (SOD) were determined according to Zhang et al. (2017). Peroxidase (POD) activity was determined according to the method of Almeida et al. (2019). Malondialdehyde (MDA) content in mango leaves was determined by the trichloroacetic acid (TCA) method (Mohamed et al., 2020). Proline (Pro) content was determined following the method of Nguyen and Van (2020). The chlorophyll content and chlorophyll fluorescence parameters were determined according to the method of Comfort et al. (2025). All physiological measurements were performed using three biological replicates per treatment (each biological replicate derived from an independent plant). For each biological replicate, three technical replicates were measured, and the mean value was used for statistical analysis.

### 2.3 Metabolome analysis of mango leaves in response to heat stress

#### 2.3.1 Dry sample extraction

For metabolomic analysis, three biological replicates (independent plants) were analyzed per cultivar per treatment. Biological samples were placed in a freeze dryer (Scientz-100F) for vacuum freeze−drying. The samples were then ground to a powder using a grinder (MM 400, Retsch) at 30 Hz for 1.5 min. A 50 mg aliquot of sample powder was weighed using an electronic balance (MS105DM). Then, 1200 μL of precooled 70% methanol containing an internal standard (1200 μL per 50 mg sample) was added. Vortexing was performed once every 30 min for 30 s, and this was repeated six times. After centrifugation (12,000 rpm, 3 min), the supernatant was aspirated, and the sample was filtered through a microporous membrane (0.22 μm pore size) and stored in the injection vial for UPLC-MS/MS analysis (Chen et al., 2013).

#### 2.3.2 Chromatography and mass spectrometry conditions

The data acquisition system consisted of an ultra-performance liquid chromatography (UPLC) system (ExionLC™ AD, https://sciex.com.cn/) (Peng et al., 2022) and a tandem mass spectrometry (MS/MS) system (Applied Biosystems 6500 QTRAP, https://sciex.com.cn/) (Kong et al., 2024). The liquid chromatography conditions were as follows: Chromatographic column: Agilent SB-C18 (1.8 µm, 2.1 mm × 100 mm) (Agilent Technologies, Santa Clara, CA, USA); Mobile phase A consisted of ultrapure water containing 0.1% formic acid, and mobile phase B consisted of acetonitrile containing 0.1% formic acid. Both solvents were of LC−MS grade and purchased from Merck KGaA (Darmstadt, Germany). Elution gradient: The proportion of phase B was 5% at 0.00 min. The proportion of phase B increased linearly to 95% within 9.00 min and was maintained at 95% for 1 min, from 10.00 to 11.10 min. The proportion of phase B decreased to 5% and was maintained at 5% for 14 min. The flow rate was 0.35 mL/min, the column temperature was 40 °C, and the injection volume was 2 μL.

The mass spectrometry conditions were as follows: The electrospray ionization (ESI) temperature was 500 °C, the ion spray voltage (IS) was 5500 V (positive ion mode) and -4500 V (negative ion mode). The ion source gas I (GSI), gas II (GSII) and curtain gas (CUR) were set to 50, 60 and 25 psi respectively, and the collision-induced ionization parameter was set to high. QQQ scans were performed using multiple reaction monitoring (MRM) mode, and the collision gas (nitrogen) was set to medium. Through further optimization of declustering potential (DP) and collision energy (CE), DP and CE of each MRM ion pair were optimized (Chen et al., 2013; Kong et al., 2024). Based on the metabolites eluted in each period, a specific set of MRM ion pairs was monitored in each period.

#### 2.3.3 Principal component analysis, hierarchical cluster analysis, and Pearson correlation coefficient

PCA, HCA, and PCC were performed using R software (version 4.1.2; R Core Team, 2021). PCA was conducted using the prcomp function from the stats package. HCA was performed using the hclust function (Ward’s method) from the stats package, and the heatmap was generated using the ComplexHeatmap package (version 2.10.0; Gu et al., 2016). PCC was calculated using the cor function from the stats package, and the correlation plot was visualized using the corrplot package (version 0.92; Wei and Simko, 2021).

#### 2.3.4 Selection of differential metabolites and KEGG enrichment analysis

Differential metabolites were screened using OPLS-DA model (ropls package). Metabolites with VIP > 1 and |log_2_FC| ≥ 1 were considered differential (Chong and Xia, 2018). Kyoto Encyclopedia of Genes and Genomes (KEGG) pathway enrichment was performed using clusterProfiler (Yu et al., 2012) with hypergeometric test (p < 0.05). Key pathways were identified by combining enrichment and topology analysis.

### 2.4 Transcriptome analysis of mango leaves in response to heat stress

#### 2.4.1 RNA extraction and transcriptome sequencing

Total RNA was extracted from mango leaves using the RNeasy Plant Mini Kit (Qiagen, Hilden, Germany). RNA concentration and purity were assessed using a NanoDrop 2000 spectrophotometer (Thermo Fisher Scientific, Waltham, MA, USA), and RNA integrity was verified by agarose gel electrophoresis. For RNA−seq, three biological replicates (independent plants) were sequenced per cultivar per treatment. Each biological replicate was processed through library preparation and sequencing independently. No pooling of biological replicates was performed. Oligo(dT) magnetic beads were used to enrich the mRNA with poly (A) structure in total RNA (Illumina, San Diego, CA, USA), and the RNA was interrupted to a fragment of about 300 bp in length by an ion disruption buffer (Thermo Fisher Scientific, Waltham, MA, USA). RNA was used as a template to synthesize the first strand cDNA with hexamer random primer and reverse transcriptase (SuperScript™ IV, Invitrogen, Carlsbad, CA, USA), and the second strand cDNA was synthesized with the first strand cDNA as template. Then the double-stranded cDNA was purified to obtain a cDNA library. 3 μL USER enzyme (New England Biolabs, Ipswich, MA, USA) was used with size-selected, adaptor-ligated cDNA at 37 °C for 15 min followed by 5 min at 95 °C before polymerase chain reaction (PCR). Further PCR amplification was used to enrich the library fragments, and then the library was selected according to the fragment size. The total concentration and effective concentration of the library were detected by using Agilent 2100 Biological analyzer (Agilent Technologies, Santa Clara, CA, USA). Finally, paired−end sequencing (150 bp) was performed on an Illumina NovaSeq 6000 platform (Illumina, San Diego, CA, USA) using the TruSeq PE Cluster Kit v3−cBot−HS (Illumina). The sequencing protocol followed the manufacturer’s standard pipeline. Raw sequencing data were deposited in the NCBI Sequence Read Archive (SRA) under BioProject PRJNA1373361.

#### 2.4.2 Gene expression and function analysis

##### 2.4.2.1 Quantitative expression and differential expression analysis

The sequencing data were filtered using fastp (version 0.20.0), and sequencing error rate and GC content distribution were checked (Chen et al., 2018). The cleaned data were compared with the reference genome using HISAT2 (version 2.2.1) (Wang et al., 2020). Gene expression levels were quantified as Fragments Per Kilobase of transcript per Million mapped reads (FPKM) following the method described by Trapnell et al. (2010) using StringTie (version 2.1.4) (Pertea et al., 2015). DESeq2 (version 1.34.0) was used for differential gene expression analysis. Genes with log_2_ fold change ≥ 1 and false discovery rate (FDR) < 0.05 were considered differentially expressed genes (DEGs) (Love et al., 2014).

##### 2.4.2.2 Functional enrichment analysis of differentially expressed genes

Gene Ontology (GO) enrichment analysis was performed using GOATOOLS (version 1.0.0; Klopfenstein et al., 2018) with a significance threshold of FDR < 0.05. KEGG pathway enrichment analysis was performed using the R package clusterProfiler (version 4.2.2; Yu et al., 2012) with a p−value threshold of < 0.05.

##### 2.4.2.3 Weighted gene co-expression network analysis

Weighted gene co−expression network analysis (WGCNA) was performed using the WGCNA package (version 1.72−1) in R (version 4.1.2, Langfelder and Horvath, 2008). To reduce background noise, only genes with FPKM ≥ 1 in at least 50% of the 36 samples were retained. The expression data were then normalized using the *varianceStabilizingTransformation* function in DESeq2 prior to network construction.

Soft−thresholding power selection: To construct a scale−free network, the pickSoftThreshold function was used to evaluate a range of soft−thresholding powers (β) from 1 to 20 with an increment of 1. For each power, the scale−free topology fit index (R²) was calculated. The optimal soft−thresholding power was selected as the lowest β at which the scale−free topology R² reached at least 0.85 and the mean connectivity remained relatively stable. In our dataset, β = 6 (signed network) achieved an R² of 0.87 and was therefore chosen for subsequent network construction.

Network construction and module detection: Using the selected soft−thresholding power (β = 6), an adjacency matrix was computed based on the biweight mid−correlation (bicor) to enhance robustness against outliers. The adjacency matrix was then transformed into a topological overlap matrix (TOM), and the corresponding dissimilarity matrix (1−TOM) was calculated. Genes were clustered using average linkage hierarchical clustering, and modules were identified using the dynamic tree−cut algorithm (cutreeDynamic) with the following parameters: minModuleSize = 30, deepSplit = 2, and cutHeight = 0.995. Modules whose eigengenes were highly correlated (Pearson correlation > 0.75) were merged using the mergeCloseModules function with mergeCutHeight = 0.25.

Hub gene identification: For each module, module membership (MM, also known as kME) was calculated as the Pearson correlation between the expression profile of each gene and the module eigengene. Genes with |MM| > 0.8 were considered candidate hub genes within their respective modules. Additionally, gene significance (GS) was computed as the correlation between gene expression and heat stress treatment duration (coded as 0 for CK, 1 for 2 h, 2 for 4 h, and 3 for 8 h). Hub genes with high MM and high |GS| were prioritized for further analysis. The network visualization was performed using Cytoscape (version 3.10.4).

#### 2.4.3 Integrated correlation analysis of metabolome and transcriptome

To identify statistically significant associations between differential metabolites (DAMs) and differential expressed genes (DEGs), we performed an integrated correlation analysis. First, we selected DAMs and DEGs that were significantly altered (|log_2_FC| ≥ 1, VIP > 1 for metabolites; |log_2_FC| ≥ 1, FDR < 0.05 for genes) in at least one treatment (W2, W4, or W8) compared to CK across the three cultivars.

Pearson correlation coefficients were calculated between the three hub genes (LOC123192271, LOC123192299, LOC123192703) and all differential metabolites using the 36 samples. Correlation pairs with |r| > 0.7 and p < 0.01 were considered significant. The correlation network was visualized using Cytoscape (version 3.10.4). Edge width was scaled to |r|, and edge color indicated positive (red) or negative (blue) correlation. The resulting metabolite−gene correlation pairs were visualized as a network using Cytoscape (version 3.10.4) (Shannon et al., 2003). Key metabolites and genes with high degree centrality (number of connections) were further analyzed for functional enrichment using clusterProfiler (Yu et al., 2012). This integrated workflow allowed us to identify hub metabolites and hub genes that potentially co−regulate or functionally interact during the heat stress response. The full correlation results are presented in **Supplementary Table 3**.

### 2.5 Data analysis

SPSS (version 25.0, IBM Corp., Armonk, NY, USA) was used for analysis of variance (ANOVA) of physiological data, and Duncan’s method was used for multiple comparisons. All statistical analyses were based on three biological replicates per treatment. Data are presented as mean ± standard deviation (SD) unless otherwise stated. Metabolome and transcriptome data were processed using Excel (version 2016, Microsoft Corp., Redmond, WA, USA), and images were generated using GraphPad Prism (version 10.1.2, GraphPad Software, San Diego, CA, USA) and PowerPoint (version 2016, Microsoft Corp., Redmond, WA, USA).

## 3 Results

### 3.1 Physiological effects of heat stress on leaves of three different mango varieties

**Figure 1:**
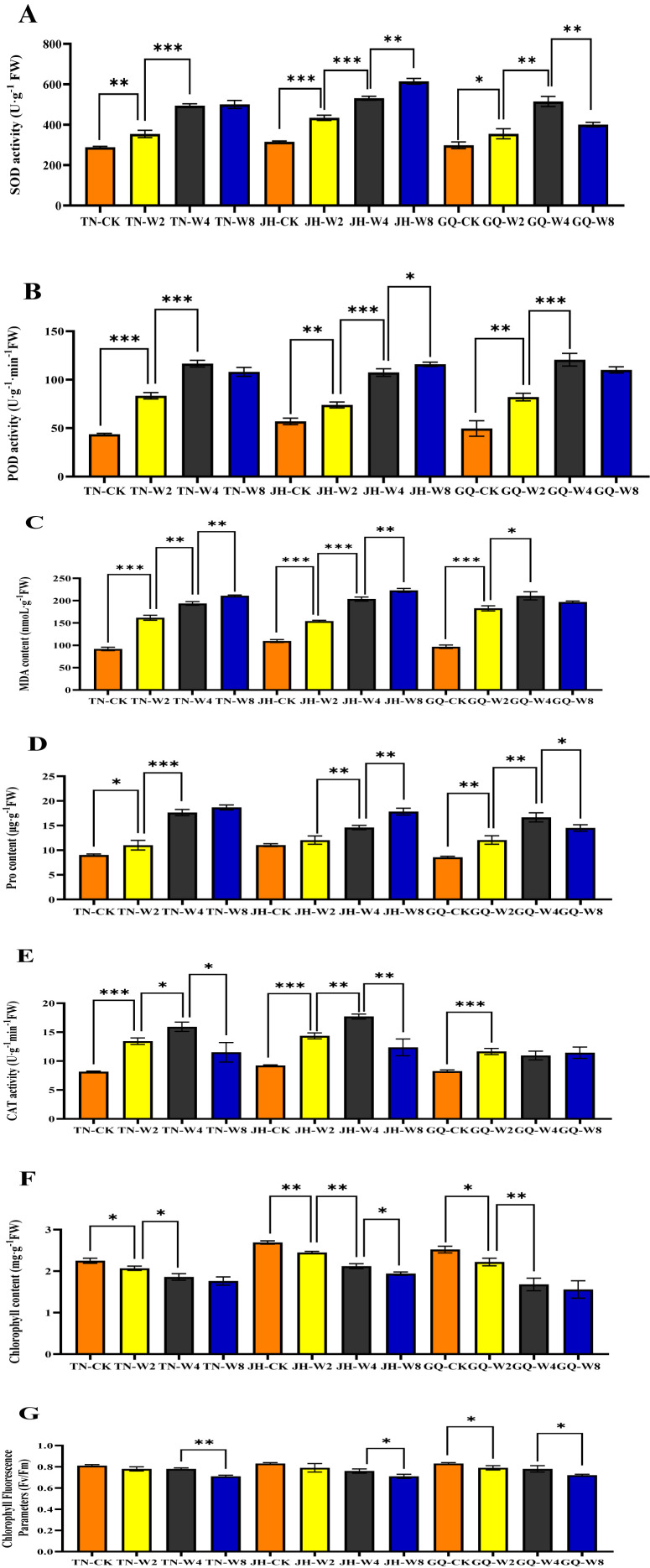
Physiological responses of three mango cultivars under acute heat stress (40 °C, 2-8 h). **(A)** Superoxide dismutase (SOD) activity, **(B)** peroxidase (POD) activity, **(C)** malondialdehyde (MDA) content, **(D)** proline (Pro) content, **(E)** catalase (CAT) activity, **(F)** chlorophyll content, and **(G)** chlorophyll fluorescence parameters (Fv/Fm). Data are presented as mean ± SD (n = 3 biological replicates, each with 3 technical replicates). Different letters above bars indicate significant differences (p < 0.05) based on one−way ANOVA followed by Duncan’s multiple range test. *, **, *** indicate p < 0.05, p < 0.01, and p < 0.001, respectively, compared to the control (CK) within each cultivar. ns, not significant.

SOD activity in all varieties significantly increased from CK to W4 and W8, except for a slight decrease in GQ at W8, suggesting that oxidative stress was induced and plants responded by enhancing SOD activity to scavenge superoxide radicals. POD activity generally increased with increasing stress intensity, though a slight decline was observed in some varieties at W8, possibly due to regulatory adjustments of enzyme activity. The MDA content in the leaves of the three mango varieties increased markedly, indicating increased membrane lipid peroxidation and consequent cell membrane damage. Similarly, Pro content rose significantly across varieties, reflecting osmotic adjustment through proline accumulation to maintain cellular water balance. CAT activity increased under low to moderate stress (W2, W4) but declined under high stress (W8), suggesting potential enzyme system overload or inhibition. Chlorophyll content generally decreased, consistent with stress-induced degradation of photosynthetic pigments and reduced photosynthetic capacity. The decline in chlorophyll fluorescence parameters suggests a decrease in the photochemical efficiency of PSII, likely due to light-induced inhibition or damage (**Figure 1**).

### 3.2 Metabolome analysis of mango leaves in response to heat stress

**Figure 2:**
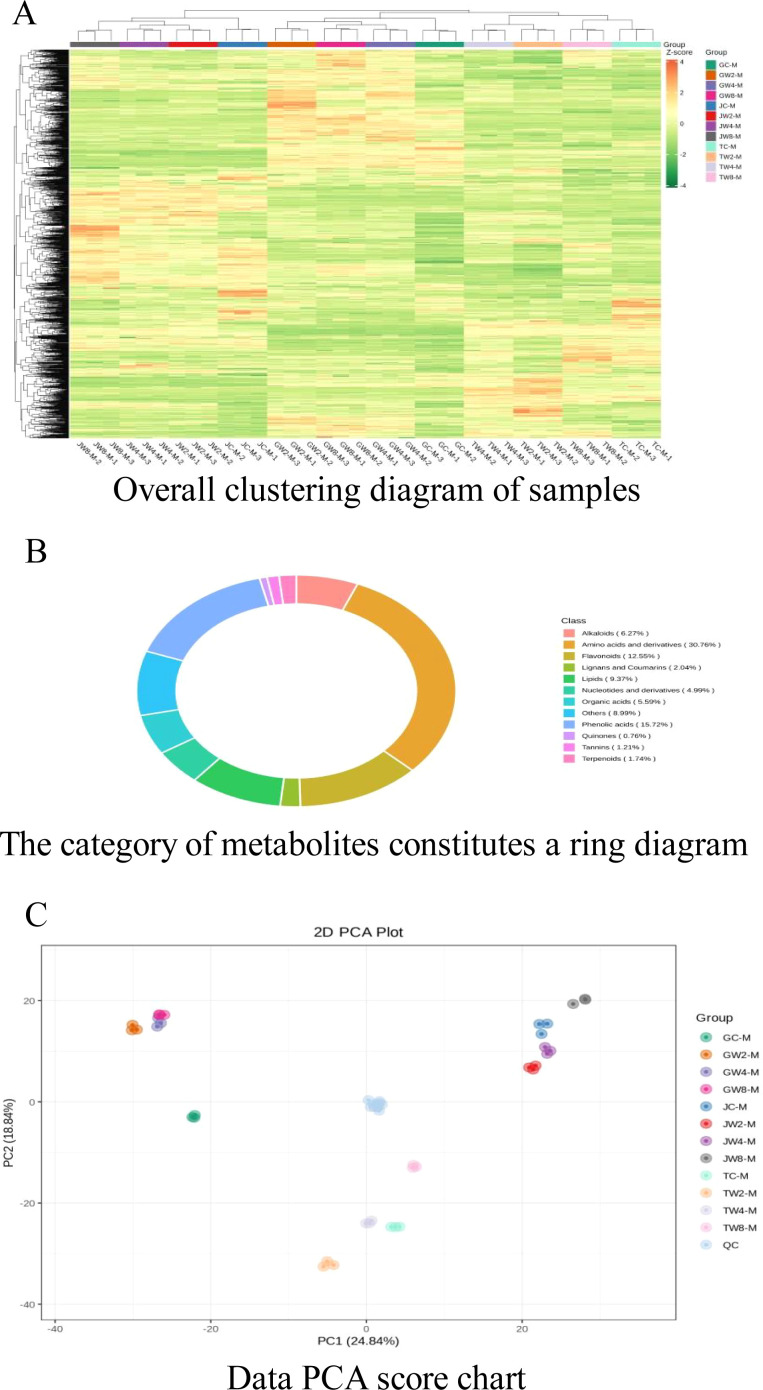
Metabolomic profiling of mango leaves under heat stress. **(A)** Hierarchical clustering heatmap of all 1323 identified metabolites across 36 samples (3 cultivars × 4 treatments × 3 replicates). Colors represent normalized peak intensities. **(B)** Pie chart showing the distribution of metabolite classes, with the percentage of each class indicated. AADs, amino acids and derivatives; PAs, phenolic acids; FLs, flavonoids; Lc, lignans and coumarins; others (including organic acids, tannins, terpenoids, etc.) as defined in the main text. **(C)** Principal component analysis (PCA) score plot of all samples. PC1 and PC2 explain 24.84% and 18.84% of the total variance, respectively. Each point represents one biological replicate, with colors indicating cultivars (TN, JH, GQ) and shapes indicating treatment times (CK, W2, W4, W8). Ellipses represent 95% confidence intervals for each treatment group.

Using UPLC-MS/MS analysis of 36 mango leaf samples, 1,323 metabolites were identified. The annotated metabolites belonged to one of 12 metabolite classes (**Figure 2A**). The highest percentage of compounds was amino acids and derivatives (AADs, 30.76%), followed by phenolic acids (PAs, 15.72%) and flavonoids (FLs, 12.55%) (**Figure 2B**). PCA revealed that biological replicates within each treatment group clustered together (**Figure 2C**), indicating good consistency among replicates. PC1 and PC2 explained 24.84% and 18.84% of the total variance, respectively. Pearson correlation coefficients (PCC) among all samples ranged from 0.52 to 1.0, with an average of 0.83 (**Supplementary Figure 1**). This moderate to high correlation further confirms the consistency of the experimental replicates.

**Figure 3:**
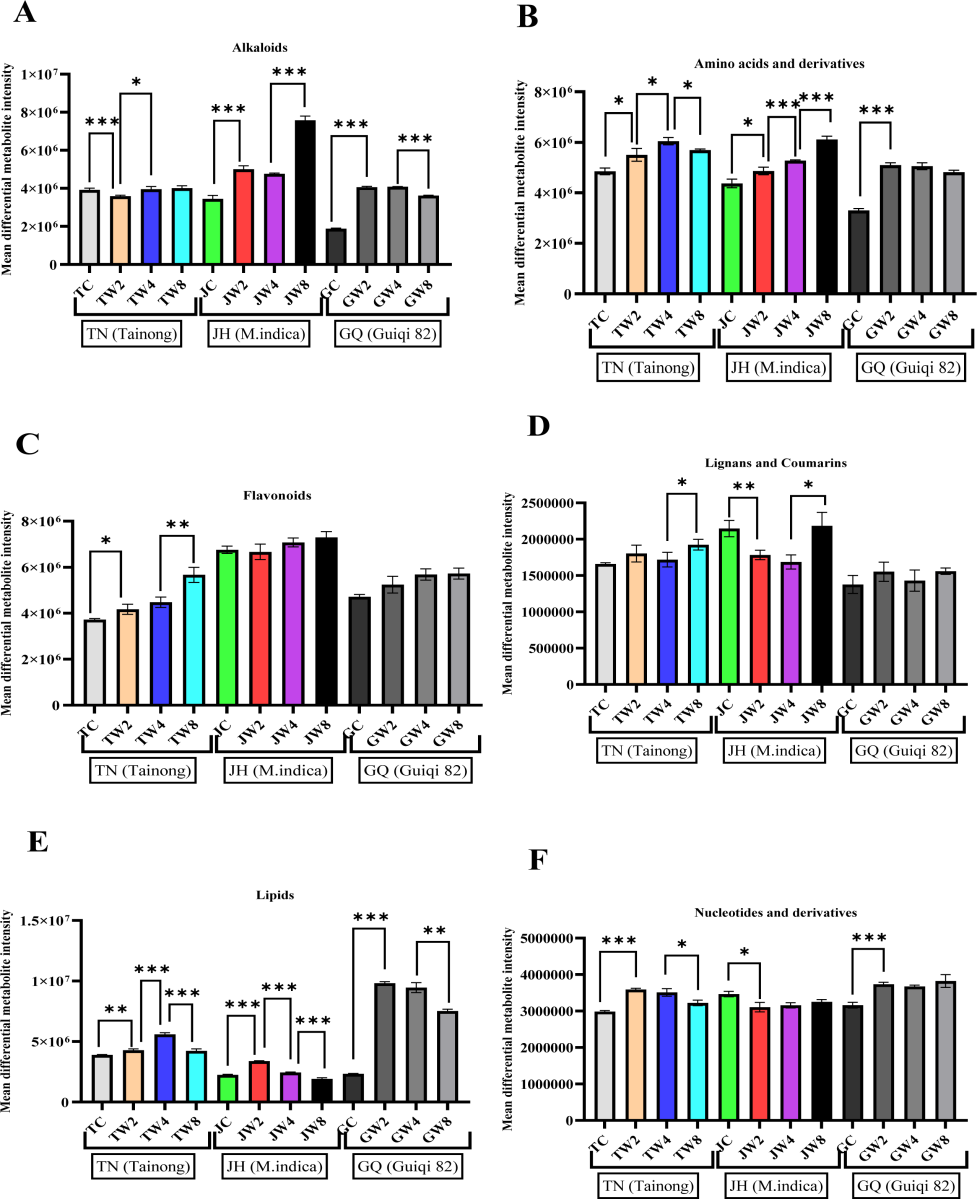
Mean differential metabolite intensity of 12 key metabolic pathways in three mango cultivars under acute heat stress. The six subfigures correspond to **(A)** Alkaloids, **(B)** Amino acids and derivatives, **(C)** Flavonoids, **(D)** Lignans and coumarins, **(E)** Lipids, and **(F)** Nucleotides and derivatives, respectively. The remaining six pathways are presented in **Supplementary Figure 2**. Three mango cultivars (TN, Tainong No.1; JH, Jinhuang; GQ, Guiqi 82) were subjected to heat stress at four time points (control, 2 h, 4 h, 8 h). Bars represent mean ± SD. Asterisks indicate significant differences compared with the control (*p < 0.05, **p < 0.01, ***p < 0.001).

The dynamic changes in mean differential metabolite intensity across 12 key metabolic pathways were determined in three mango cultivars under acute heat stress (**Figure 3**; **Supplementary Figure 2**). JH exhibited stable and significant upregulation in most defense-related pathways, including alkaloids, amino acids and derivatives, flavonoids, lignans and coumarins, organic acids, tannins, and terpenoids. Notably, alkaloids in JH increased continuously and reached more than twice the control level after 8 h of stress, showing a significant increase (p < 0.001). TN showed a delayed response, with most pathways significantly altered only at 4–8 h, while lipids exhibited a transient increase followed by a decline. GQ displayed weak induction in core defense pathways, with only organic acids, lipids, and nucleotides showing strong upregulation. Overall, JH possessed a more coordinated and sustained metabolic response across multiple pathways, which is closely associated with its superior heat tolerance.

### 3.3 Number of differential metabolites in response to heat stress in mango

We further studied the number of differential metabolites in the mango response to acute heat stress. As shown in **Table 2**, the number of upregulated and downregulated metabolites were as follows. In JH, the total number of differential metabolites increased with prolonged heat stress, from 185 (JW2) to 234 (JW8), whereas in ‘Tainong No.1’ (TN) and ‘Guiqi’ (GQ) the total numbers first increased then decreased (**Table 2**). We observed that the total number of metabolites of ALs (8), AADs (13), FLs (12), Lc (2), Nucleotides and derivatives (NDs) (5), OAs (10), Others (1), and PAs (10) in JH increased with increasing time. While lipids (11) decreased. The number of lipid (1) and OAs (2) metabolites of TN increased to varying degrees. The total number of metabolites decreased in ALs (7), AADs (49), FLs (3), Lc (10), NDs (7), Others (4), PAs (38), and Terpenoids (4). The number of metabolites in AADs (12), OAs (4), and ‘other’ (12) metabolic pathways increased gradually in GQ. With prolonged heat stress, the number of metabolites in ALs, AADs, FLs, Lc, Lipids, NDs, Others and PAs in JH was lower than that in TN and GQ at 4-8 h of heat stress. Finally, metabolomic comparison showed that ALs, AADs, FLs, Lc, Lipids, NDs, OAs, Others (including Alcohol compounds, Aldehydes, Chromones, Ketones, Saccharides, Stilbenes, and Vitamins), PAs, Quinones, Tannins and Terpenoids were the main metabolites classes involved in the heat stress response in mango leaves. Comparison across varieties using Venn diagrams revealed 51, 97, and 171 common DAMs in TN, JH, and GQ, respectively (**Supplementary Figure 3**).

**Table 2:** Number of differentially accumulated metabolites (DAMs) in mango in response to heat stress.

Class I	TW2	TW4	TW8	JW2	JW4	JW8	GW2	GW4	GW8
ALs	8/6	8/0	5/2	7/2	6/2	16/1	22/1	21/0	26/0
AADs	43/82	44/23	49/27	45/30	50/17	56/32	97/1	94/6	104/6
FLs	20/12	7/0	27/2	4/0	9/2	11/5	20/1	16/1	20/1
Lc	5/7	1/1	2/0	0/1	1/1	1/2	2/0	1/0	2/0
Lipids	9/26	9/18	3/33	18/24	8/14	2/29	63/3	45/4	44/5
NDs	5/9	6/8	2/5	4/6	5/4	8/7	11/3	6/3	6/2
OAs	6/9	8/5	6/11	5/2	4/0	10/7	10/1	8/4	11/4
Others	7/10	8/6	9/4	8/3	7/4	6/6	9/0	14/3	18/3
PAs	28/18	22/1	8/0	14/10	21/2	22/12	28/1	39/1	35/2
Quinones	1/0	0/0	0/0	1/0	0/0	0/0	0/0	0/0	0/0
Tannins	2/1	0/0	1/0	0/	0/0	1/0	3/0	2/0	3/0
Terpenoids	1/5	0/3	0/2	0/1	4/0	0/0	6/0	7/0	7/0
Total	320	178	198	185	161	234	282	275	299

Values are expressed as “number of upregulated metabolites/number of downregulated metabolites”. Up−regulation: log_2_FC ≥ 1, VIP > 1; down−regulation: log_2_FC ≤ -1, VIP > 1. “0/0” indicates no differential metabolites of that class at that time point. Total refers to the sum of all differential metabolites (both up− and downregulated) across all classes.

KEGG pathway enrichment analysis revealed DAMs in TN (TC vs. TW2/TW4/TW8). These were mainly concentrated in glucosinolate biosynthesis, and in the valine, leucine, and isoleucine biosynthesis metabolic pathways (**Supplementary Figure 4**). DAMs were enriched in JH (JC vs JW2/JW4/JW8) in important metabolic pathways, including valine, leucine, and isoleucine degradation, linoleic acid metabolism, zeatin biosynthesis, glucosinolate biosynthesis, and D-Amino acid metabolism (**Supplementary Figure 4**). However, in GQ (GC vs. GW2/GW4/GW8), DAMs were mainly enriched in glucosinolate biosynthesis, aminoacyl-tRNA biosynthesis, linoleic acid metabolism, 2-oxocarboxylic acid metabolism (**Supplementary Figure 4**). Interestingly, linoleic acid metabolism, glucosinolate biosynthesis, amino acid biosynthesis, and metabolic pathways were significantly different among the three varieties.

In the DAMs of TN (TC vs. TW2/TW4/TW8), we observed that the metabolite 1,6-di-o-galloyl-2-o-feruloyl-β-d-glucose accumulated progressively with increasing duration of heat stress exposure. In contrast, the most significantly down-accumulated DAMs were Phe-Thr-His and Allopurinol (**Supplementary Figure 5**). Among the DAMs in JH (JC vs. JW2/JW4/JW8), Chlorogenic acid, 2’, 4’, 6’-trihydroxydihydrochalcone, and 9-hypoxanthine showed marked upward accumulation with prolonged heat stress treatment. Meanwhile, Arg-Tyr-Leu-Lys, 9,12,13-trihydroxy-10, 15-octadecadienoic acid were notably down-accumulated (**Supplementary Figure 5**). For GQ (GC vs. GW2/GW4/GW8), interestingly, the most significantly up-accumulated DAM across time points was consistently 2,5-dimethylpyrazine. The most prominently down-accumulated DAMs included chrysoeriol-7-o-rutinoside, 3-hydroxy-4-methoxybenzoic acid, and p-coumaric acid (**Supplementary Figure 5**).

### 3.4 Transcriptome analysis of mango response to heat stress

After sequencing quality control, a total of 264.43 Gb of clean data were obtained, with each sample yielding at least 6 Gb of clean data. The number of raw reads ranged from 43,201,464 to 71,530,116, and the number of clean reads ranged from 42,072,442 to 70,207,462. The GC content ranged from 41.47% to 43.24%, and the percentage of reads with a Q30 score exceeded 89.86% for all 36 samples. These results indicate that the sequencing data are of high quality and suitable for subsequent analyses (**Supplementary Table 1**).

**Figure 4:**
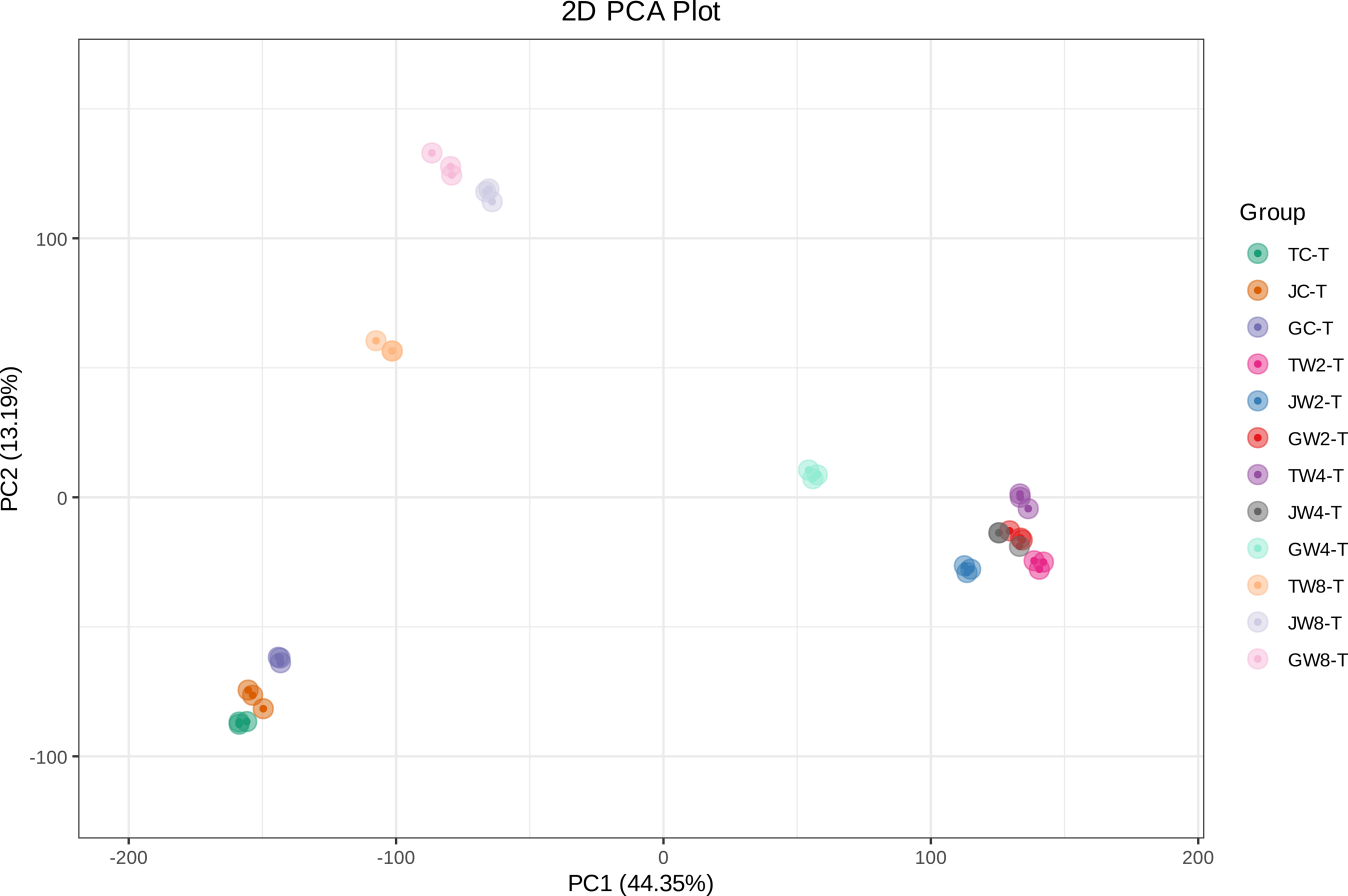
Principal component analysis (PCA) of transcriptome data. PCA was performed on FPKM values of all genes across 36 samples (3 cultivars × 4 treatments × 3 replicates). PC1 explains 44.35% of the variance, separating treatment groups (CK, W2, W4, W8); PC2 explains 13.19% of the variance, partially separating cultivars. Each symbol represents one biological replicate. The clear clustering of treatment groups indicates a stronger effect of heat stress duration than genotype on global gene expression.

FPKM distribution was lower in the W2 treatment, and the highest expression level was observed in the CK, indicating that the heat stress significantly affected gene expression (**Supplementary Figure 6**). The average Pearson correlation coefficient (PCC) among all samples was 0.64 (**Supplementary Figure 7**), indicating moderate correlation, which is acceptable for biological replicates given the expected variation among independent plants. PCA of the FPKM values revealed clear separation of treatment groups (CK, W2, W4, W8) along PC1 (44.35% of the variance), while the three cultivars partially separated along PC2 (13.19% variance), suggesting that heat stress treatment had a stronger effect on gene expression than genotype (**Figure 4**).

### 3.5 Differential gene expression in response to heat stress in mango

According to the Venn diagram, the total numbers of differentially expressed genes (DEGs) shared between the treatment and control groups were 3,078, 4,624, and 3,837 for TN, JH, and GQ, respectively (**Supplementary Figure 8**). During GO functional annotation, we observed that at each heat stress time point for all three cultivars, differentially expressed genes were significantly enriched in both metabolic processes and cellular processes within the Biological Process (BP) category. In the Cellular Component (CC), differentially expressed genes were significantly enriched in “protein-containing complex” and “cellular anatomical entity” terms. In Molecular Function (MF), the differentially expressed genes were significantly enriched in catalytic activity and binding terms. Over time under heat stress, the number of DEGs in JH and GQ gradually decreased. The number of DEGs in TN first increased and then decreased (**Supplementary Figure 9**).

KEGG enrichment analysis showed that in TN (TC vs. TW2/TW4/TW8), differentially expressed genes were significantly enriched in protein processing in the endoplasmic reticulum, photosynthetic antenna proteins, and metabolic pathways (**Supplementary Figure 10**). In JH (JC vs. JW2/JW4/JW8), the differentially expressed genes were significantly enriched in protein processing in the endoplasmic reticulum, circadian rhythm (plant), photosynthetic antenna proteins, metabolic pathways, and biosynthesis of secondary metabolites (**Supplementary Figure 10**). In GQ (GC vs. GW2/GW4/GW8), the differentially expressed genes were significantly enriched in protein processing in the endoplasmic reticulum, circadian rhythm (plant), plant hormone signal transduction, photosynthesis, metabolic pathways, and biosynthesis of secondary metabolites (**Supplementary Figure 10**).

### 3.6 Specific differential gene expression in three mango cultivars

Heat stress caused serious adverse effects on mango leaves. We conducted in-depth studies on photosynthesis, antenna proteins-related, homeostasis-related, stomatal-related, heat stress-related, MAPK and plant hormone-related pathways.

#### 3.6.1 Differentially expressed genes related to photosynthesis

**Figure 5:**
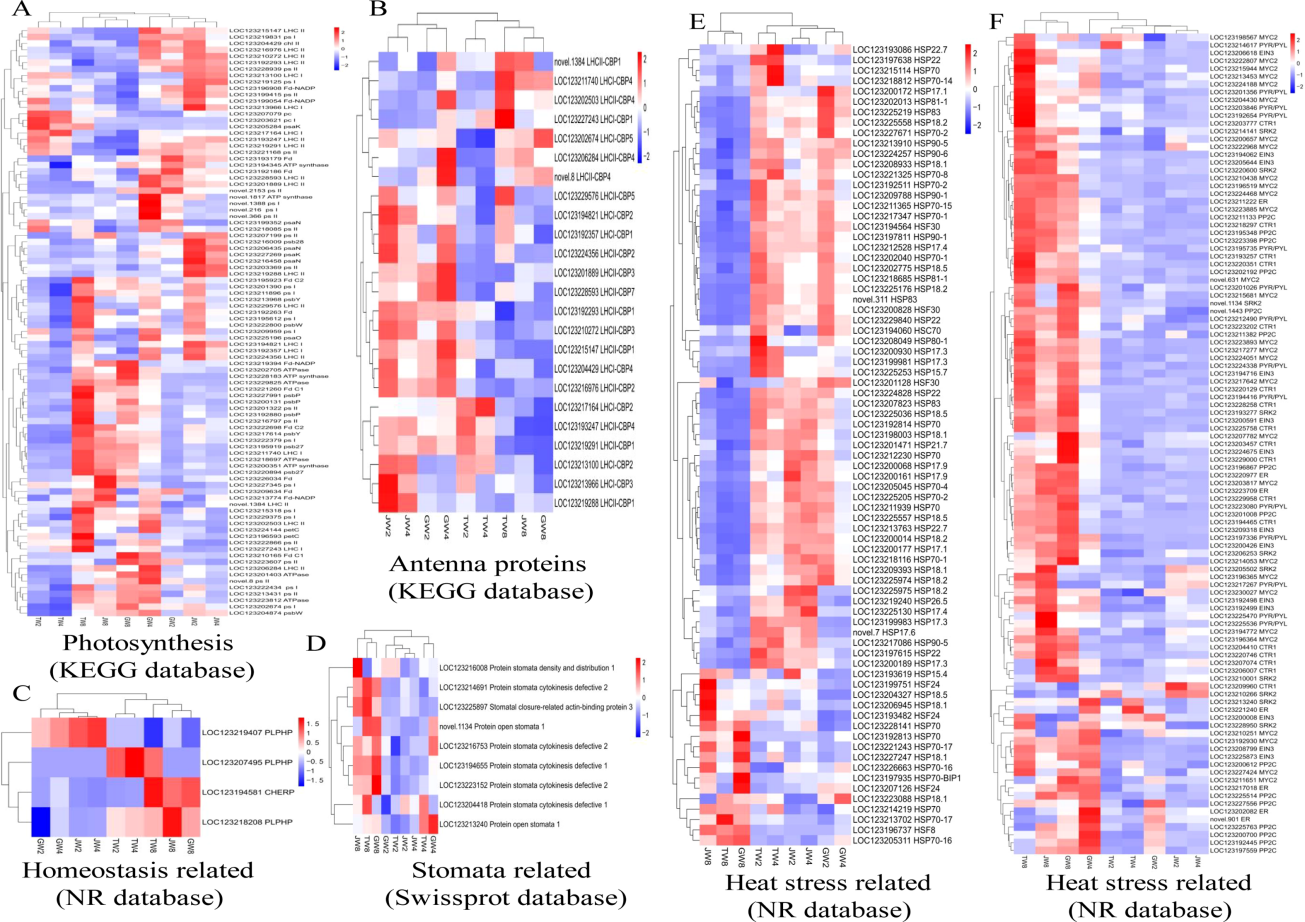
Expression patterns of differentially expressed genes involved in key pathways. **(A)** Photosynthesis−related genes (photosystem I/II, ferredoxin, ATP synthase, light−harvesting complexes). **(B)** Antenna proteins. **(C)** Homeostasis−related genes. **(D)** Stomatal regulation genes. **(E)** Heat shock protein (*HSP/HSF*) genes. **(F)** MAPK and plant hormone signaling pathway genes. Colors represent log_2_ fold change values (log_2_FC) compared to the control (CK) for each cultivar and treatment. Red indicates upregulation, blue indicates down−regulation. Only genes with |log_2_FC| ≥ 1 and FDR < 0.05 are shown. The full list of gene IDs is provided in **Supplementary Table 2**.

After removing unknown and duplicate genes, we identified a total of 87 DEGs. These included components of photosystem I (*psaK*, *psaN*, *psaO*), photosystem II (*psbY*, *psbW*, *psbP*, *psb27*, *psb28*), ferredoxin (*Fd*, *Fd C1*, *Fd C2*, *Fd-NADP*), the cytochrome complex iron−sulfur subunit (*petC*), ATP synthase/ATPase, light−harvesting complexes (*LHC I*, *LHC II*), and other related factors (**Figure 5A**; **Supplementary Table 2**). In the JH variety, the expression levels of *PSI* (LOC123222434) and *Fd* (LOC123226034) gradually increased under prolonged heat stress and were higher than those observed in TN and GQ. Other genes such as *PSII* (LOC123216009), *Fd-NADP* (LOC123219394), and *PSII* (novel.366) also showed differential expression patterns. The expression levels of *PSII* (LOC123216009) and *PSII* (novel.366) decreased gradually under stress but remained higher than in the other two varieties. This expression pattern may partially explain the enhanced photosynthetic potential observed in JH. Other genes, including *PSII* (LOC123200131), *ATPase* (LOC123201322), *PSII* (LOC123221168), *Fd* (LOC123222698), and *PSII* (LOC123227991), were downregulated under heat stress. Notably, with prolonged heat stress exposure, the expression of *PSII* (LOC123200131) and *ATPase* (LOC123201322) exhibited a gradual increase. These two genes were also downregulated in GQ, but not in TN.

The expression of *Fd* (LOC123222698) decreased gradually, and this gene was downregulated in TW2 and GW8; however, there was no differential expression at other time points. In addition to these changes, we also noticed that JH had the largest number of upregulated differential genes in the *PSI* and *PSII* systems, followed by GQ and TN. *LHCII* (LOC123210272), *LHCII* (LOC123192293), and *LHC I* (LOC123213100) were significantly upregulated in JH. It is speculated that after heat stress, JH upregulates light-harvesting complex II chlorophyll a/b binding proteins 2 and 3, maintaining internal balance and ensuring normal photosynthesis (**Figure 5A**).

Finally, we also analyzed 24 PSI− and PSII−related DEGs (**Supplementary Table 2**). These differentially expressed genes varied, and JW8 and TW8 had a greater number of genes that were not differentially expressed. When heat stress lasted too long, these genes were no longer differentially expressed, suggesting possible loss of function under prolonged stress. Interestingly, we found a special differential gene, *PSII* (photosystem II 13 kDa protein, LOC123216009), which was upregulated in each time period for all three varieties, and its expression gradually decreased over time under heat stress (**Supplementary Table 2**). It is speculated that this gene is involved in the process of electron transfer and energy conversion in photosynthesis, which is mainly responsible for the use of light energy to split water, release oxygen, and produce *ATP* and *NADPH*, and plays an important role in photosynthesis. 13 kDa protein is also involved in the maintenance of the stability of the PSII complex, but the specific regulatory mechanism requires further study.

#### 3.6.2 Differentially expressed genes related to antenna proteins

Next, we studied antenna proteins involved in photosynthesis (**Figure 5B**; **Supplementary Table 2**). We found that *LHCI-CBP1* (LOC123219288) and *LHCI-CBP3* (LOC123213966) were significantly upregulated in JW2, and their expression levels were higher than those of the other two varieties at the same time. Only a few genes showed significant expression at GW4 (*LHCI−CBP1*, *LHCI−CBP4*) and at TW8 (*LHCII−CBP5*, *LHCI−CBP1*, *LHCI−CBP4*); most genes were not significantly expressed. In **Supplementary Table 2**, it can be seen that regardless of cultivar, most of the differentially expressed genes were significantly upregulated at W2, with JH having the largest number of upregulated DEGs. Finally, we observed two differential genes, *LHCII* (LOC123202503) and *LHCII* (LOC123210272). They were not differentially expressed at W4, but in JH they were downregulated at the other time points. *LHCII* (LOC123210272) was upregulated at all time periods except TW8 and GW8, and its expression level showed a decreasing trend over time. The expression levels of these two differential genes in JH were higher than those in TN and GQ. It is speculated that *LHCII* can more effectively absorb and transmit light energy, thus supporting efficient photosynthesis. At the same time, it has a light protection mechanism and can help plants cope with strong light irradiation, reduce light damage, and improve heat stress resistance.

#### 3.6.3 Differentially expressed genes related to homeostasis

Homeostasis refers to the adaptive mechanism by which plants maintain a stable internal environment and respond to stress. We conducted an in-depth analysis of the genes related to the homeostasis pathway in mangoes (**Figure 5C**; **Supplementary Table 2**). We found four related differential genes in NR database (LOC123194581, LOC123207495, LOC123218208, LOC123219407). These four differential genes were not differentially expressed at W8 (except for the downregulation of LOC123219407 in JW8), which is speculated to result from the loss of gene regulation under excessive heat stress.

In addition, JW8 (*PLPHP*, LOC123218208), TW8 (*CHERP*, LOC123194581), and TW4 (*PLPHP*, LOC123207495) were significantly differentially expressed. We focused on the differential gene encoding pyridoxal phosphate homeostasis protein-like isoform X1 (*PLPHP*, LOC123207495), which was upregulated in JH and TN at W2 and W4, but showed no differential expression in GQ. *PLPHP* in JH and TN responded to heat stress with altered expression levels, suggesting that the acute heat stress response of JH and TN is stronger than that of GQ.

#### 3.6.4 Differentially expressed genes related to stomata regulation

Nine related differential genes were found in the SwissProt database (**Figure 5D**; **Supplementary Table 2**). These included JW8 (protein stomata density and distribution 1, LOC123216008), TW8 (protein stomata cytokinesis defective 1, LOC123204418), GW8 (protein stomata cytokinesis defective 2, LOC123223152), and GW4 (protein open stomata 1, LOC123213240). We focused on protein stomatal density and distribution 1 (*PSDD1*) (LOC123216008). It is noteworthy that over time under heat stress, the expression of this gene in JH gradually increased, but in TN and GQ, the expression gradually decreased. It is speculated that *PSDD1* in JH, as a stomatal regulatory gene, it is speculated that PSDD1 in JH, as a stomatal regulatory gene, is involved in gas exchange, water regulation, photosynthesis, respiration, transpiration, and heat stress response. The regulatory ability of these aspects may be greater than that of the other two varieties, and JH may have the greatest heat stress resistance.

#### 3.6.5 Differentially expressed genes related to heat stress

We identified 78 differentially expressed genes related to the heat shock protein synthesis pathway in the NR database (**Figure 5E**; **Supplementary Table 2**). Significant upregulation was observed in JW8 (*HSF24*, LOC123199751; *HSP18.5*, LOC123204327; *HSP18.1*, LOC123206945; *HSF24*, LOC123193482), TW8 (*HSP70*-17, LOC123213702), GW8 (*HSP70-BIP1*, LOC123197935; *HSF24*, LOC123207126), TW4 (*HSP22.7*, LOC123193086; *HSP22*, LOC123197638; *HSP70*, LOC123215114; *HSP70-14*, LOC123218812). Interestingly, most of the differentially expressed genes were upregulated under heat stress. We focused on the gene encoding *HSP30* (LOC123194564), which was upregulated in all three varieties at all time points, with JH having the highest expression. It is speculated that *HSP30* in JH plays the most significant and stable role in antioxidant defense, which can help plants resist oxidative damage and enhance their stress resistance.

#### 3.6.7 Differentially expressed genes related to signal transduction

We analyzed 511 MAPK-related genes, 119 genes related to MAPK, plant hormones, and plant pathogen interactions, and 117 genes related to MAPK and plant hormones. We focused on the MAPK and plant hormone signaling pathway-related differential genes. We found that most of the differentially expressed genes showed significant changes at W2. With an increase in temperature, the expression of these genes either decreased or showed no differential expression (**Figure 5F**; **Supplementary Table 2**). Over time under heat stress, the expression levels of MAPK and plant hormone-related differential genes in the leaves of the three mango varieties increased by the W8 time point. Take JH leaf as an example: serine/threonine-protein kinase *CTR1* (LOC123207074, LOC123206007), *transcription factor MYC2* (LOC123194772), *PYR/PYL* (LOC123225470, LOC123225536), *SRK2* (LOC123205502) were upregulated. In TN, ethylene-insensitive protein 3 (EIN3, LOC123206618), *MYC2* (LOC123222807, LOC123215944, LOC123213453), abscisic acid receptor *PYR/PYL* family (*PYR/PYL*, LOC123203846), and *CTR1* (LOC123203777) showed increased expression. In addition to the above genes, others (*MYC2*, *PYR/PYL*, *EIN3*) showed no or low differential expression at W2 and W4.

To further understand the expression of MAPK-and plant hormone-related pathway genes, we screened several significantly different expressed genes related to the signal transduction pathway including *MYC2* (*transcription factor MYC2*, LOC123215681), *ER* (*ethylene receptor*, LOC123217018), *PYR/PYL* (abscisic acid receptor PYR/PYL family, LOC123223767), and *CTR1* (serine/threonine-protein kinase *CTR1*, LOC123225032). *MYC2*, *PYR/PYL*, and *CTR1* were downregulated, and their expression levels decreased with the extension of heat stress. It is speculated that these three differential genes are involved in activating target genes, interacting with a variety of hormone signaling pathways, and participating in the regulation of plant growth, reproduction, defense, and secondary metabolism, which play important roles in plant growth and development (Richter et al., 2010). In addition, *CTR1* was upregulated in each time period for the three varieties. Interestingly, with the extension of the heat stress period, the expression of *CTR1* first increased and then decreased, reaching a maximum at W4. It is speculated that through the interaction of proteins such as *CTR1* and *EIN2*, the signal is transmitted to downstream transcription factors, and then the gene expression and physiological processes are regulated to resist heat stress.

### 3.7 Identification of candidate genes by WGCNA

The FPKM values of 12,635 DEGs were used to analyze the weighted gene co-expression network and calculate the correlation of modular traits. Seventeen gene modules were identified according to co-expression patterns. Each module was assigned a color and visualized as a hierarchical clustering tree and a gene clustering heatmap (**Supplementary Figures 11, S12**). The built-in extension of Cytoscape was used to identify hub genes and to visualize the gene network.

**Figure 6:**
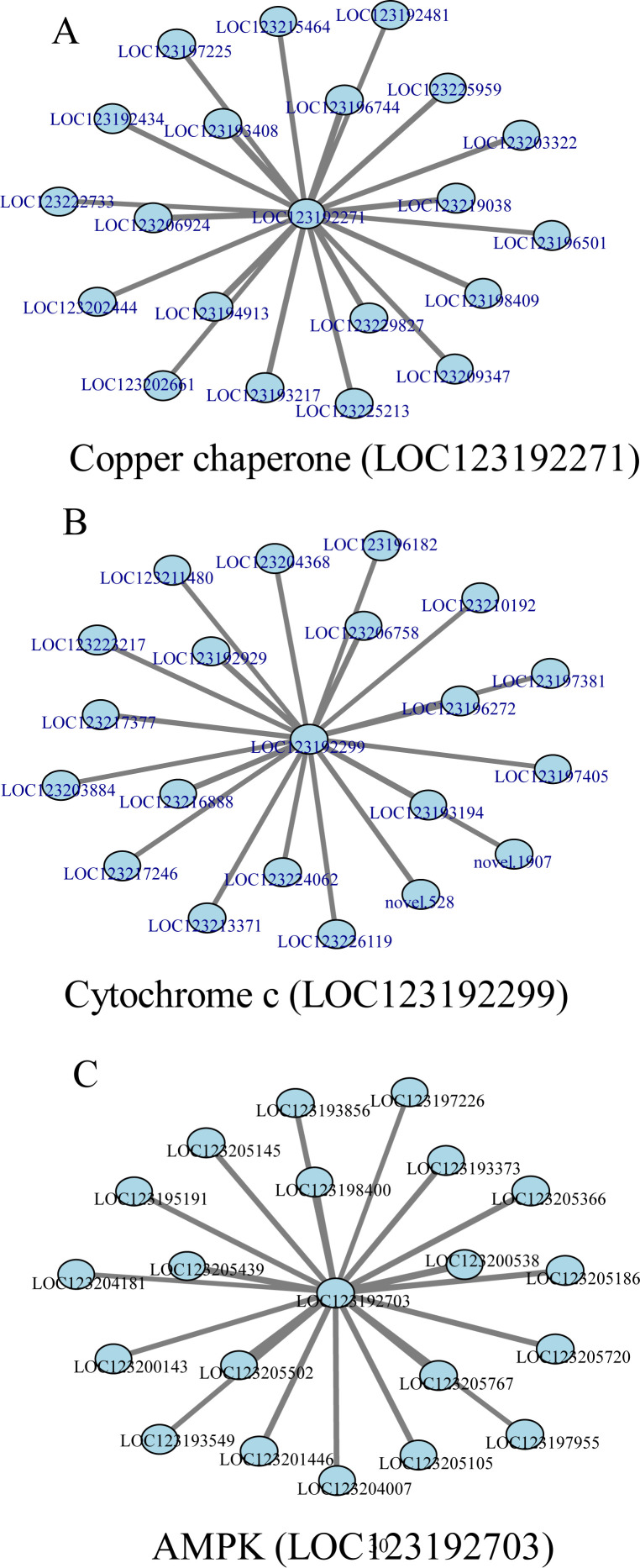
Gene co−expression networks for the three hub genes identified by WGCNA. Networks are shown as hub−and−spoke layouts for **(A)** copper chaperone (LOC123192271), **(B)** cytochrome c (LOC123192299), and **(C)** *AMPK* (LOC123192703). Each hub gene is connected to its top 20 co−expressed neighbors (edges are based on Pearson correlation, |r| > 0.7, p < 0.01). Edge width is proportional to the connection weight (|r|), with thicker lines indicating stronger co−expression. Node labels are gene IDs. The layout was generated using a force−directed algorithm (Prefuse Force Directed in Cytoscape). All three networks were constructed from the combined 36 samples (3 cultivars × 4 treatments × 3 replicates).

Through network co-association, one hub gene was identified from each of 17 modules. These hub genes were: LOC123209963 in the black module, LOC123207767 in the blue module, LOC123224931 in the brown module, LOC123204543 in the cyan module, LOC123206257 in the green module, *novel.469* in the green-yellow module, LOC123207495 in the grey60 module, LOC123192703 in the lightcyan module, LOC123216537 in the magenta module, LOC123192271 in the midlight blue module, LOC123223219 in the pin module, and LOC123218008 in the pink module, LOC123195576 in red module, LOC123208481 in salmon module, LOC123192299 in tan module, LOC123218697 in turquoise module, and LOC123224257 in yellow module (**Supplementary Figure 13**). We further identified the key candidate genes involved in acute heat stress response in mangoes from the 16 hub genes (**Supplementary Figure 14**). Finally, three key candidate genes involved in the heat stress resistance mechanism of mango were identified: 5’-AMP-activated protein kinase (AMPK, LOC123192703) from the lightcyan module (**Figure 6A**), copper chaperone (LOC123192271) from the midlight blue module (**Figure 6B**), and cytochrome c (LOC123192299) from the tan module (**Figure 6C**). These are the three highly related modules containing the key genes. Of these 17 hub genes, three were selected for further integrated analysis based on their high connectivity and annotation relevance to stress responses.

### 3.8 Key candidate genes underlying mango defense mechanisms against heat stress

**Figure 7:**
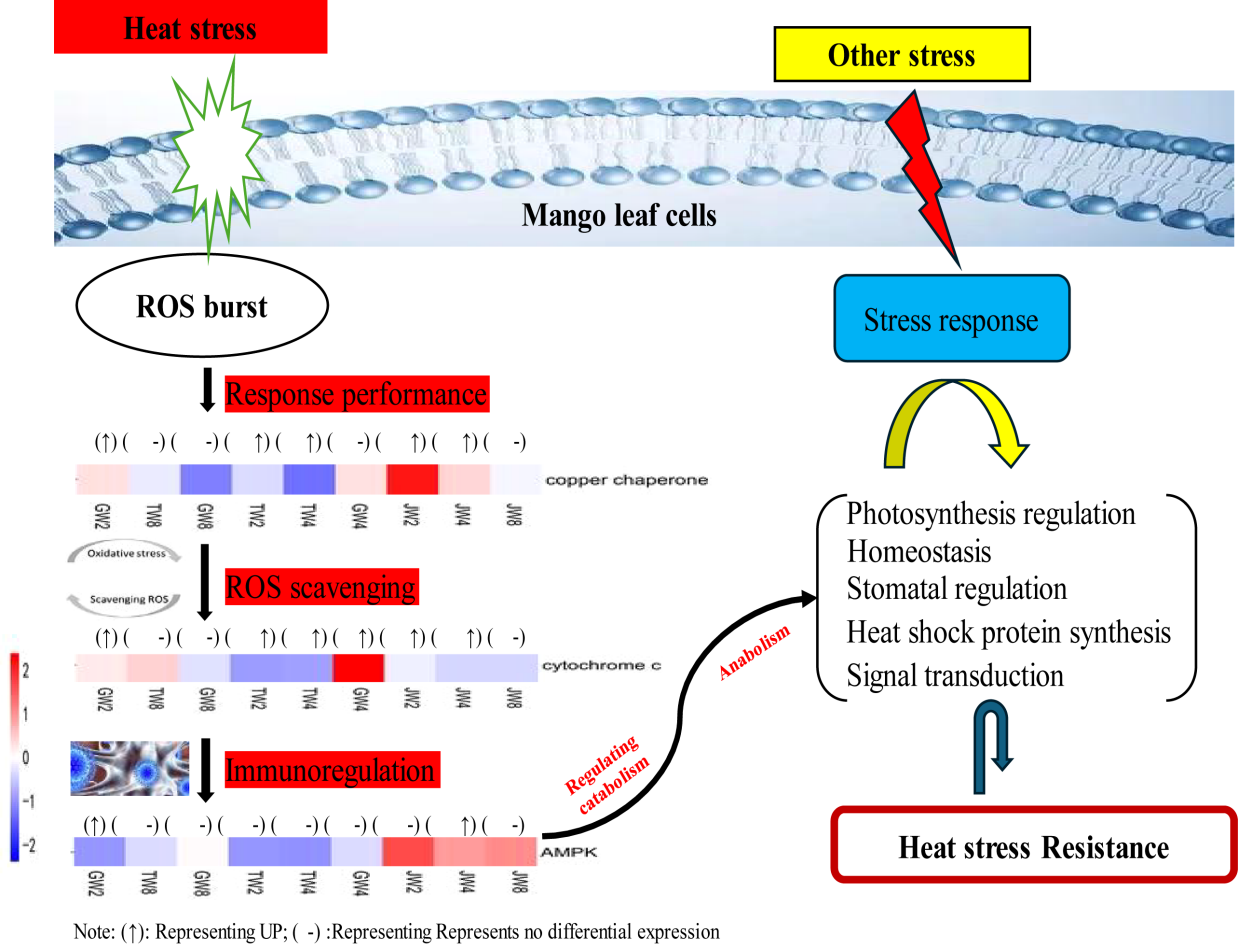
Schematic diagram of regulation of three key genes.

The important candidates identified were *AMPK* (LOC123192703) from the lightcyan module, copper chaperone (LOC123192271) from the midlight blue module, and cytochrome c (LOC123192299) from the tan module. Copper chaperone (LOC123192271) is associated with SOD activity, participates in reactive oxygen species scavenging, and may play a role in plant stress resistance. The three varieties showed upregulated expression at W2, with the highest expression observed in JH. With increasing temperature, its expression level gradually decreased, and no differential expression was detected at W8. The expression of copper chaperones in JH at W2 and W4 was higher than that in the other varieties. Cytochrome c (LOC123192299) exerts immunomodulatory and enzymatic effects on the oxidation and reduction of plant tissues. It also participates in electron transfer in cells, especially in the cellular respiratory chain. Over time under heat stress, the expression levels of cytochrome c in the three cultivars were upregulated at W2 and W4. In particular, the expression patterns and levels in JH and GQ were similar during the first two time periods (W2 and W4). It is speculated that cytochrome c participates in plant metabolism, promotes nutrient absorption, maintains normal physiological activities, and contributes to heat stress resistance by accelerating enzymatic reactions. *AMPK* (LOC123192703) is involved in the regulation of catabolic and anabolic pathways. *AMPK* expression is associated with genes involved in photosynthesis, homeostasis, and stomatal movement under heat stress. Over time under heat stress, *AMPK* was upregulated only in JH, and its expression level gradually decreased after the initial increase. However, *AMPK* was not differentially expressed in the other two varieties at any time point compared to the control group. *AMPK* may contribute to heat stress resistance by influencing photosynthesis, homeostasis, stomatal regulation, heat shock protein synthesis, and signal transduction through metabolic, catabolic, and anabolic pathways. Based on their expression patterns, expression levels, and potential roles in the acute heat stress response and plant defense mechanisms, these three genes were selected as candidate acute heat stress response genes in mango (**Figure 7**).

### 3.9 Integrated correlation network analysis of key metabolites and genes

**Figure 8:**
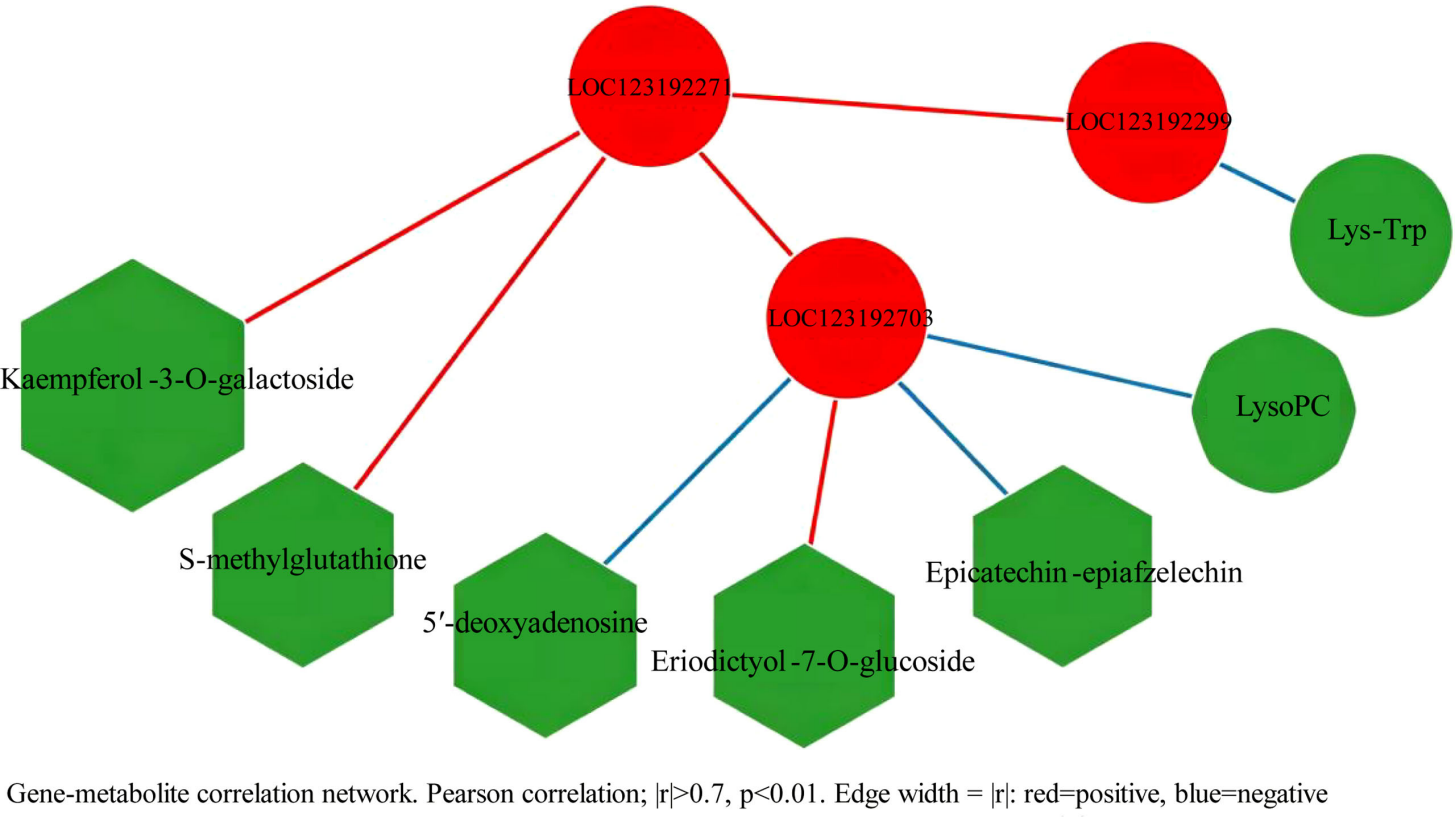
Integrated gene–metabolite correlation network under acute heat stress. Pearson correlation coefficients (r) were calculated between three hub genes (*AMPK* LOC123192703, cytochrome c LOC123192299, copper chaperone LOC123192271) and seven differential metabolites across all 36 samples. Only significant correlations (|r| > 0.7, p < 0.01) are shown. Red edges indicate positive correlation, blue edges indicate negative correlation. Edge width is proportional to |r|. Node shapes: ellipses = genes, hexagons = metabolites. Metabolite full names: kaempferol−3−O−galactoside, S−methylglutathione, 5′−deoxyadenosine, eriodictyol−7−O−glucoside, lysoPC, epicatechin−epiafzelechin, and Lys−Trp. Data are from 36 samples (3 cultivars × 4 treatments × 3 replicates). The exact r and p values for all pairs are provided in **Supplementary Table 3**.

To explore the functional coupling between metabolites and transcripts, we performed Pearson correlation analysis between the three WGCNA−identified hub genes (copper chaperone LOC123192271, cytochrome c LOC123192299, and *AMPK* LOC123192703) and all differential metabolites across the 36 samples. Only significant correlations with |r| > 0.7 and p < 0.01 were retained for network visualization. The resulting correlation network (**Figure 8**; **Table 3**) includes 10 nodes (3 genes and 7 metabolites) and 7 significant edges. Specifically, the copper chaperone (LOC123192271) showed a strong positive correlation with kaempferol−3−O−galactoside (r = 0.87, p < 0.001) and a negative correlation with S−methylglutathione (r = -0.79, p < 0.001). Cytochrome c (LOC123192299) was positively correlated with 5′−deoxyadenosine (r = 0.73, p < 0.001). *AMPK* (LOC123192703) exhibited the most extensive associations, with strong positive correlations with eriodictyol−7−o−glucoside (r = 0.79), epicatechin−epiafzelechin (r = 0.81), lysophosphatidylcholine (lysoPC, r = 0.89), and Lys−Trp (r = 0.84) (all p < 0.001). Edge width in the network is proportional to |r|, and edge color indicates positive (red) or negative (blue) correlation. The full correlation matrix, including all metabolite−gene pairs with r and p values, is provided in **Supplementary Table 3**.

**Table 3:** Correlation coefficient matrix (3 genes × 7 metabolites).

Gene/metabolite	LOC123192271 (copper chaperone)	LOC123192299 (cytochrome c)	LOC123192703 (*AMPK*)
mws0913 (Kaempferol-3-O-galactoside)	0.87	-0.12	0.56
mws0582 (S-methylglutathione)	-0.79	0.23	-0.18
MWSmce116 (5’-deoxyadenosine)	0.34	0.73	0.16
MWS20145 (Eriodictyol-7-O-glucoside)	0.48	-0.08	0.79
pmb3114 (Epicatechin-epiafzelechin)	0.62	0.01	0.81
pmp001273 (LysoPC)	0.41	-0.21	0.89
MW0108107 (Lys-Trp)	0.39	-0.15	0.84

Bolded correlation coefficients indicate |r| > 0.7 and p < 0.001 (with n = 36, the threshold for p < 0.001 is |r| > 0.518). All p−values were between 1e−6 and 1e−12.

### 3.10 Consistency of transcriptome-metabolome regulation across additional hub genes

To further extend the integration beyond the three genes shown in **Figure 8**, we examined three additional hub genes identified by WGCNA (LOC123209963, LOC123207767, LOC123224931) and assessed whether their expression changes under heat stress were directionally concordant with the accumulation patterns of representative metabolites from their respective pathways. As summarized in **Table 4**, all three genes showed consistent upregulation with the corresponding metabolites in the heat-tolerant cultivar JH. This pathway-level concordance supports the robustness of our multi-omics dataset and indicates that the coordinated regulation is not limited to the three focused genes.

**Table 4:** Concordance of regulation direction between selected additional hub genes and representative metabolites in the heat-tolerant cultivar JH (8 h heat stress vs control).

Gene ID	KEGG pathway	Log_2_FC (gene)	Representative metabolite	Log_2_FC (metabolite)	Concordant
LOC123209963	Flavonoid biosynthesis	↑ (+2.1)	Kaempferol-3-O-galactoside	↑ (+1.8)	Yes
LOC123207767	Linoleic acid metabolism	↑ (+1.5)	Trihydroxy-10,15-octadecadienoic acid	↑ (+1.2)	Yes
LOC123224931	Plant hormone signal transduction	↑ (+1.9)	Abscisic acid	↑ (+1.4)	Yes

Genes were selected from the 16 WGCNA hub genes (**Supplementary Figure 14**) other than the three shown in **Table 3**. log_2_FC values are from the comparison JW8 vs JC (Jinhuang cultivar, 8 h heat stress vs control). Concordant indicates that gene and metabolite are both upregulated (positive log_2_FC) or both downregulated (negative log_2_FC).

## 4 Discussion

Global warming has increased the frequency of extreme heat stress events (Kan et al., 2023). Similar to most crops, mango responds to heat stress by activating a complex network of physiological, biochemical, and molecular regulations (Wang et al., 2023; Yu et al., 2023). Through a multi-dimensional analysis of three mango cultivars under acute heat stress, this study not only confirmed some typical responses associated with plant acute heat stress response but, more importantly, systematically integrated physiological damage indicators, key gene co-expression modules, and dynamic associations with specific metabolites for the first time in mango, thereby proposing a hypothetical hierarchical framework for the heat stress response.

Oxidative damage triggered by heat stress is a core cause of plant mortality. In this study, the MDA content significantly increased in all three cultivars with prolonged stress, clearly indicating excessive accumulation of ROS and consequent membrane system damage (Qiu et al., 2019). However, the trajectory of physiological and metabolic responses among cultivars revealed crucial differences in their acute heat stress response. In the more heat-tolerant cultivar (JH), the antioxidant enzyme system (SOD, POD, CAT) maintained a more effective and prolonged activation during the early stages of stress, consistent with previous findings (Lin et al., 2022). JH also showed stable and sustained increases in mean differential metabolite intensity across 12 key metabolic pathways. In contrast, the enzyme activity in the GQ showed a rapid decline after an initial peak, leading to a faster loss of ROS-scavenging capacity, and core defense metabolites were weakly activated. Similarly, the accumulation pattern of the osmolyte proline (Pro) exhibited cultivar specificity (Zhang et al., 2020). This pattern is consistent with earlier reports in other fruit crops. For instance, in tomato and wheat, heat-tolerant genotypes also exhibit sustained antioxidant enzyme activity, whereas sensitive genotypes show a sharp decline after an initial increase (Zhou et al., 2022; Mustafa et al., 2021). These results are consistent with the widely accepted view that “antioxidant defense and osmotic adjustment are fundamental to plant heat tolerance” but, more significantly, translate and metabolic physiological phenotypic differences into quantifiable metrics, providing clear phenotypic anchors for subsequent gene mining. Our data suggests that superior acute heat stress response capacity may lie not in completely avoiding damage but in sustaining the duration and efficiency of the coordinated physiological and metabolic defense system activity.

To elucidate the molecular basis underlying these physiological differences, we employed Weighted Gene Co-expression Network Analysis (WGCNA), successfully screened three key genes highly associated with the acute heat stress response from the complex transcriptome data: a copper chaperone (LOC123192271), cytochrome c (LOC123192299), and *AMPK* (LOC123192703). Their significance lies not merely in their identification but in their collective functional implication of three core aspects potentially involved in the regulatory network of the acute heat stress response: redox homeostasis, energy metabolism and programmed cell death, and global energy signaling. Copper chaperone (LOC123192271): As a member of the *ATOX1* family, it is primarily involved in copper ion homeostasis. This functional identification of a plant copper chaperone was first established through its yeast homolog ATX1 (Himelblau et al., 1998). Under heat stress, copper ions serve as cofactors for antioxidant enzymes (SOD) yet are also potential catalysts for the Fenton reaction. We speculate that this gene may be involved in regulating intracellular copper distribution, potentially influencing copper/zinc superoxide dismutase (Cu/Zn-SOD) while reducing oxidative damage caused by free copper ions (María-Eugenia et al., 2020). However, direct experimental validation is needed to confirm this regulatory role. This result is consistent with studies in *Arabidopsis thaliana*, where overexpression of the copper chaperone *ATOX1* enhanced Cu/Zn-SOD activity and improved oxidative stress tolerance (María-Eugenia et al., 2020). Our observation that JH exhibited higher and more sustained copper chaperone expression compared to TN and GQ provides further support for its candidate positive role in heat tolerance. This suggests that the acute heat stress response may involve the fine management of essential trace elements. Cytochrome c (LOC123192299): Traditionally viewed as a key component of the mitochondrial respiratory chain and photosynthetic electron transport chain (Andronis and Roubelakis-Angelakis, 2010). Its high expression under heat stress is consistent with the critical importance of maintaining unimpeded electron flow for cellular energy (ATP) production and carbon assimilation. More notably, cytochrome c is also a key signaling molecule in plant programmed cell death. Its expression pattern could represent a “trade-off”: enhancing energy production under mild stress, and under severe stress, potentially initiating localized programmed cell death to protect the whole plant. *AMPK* (LOC123192703): This represents a particularly intriguing finding. *AMPK* acts as a central energy and stress-sensing kinase in plants (Herzig and Shaw, 2018). Our analysis suggests a potential role for AMPK in the mango heat stress response. This is consistent with the established role of *AMPK* as a master energy sensor under various abiotic stresses in mammals and plants (Herzig and Shaw, 2018). In *Arabidopsis*, AMPK homologs (SnRK1 complexes) are known to be associated with the regulation of catabolic and anabolic pathways under energy deprivation (Baena-González et al., 2007). However, to our knowledge, this is the first study to implicate *AMPK* specifically in acute heat stress response in mango. *AMPK* was only upregulated in JH, which underscores its potential as a candidate determinant of cultivar-specific acute heat stress response. During energy crisis (ATP depletion induced by heat stress), *AMPK* may be involved in reallocating resources by inhibiting anabolism and promoting catabolism (autophagy) (Yan et al., 2017; Liu et al., 2019). These correlative data provide molecular clues for understanding the “survival over growth” strategy of plants under heat stress, but causal relationships remain to be established.

The integrated correlation analysis (presented in section 3.10) provides statistically supported associations between the three hub genes and specific metabolite classes. This approach goes beyond independent interpretation of the two omics layers and reveals a coordinated correlation (**Figure 8**). Specifically, the copper chaperone (LOC123192271) showed a strong positive correlation with the flavonol kaempferol−3−O−galactoside (r = 0.87, p < 0.001), suggesting a potential link to flavonoid metabolism and antioxidant defense under heat stress. In contrast, it exhibited a negative correlation with S−methylglutathione (r = -0.79, p < 0.001), raising the possibility of a trade−off between glutathione−dependent and flavonoid−dependent ROS scavenging pathways. Cytochrome c (LOC123192299) was strongly correlated with 5′−deoxyadenosine (r = 0.73, p < 0.001), implying a possible involvement in methylation cycles or stress−related nucleotide signaling. *AMPK* (LOC123192703) displayed the most extensive metabolite associations, with strong positive correlations with eriodictyol−7−O−glucoside (r = 0.79), epicatechin−epiafzelechin (r = 0.81), lysoPC (r = 0.89), and the dipeptide Lys−Trp (r = 0.84) (all p < 0.001). Taken together, these correlations are consistent with a model in which AMPK is associated with integrating energy status (lipid remodeling), redox balance (flavonoids), and amino acid metabolism during the acute heat stress response. The complete list of significant correlations is available in **Supplementary Table 3**. Collectively, these gene−metabolite correlations support the candidate relevance of the three hub genes and are consistent with a hypothetical model in which *AMPK* may coordinate broad metabolic adjustments, while copper chaperone and cytochrome c may fine−tune specific antioxidant and electron transfer pathways. To our knowledge, this is the first study to apply network−based integration of metabolomic and transcriptomic data to dissect acute heat stress responses in mango, providing testable hypotheses for future functional studies.

Based on the above analysis, we propose a hypothetical model for the mango heat stress response. This model suggests that the heat stress response may be a coordinated process unfolding in temporal sequence and logical hierarchy: First, Rapid Sensing and Primary Damage: Heat stress directly causes ROS burst and damage to photosynthetic apparatus, triggering membrane lipid peroxidation (increased MDA) and declined photosynthetic efficiency (Averill-Bates, 2024). Secondly, Physiological Defense Mobilization: Plants activate “firefighting” systems, including the accumulation of antioxidant enzymes (SOD, POD, CAT) and osmolytes (Pro), to contain primary damage. Thirdly, Candidate Core Gene Regulatory Network: At the transcriptional level, *AMPK* shows expression patterns consistent with a role in energy and stress signaling, potentially contributing to coordinating the global response. The copper chaperone is upregulated under heat stress, which may help optimize redox cofactor homeostasis and enhance antioxidant capacity; cytochrome c is modulated, possibly balancing energy supply with potential programmed cell death decisions. Finally, Metabolic Reprogramming Execution: The observed correlations suggest that the regulatory effects of key genes may be executed by influencing specific metabolic pathways (flavonoid synthesis, lipid metabolism, amino acid metabolism), as indicated by the observed gene–metabolite associations. This hypothetical model associates physiological phenotypes, core transcriptional regulators, and functional metabolites into a coherent whole, offering a potential explanation for why JH can more effectively delay wilting, as if it possess more efficient *AMPK* signal activation, more sustained copper chaperone-mediated antioxidant capacity, and a more balanced cytochrome c-related energy versus cell death decision-making. We emphasize that this model is based on correlative data and requires experimental testing.

In summary, many of our physiological observations (increased MDA, Pro, and antioxidant enzyme activities) are consistent with well-established plant heat stress responses documented in species such as tomato, rice, and wheat (Kan et al., 2023). However, the specific combination of key genes (copper chaperone, cytochrome c, *AMPK*) identified through WGCNA, and their association with particular metabolite classes (flavonoids, lipids, amino acid derivatives), represents a novel finding in mango. To our knowledge, no previous study has simultaneously integrated metabolome and transcriptome data to compare heat-contrasting mango cultivars. These results extend earlier work by proposing a hypothetical hierarchical framework that associates physiological damage (MDA), core regulatory genes (*AMPK*, copper chaperone, cytochrome c), and downstream metabolic reprogramming (flavonoid and amino acid pathways) in a single model. Finally, we fully acknowledge the limitations of this study regarding the concept of ‘thermotolerance’. The heat stress protocol used (40 °C for 2-8 h) only captures acute, short−term stress responses, not long−term acclimation or repeated stress exposure that would be required to define true ‘thermotolerance’. Therefore, the mechanisms we describe should be interpreted as early signaling and metabolic adjustments under acute heat stress, rather than sustained adaptive traits. Nevertheless, the rapid physiological and molecular divergence among cultivars observed here provides a strong basis for future studies on long−term heat tolerance. The latter may involve different acclimation mechanisms, such as epigenetic modifications or long-term accumulation of specific proteins. Therefore, our model primarily reflects “acute heat stress response,” while the mechanisms underlying long−term acclimation and acquired thermotolerance remain unclear.

Furthermore, a major limitation of this study is that all regulatory conclusions are based on correlation analyses (transcriptome−metabolome integration, WGCNA, and Pearson correlations). Correlation does not imply causation. Therefore, the proposed functions of the hub genes (copper chaperone, cytochrome c, AMPK) in heat tolerance remain hypothetical until validated by functional experiments such as gene overexpression, knockout, or silencing in mango or model plants. Their expression patterns and specific functions across different mango tissues and developmental stages are also unknown. Another limitation is the absence of independent qPCR validation for the RNA−seq data, including the three hub genes. Due to the exploratory nature of this study, we did not perform qPCR. Nevertheless, the high sequencing quality, replicate consistency, and congruence with metabolomic data support the reliability of our transcriptomic findings. The raw data are publicly available (NCBI SRA: PRJNA1373361) for independent verification. Future studies should validate these hub genes using qPCR and functional assays. Addressing these limitations, future research should conduct long-term, progressive heat stress experiments to compare gene expression and physiological responses under different stress regimes, and employ gene editing (CRISPR-Cas9) or transgenic techniques to functionally validate candidate genes. These efforts will strengthen the proposed model by moving from correlation to mechanism, ultimately providing solid and reliable targets for molecular breeding of heat-tolerant mango.

## 5 Conclusion

This study investigated the divergent acute heat stress responses (40 °C, 2-8 h) among three mango cultivars, with ‘Jinhuang’ being the most tolerant, followed by ‘Tainong No.1’, and ‘Guiqi’ the most sensitive. Integrated metabolomic and transcriptomic analyses revealed that the heat−tolerant JH exhibited sustained antioxidant activity, greater accumulation of protective metabolites, and distinct expression patterns of photosynthesis−and stomatal−related genes. Weighted gene co−expression network analysis identified three key candidate genes-copper chaperone, cytochrome c, and AMPK-which were further validated by significant gene−metabolite correlations (|r| > 0.7, p < 0.001). These findings support a hierarchical model in which AMPK coordinates broad metabolic adjustments while copper chaperone and cytochrome c fine−tune antioxidant and electron transfer pathways. This work provides a theoretical basis for breeding heat−tolerant mango varieties. Further functional validation of these key genes is needed.

## Data Availability

The datasets presented in this study can be found in online repositories. The names of the repository/repositories and accession number(s) can be found in the article/**Supplementary Material**.
