# Supplementary material for: Integrated analysis of metabolome and transcriptome reveals the mechanism of divergent acute heat stress responses in mango cultivars
Source: Front Plant Sci. 2026 Jun 12;17:1805686. doi: 10.3389/fpls.2026.1805686 (PMC13303361; doi:10.3389/fpls.2026.1805686)
Supplement: Supplementary file 1 [file DataSheet1.zip › Supplementary Figure and Table/Supplementary Figure S3.pdf]

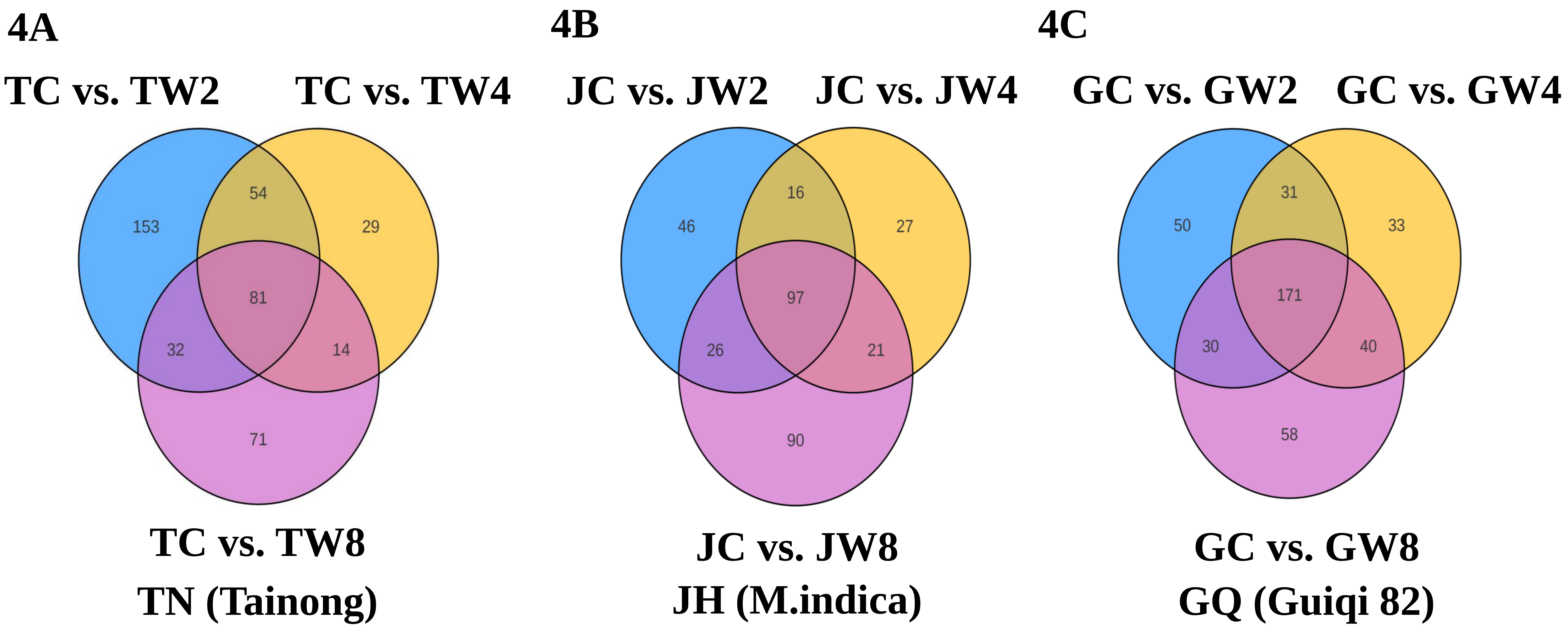
4A
TC vs. TW2
TC vs. TW4
153
54
29
81
32
14
71
TC vs. TW8
TN (Tainong)
4B
JC vs. JW2
JC vs. JW4
46
16
27
97
26
21
90
JC vs. JW8
JH (M.indica)
4C
GC vs. GW2
GC vs. GW4
50
31
33
171
30
40
58
GC vs. GW8
GQ (Guiqi 82)
