# Supplementary material for: Integrated analysis of metabolome and transcriptome reveals the mechanism of divergent acute heat stress responses in mango cultivars
Source: Front Plant Sci. 2026 Jun 12;17:1805686. doi: 10.3389/fpls.2026.1805686 (PMC13303361; doi:10.3389/fpls.2026.1805686)
Supplement: Supplementary file 1 [file DataSheet1.zip › Supplementary Figure and Table/Supplementary Figure S8.pdf]

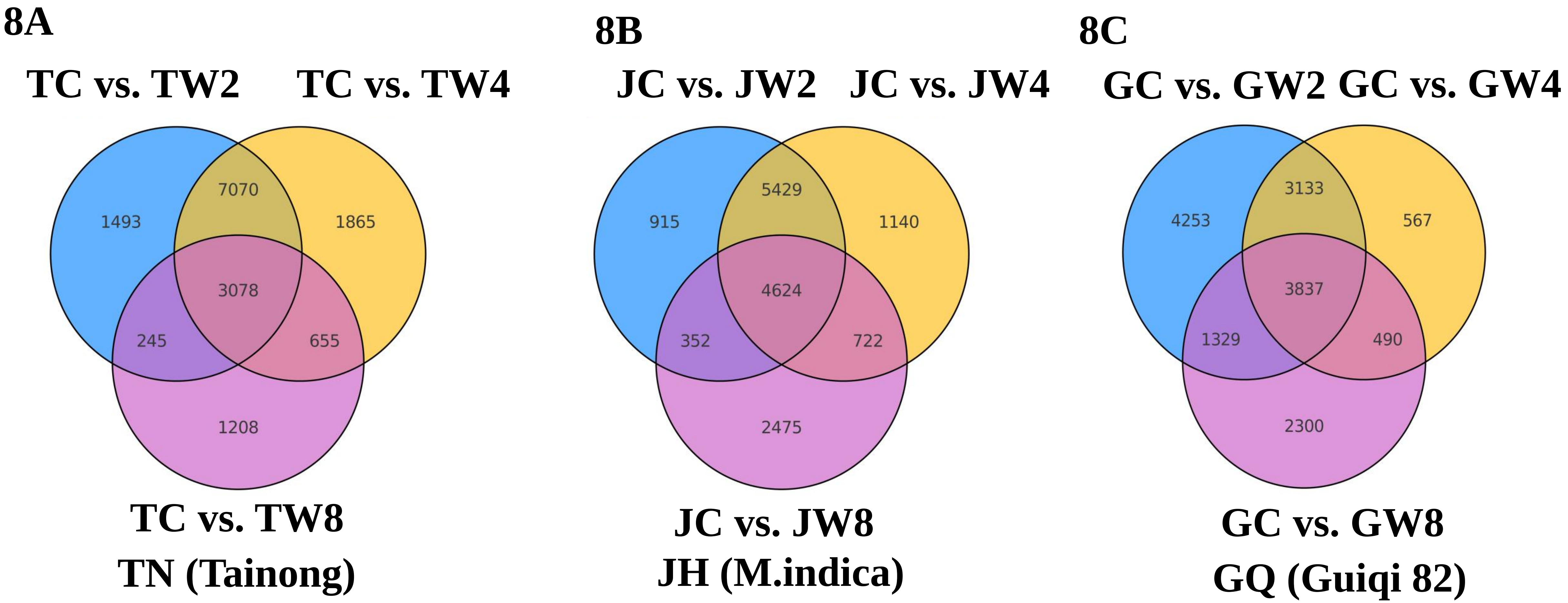
8A
TC vs. TW2
TC vs. TW4
1493
7070
1865
3078
245
655
1208
TC vs. TW8
TN (Tainong)
8B
JC vs. JW2
JC vs. JW4
915
5429
1140
4624
352
722
2475
JC vs. JW8
JH (M.indica)
8C
GC vs. GW2
GC vs. GW4
4253
3133
567
3837
1329
490
2300
GC vs. GW8
GQ (Guiqi 82)
