# Supplementary figures and images for: Integrated analysis of metabolome and transcriptome reveals the mechanism of divergent acute heat stress responses in mango cultivars
Source: Front Plant Sci. 2026 Jun 12;17:1805686. doi: 10.3389/fpls.2026.1805686 (PMC13303361; doi:10.3389/fpls.2026.1805686)

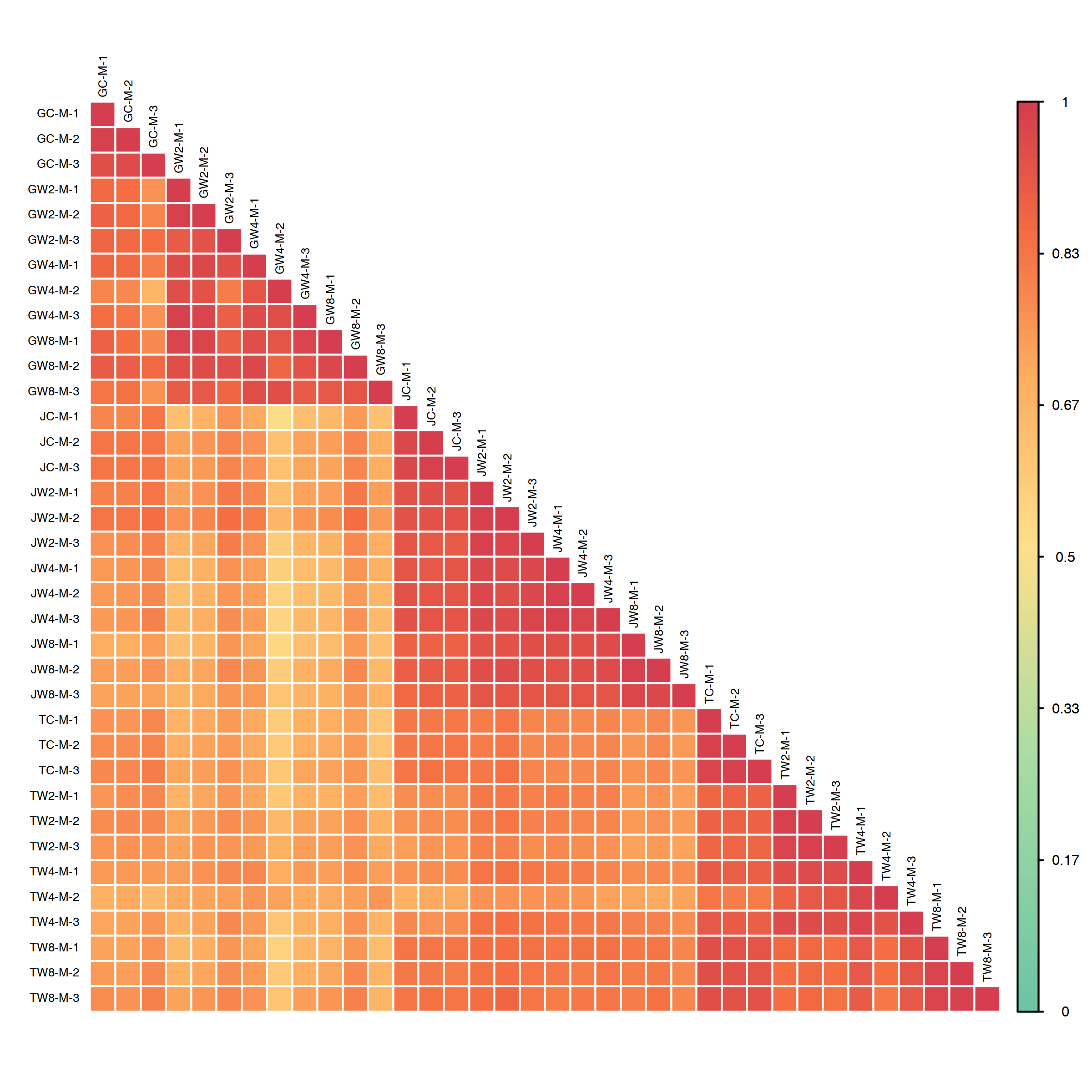

Supplement: Supplementary file 1 [file DataSheet1.zip › Supplementary Figure and Table/Supplementary Figure S1.png]

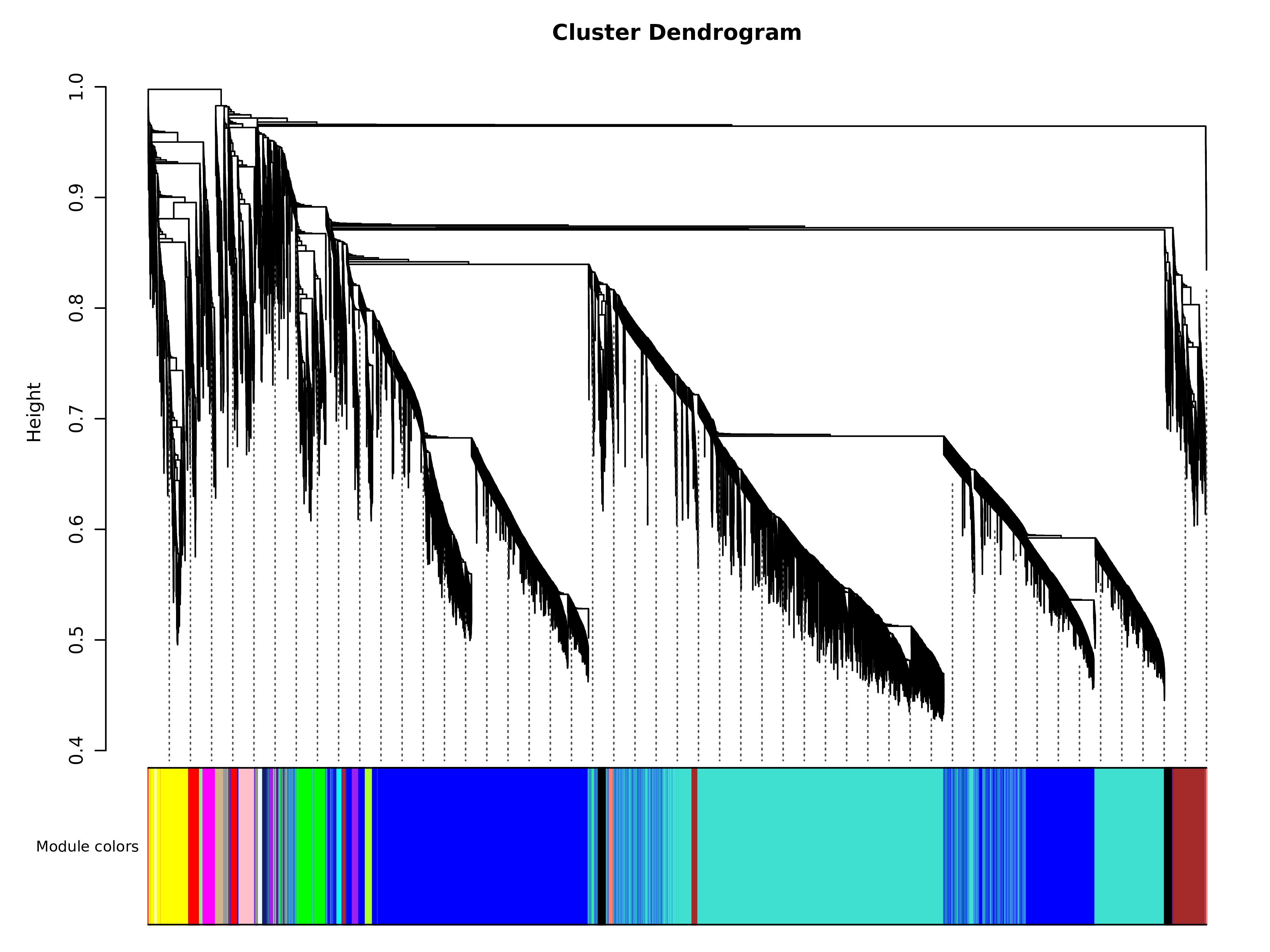

Supplement: Supplementary file 1 [file DataSheet1.zip › Supplementary Figure and Table/Supplementary Figure S12.png]

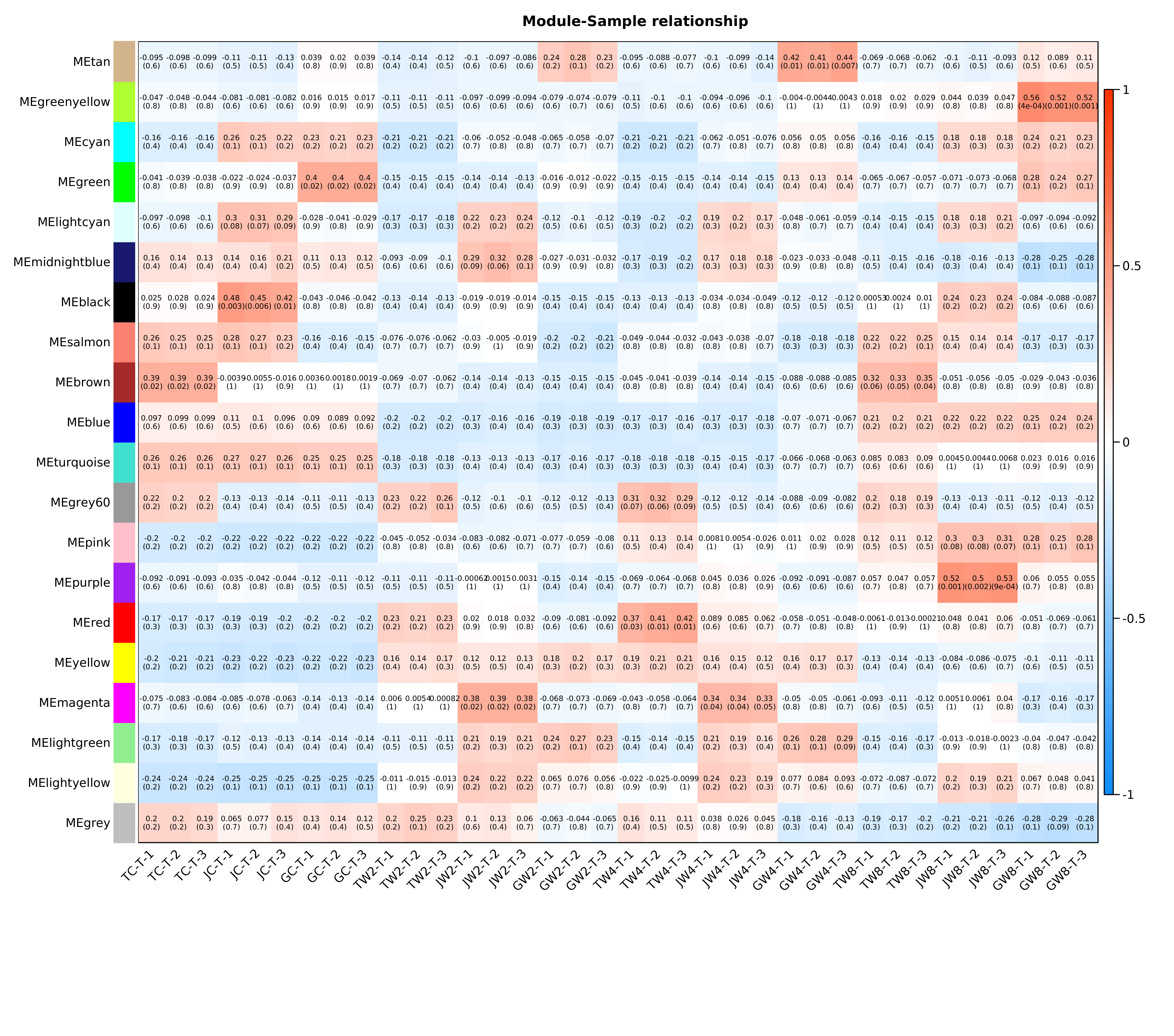

Supplement: Supplementary file 1 [file DataSheet1.zip › Supplementary Figure and Table/Supplementary Figure S13.png]

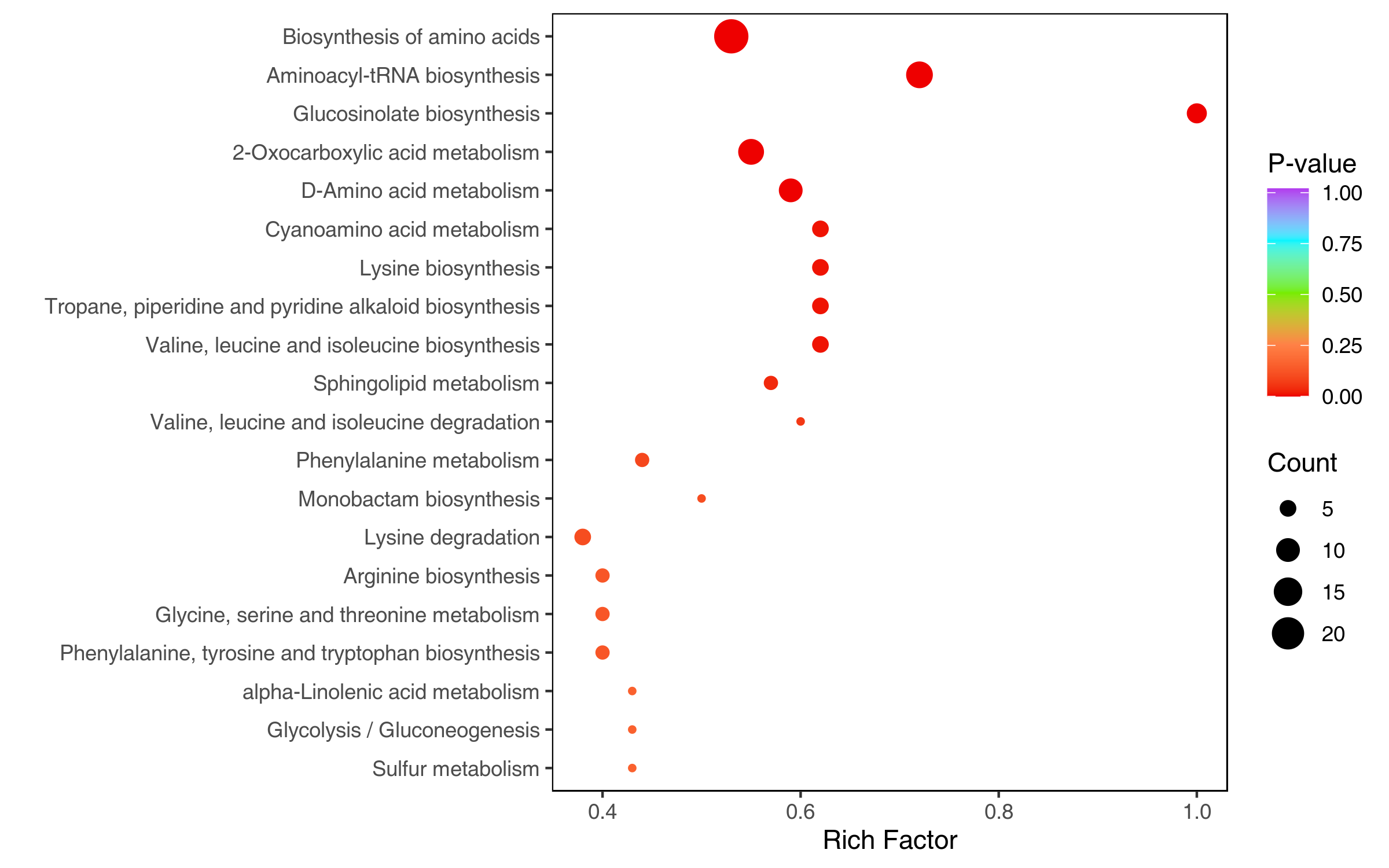

Supplement: Supplementary file 1 [file DataSheet1.zip › Supplementary Figure and Table/Supplementary Figure S4/Guiqi/GC-M_vs_GW4-M_KEGG_Enrichment.png]

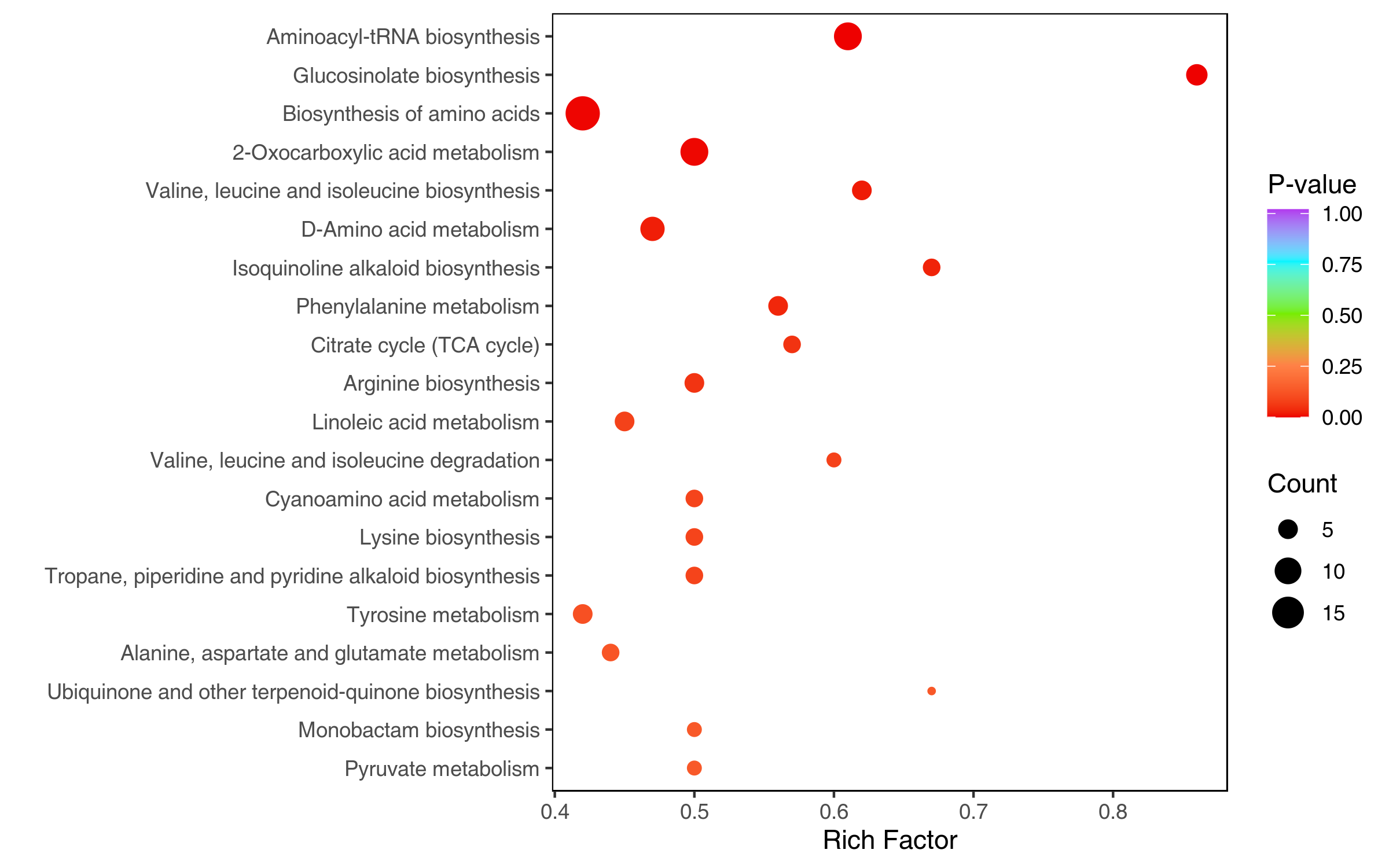

Supplement: Supplementary file 1 [file DataSheet1.zip › Supplementary Figure and Table/Supplementary Figure S4/Guiqi/GC-M_vs_GW8-M_KEGG_Enrichment.png]

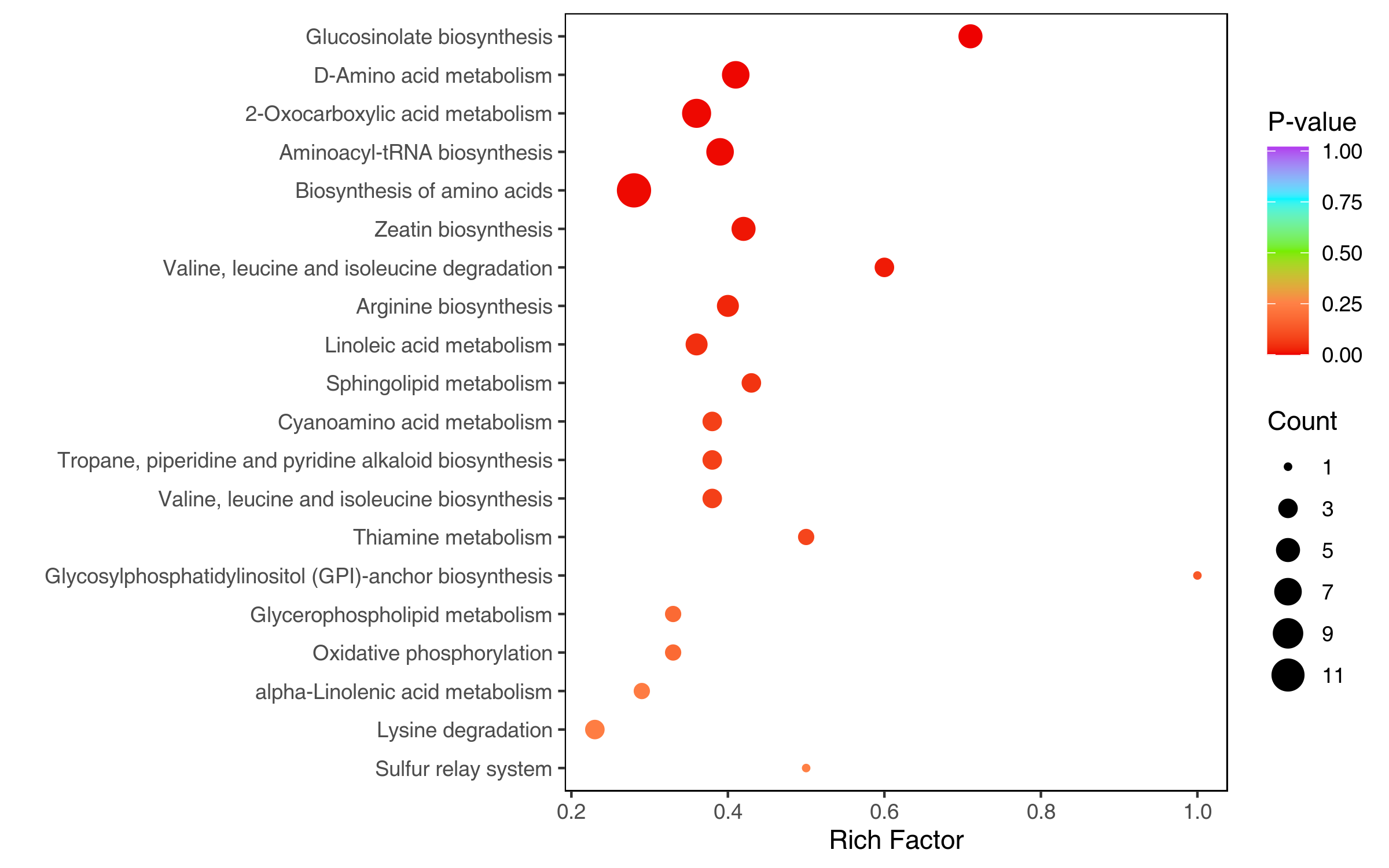

Supplement: Supplementary file 1 [file DataSheet1.zip › Supplementary Figure and Table/Supplementary Figure S4/M.indica/JC-M_vs_JW4-M_KEGG_Enrichment.png]

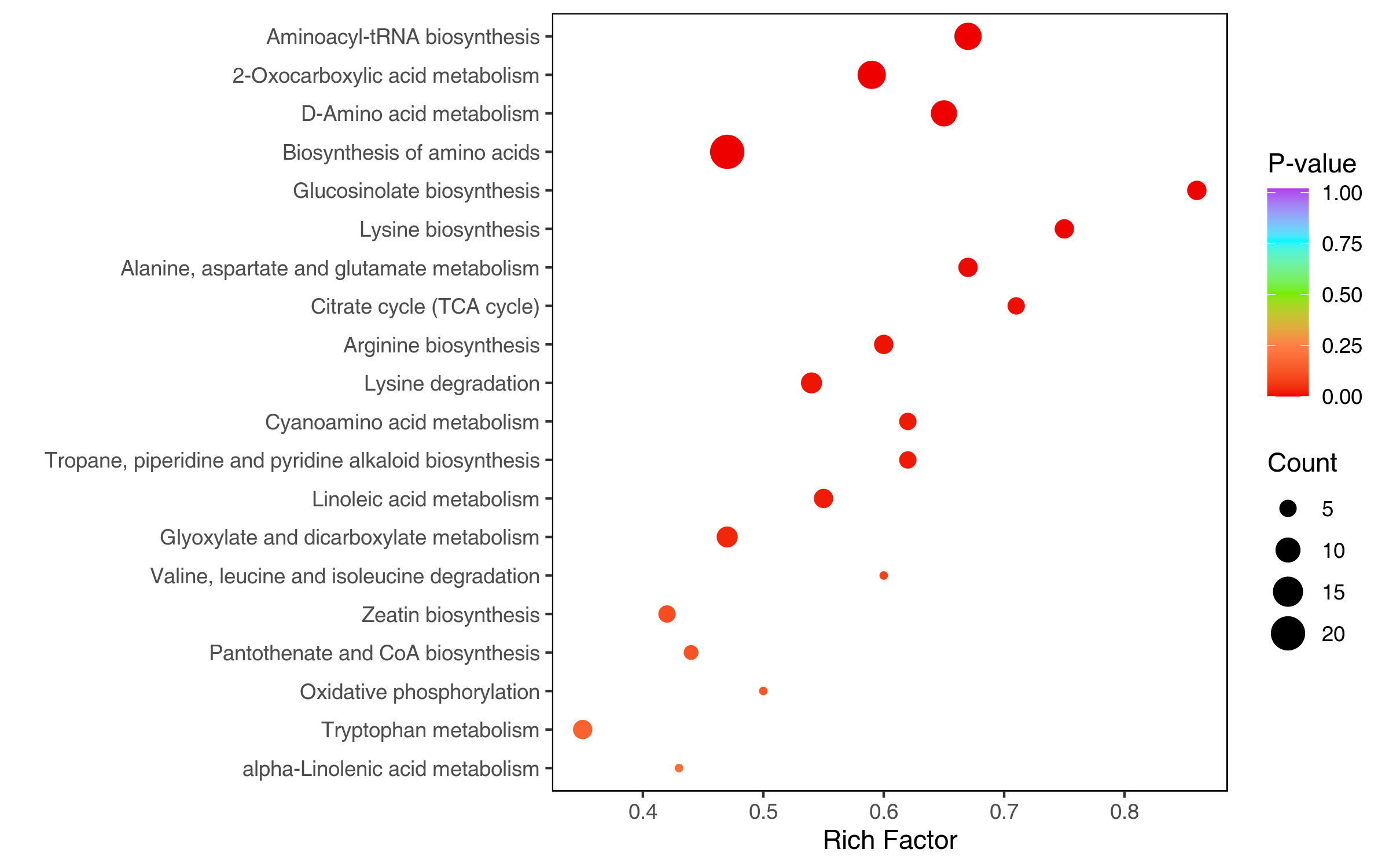

Supplement: Supplementary file 1 [file DataSheet1.zip › Supplementary Figure and Table/Supplementary Figure S4/M.indica/JC-M_vs_JW8-M_KEGG_Enrichment.png]

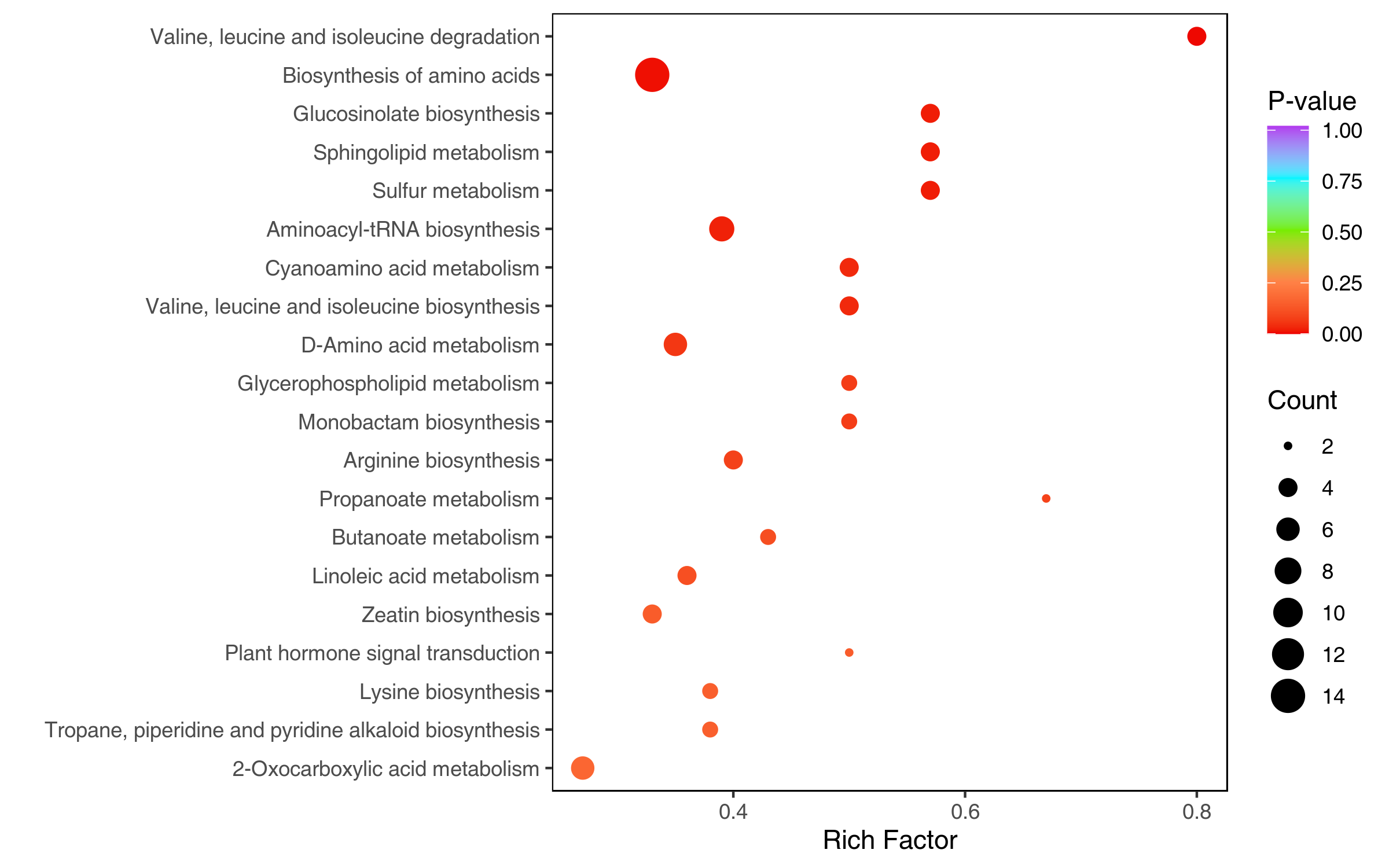

Supplement: Supplementary file 1 [file DataSheet1.zip › Supplementary Figure and Table/Supplementary Figure S4/Tainong/TC-M_vs_TW4-M_KEGG_Enrichment.png]

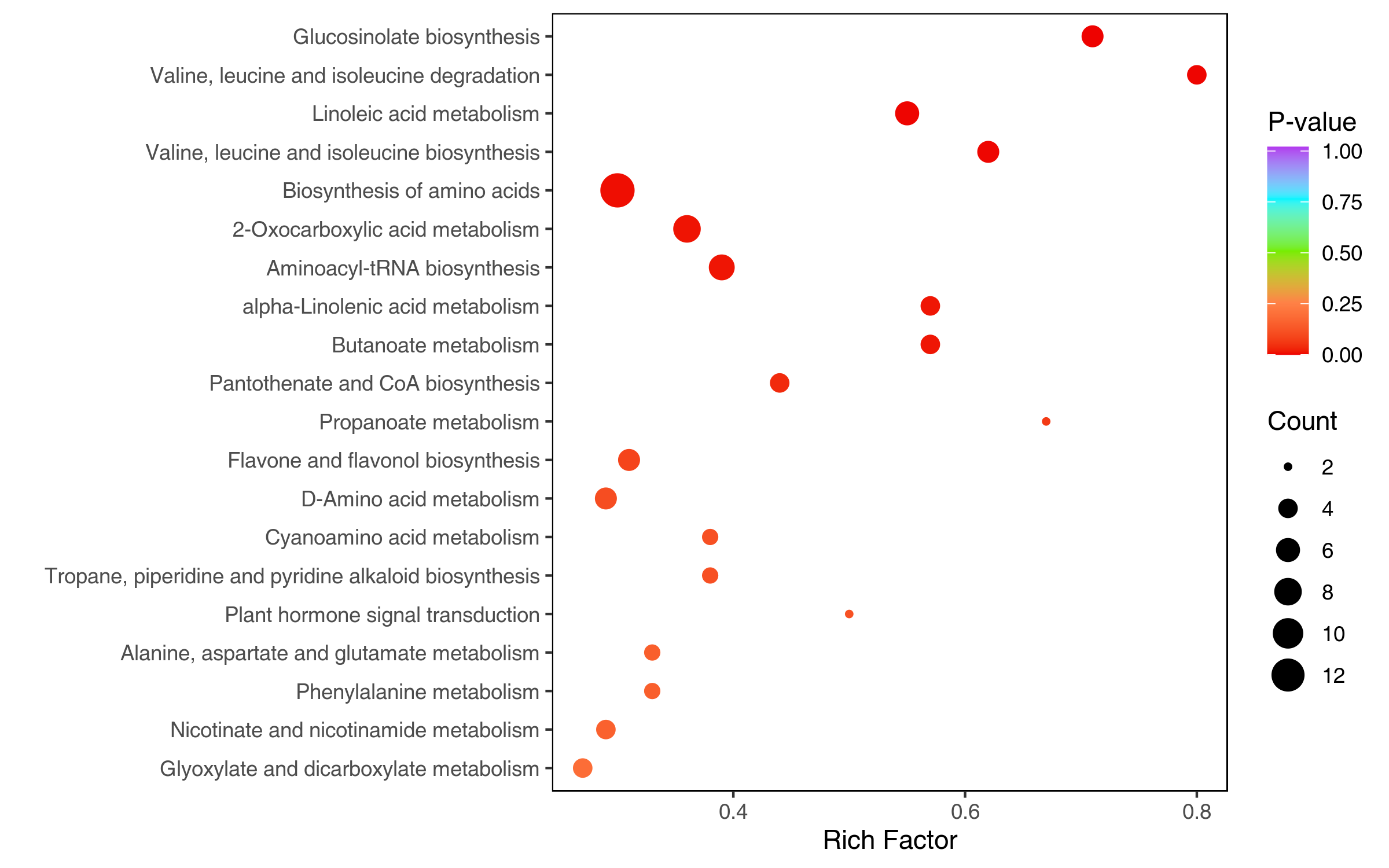

Supplement: Supplementary file 1 [file DataSheet1.zip › Supplementary Figure and Table/Supplementary Figure S4/Tainong/TC-M_vs_TW8-M_KEGG_Enrichment.png]

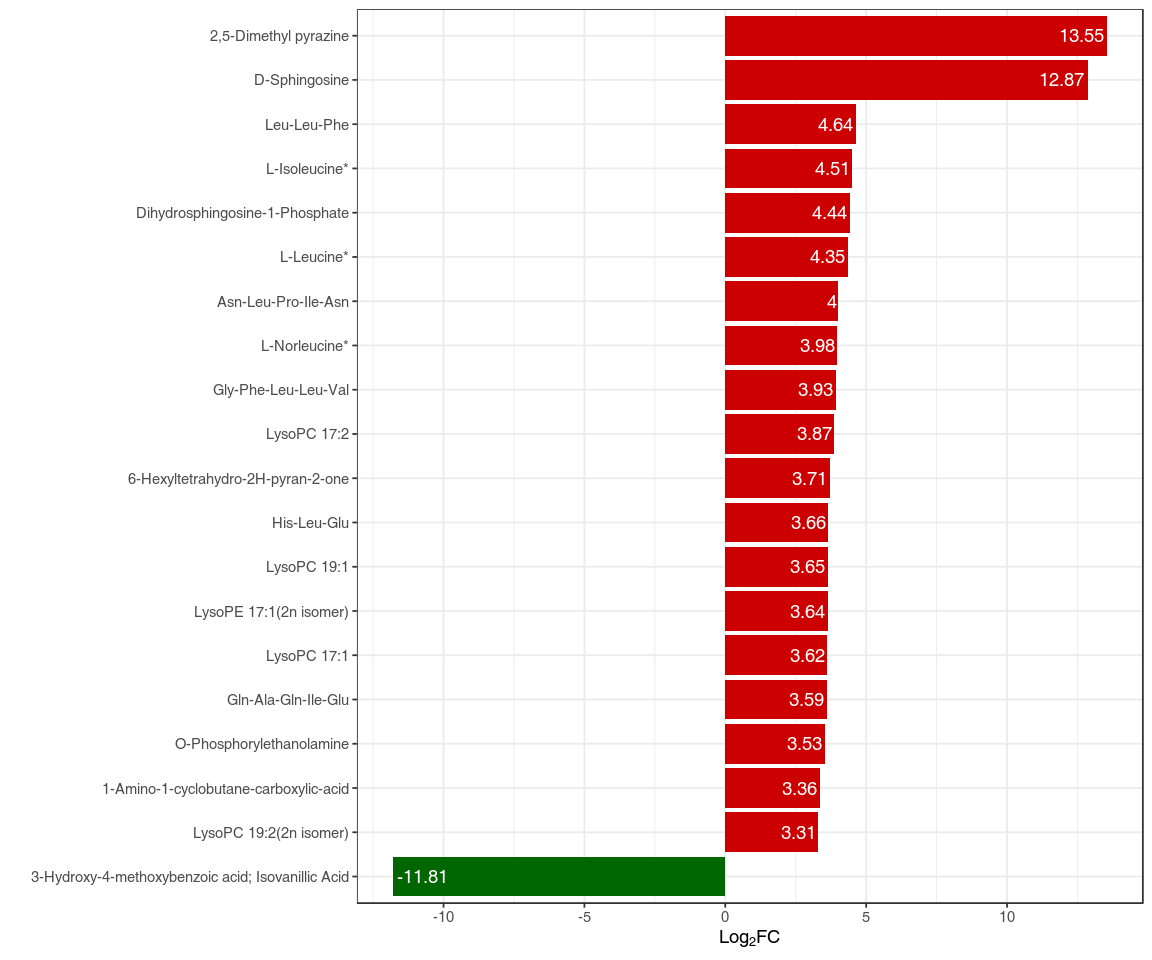

Supplement: Supplementary file 1 [file DataSheet1.zip › Supplementary Figure and Table/Supplementary Figure S5/Guiqi/GC-M_vs_GW4-M_TopFcBarChart_Compounds.png]

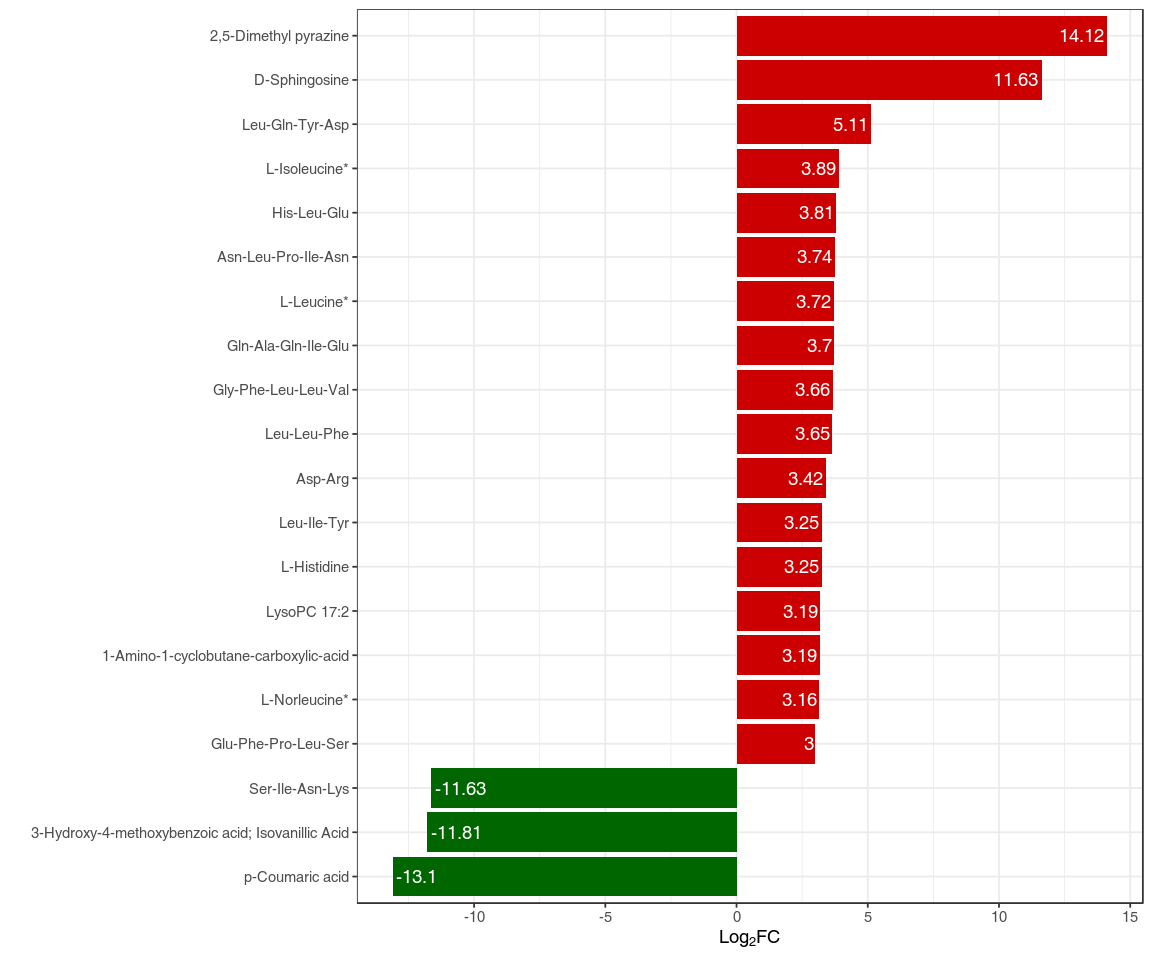

Supplement: Supplementary file 1 [file DataSheet1.zip › Supplementary Figure and Table/Supplementary Figure S5/Guiqi/GC-M_vs_GW8-M_TopFcBarChart_Compounds.png]

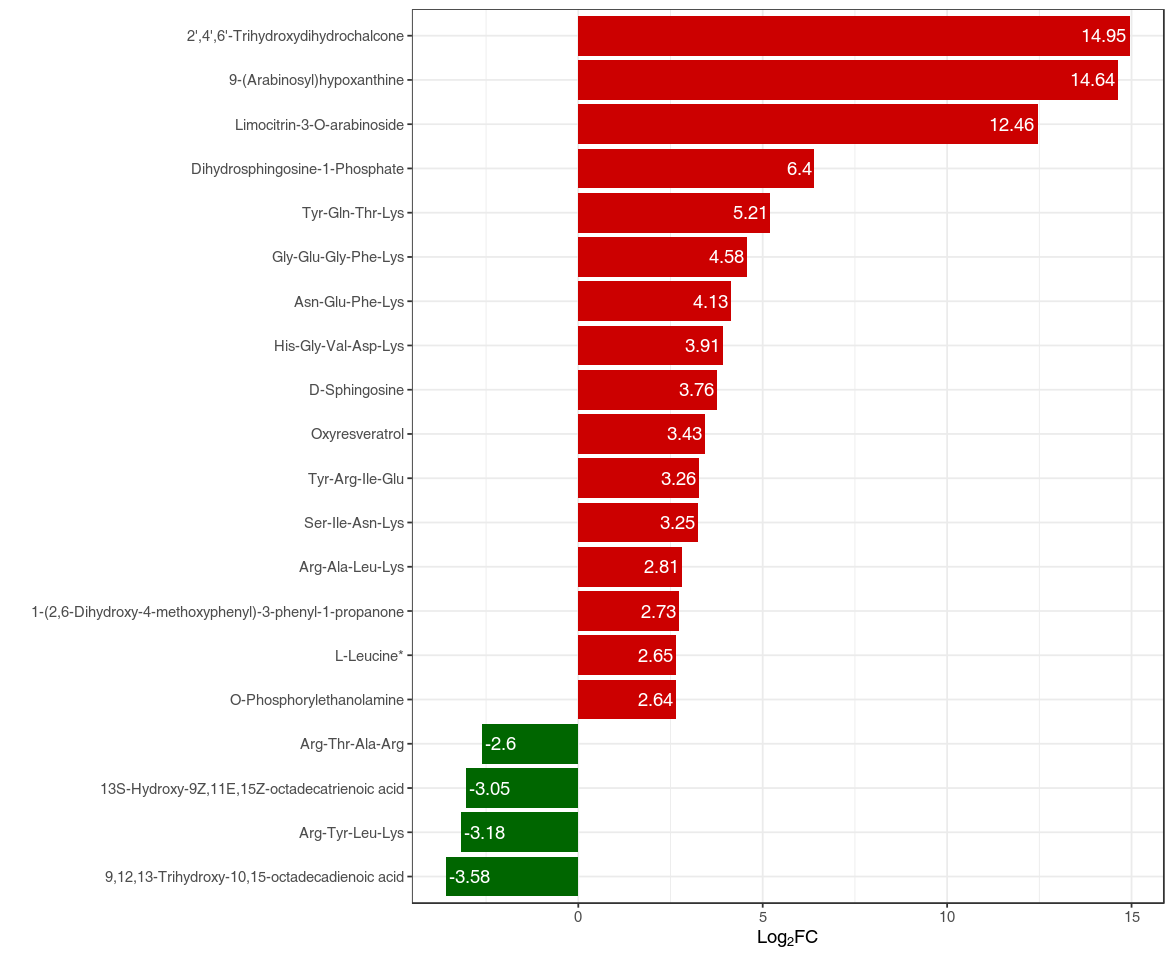

Supplement: Supplementary file 1 [file DataSheet1.zip › Supplementary Figure and Table/Supplementary Figure S5/M.indica/JC-M_vs_JW4-M_TopFcBarChart_Compounds.png]

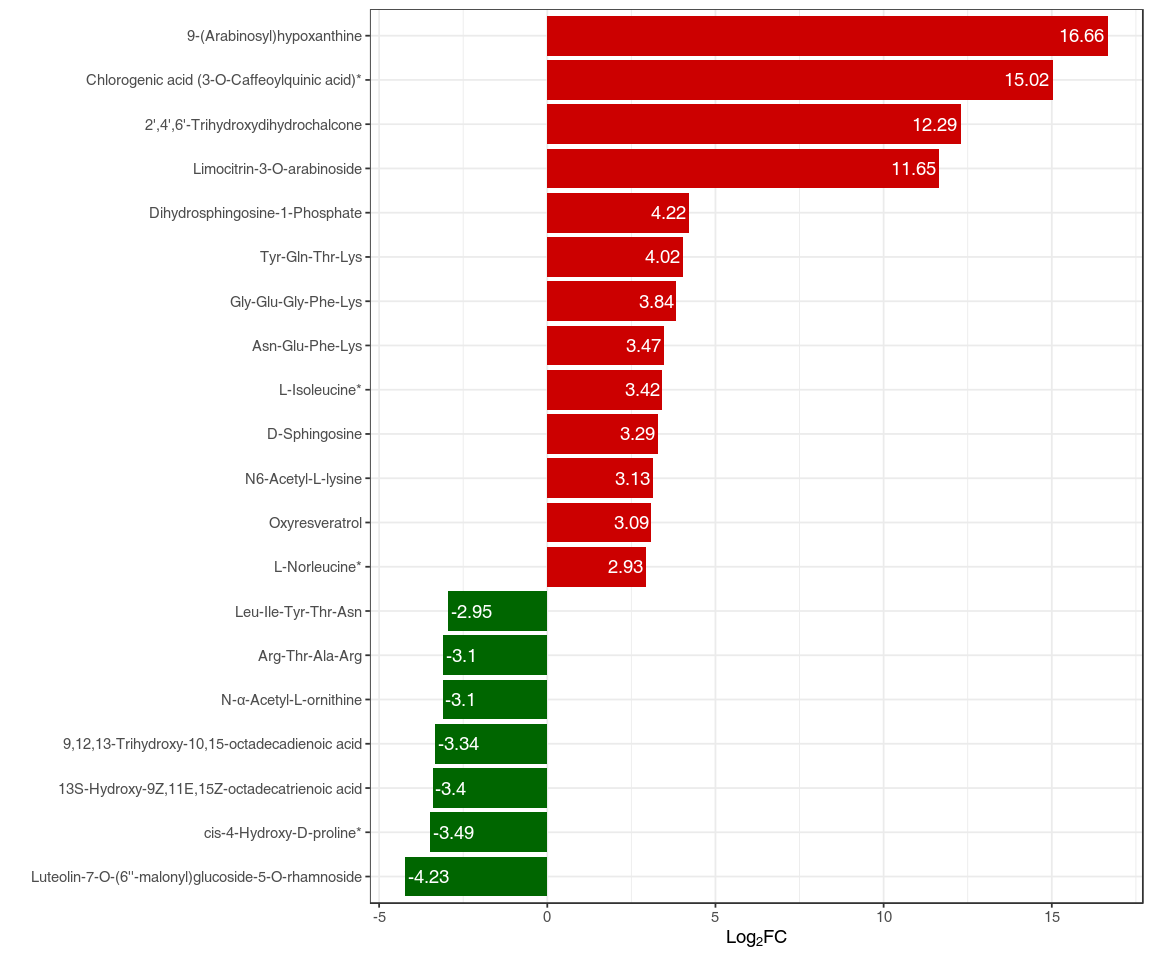

Supplement: Supplementary file 1 [file DataSheet1.zip › Supplementary Figure and Table/Supplementary Figure S5/M.indica/JC-M_vs_JW8-M_TopFcBarChart_Compounds.png]

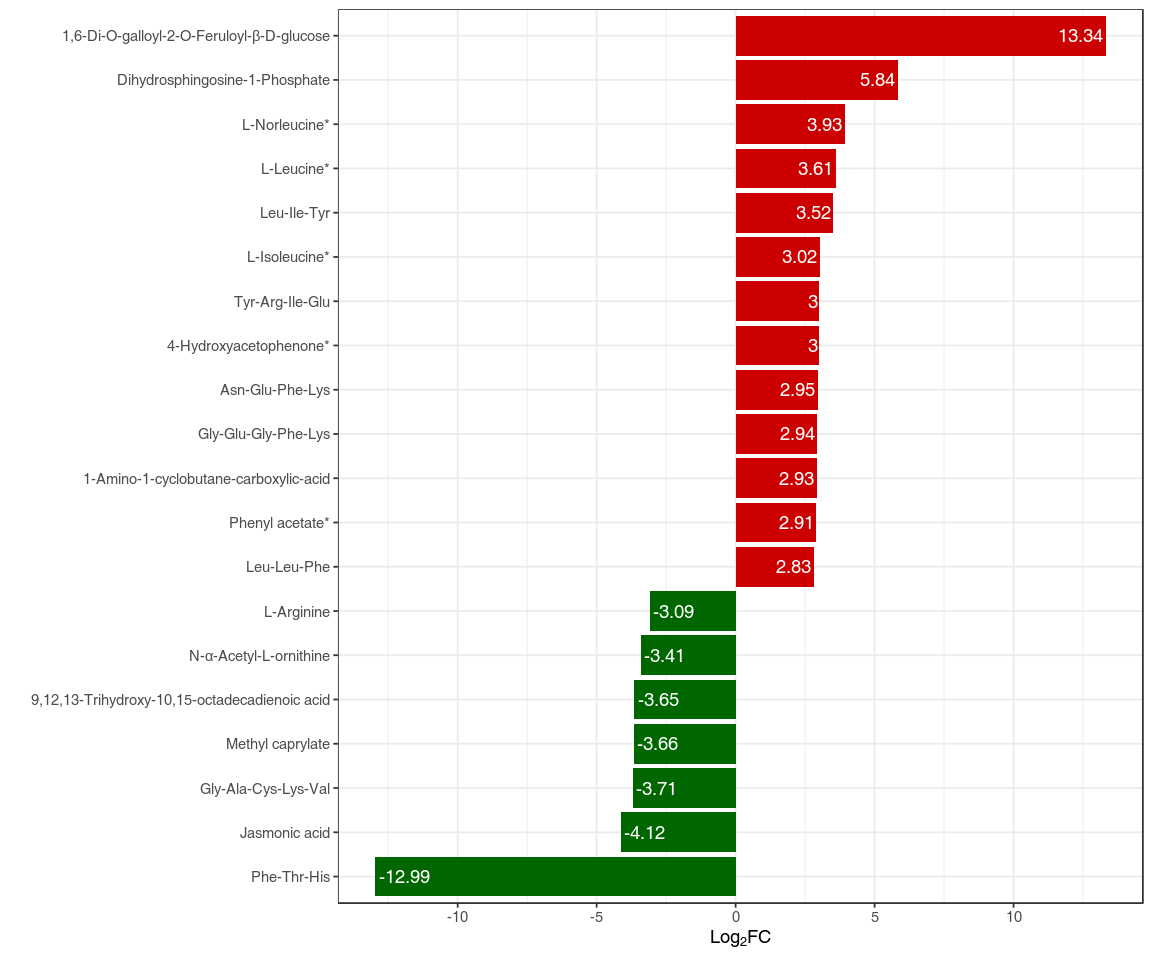

Supplement: Supplementary file 1 [file DataSheet1.zip › Supplementary Figure and Table/Supplementary Figure S5/Tainong/TC-M_vs_TW4-M_TopFcBarChart_Compounds.png]

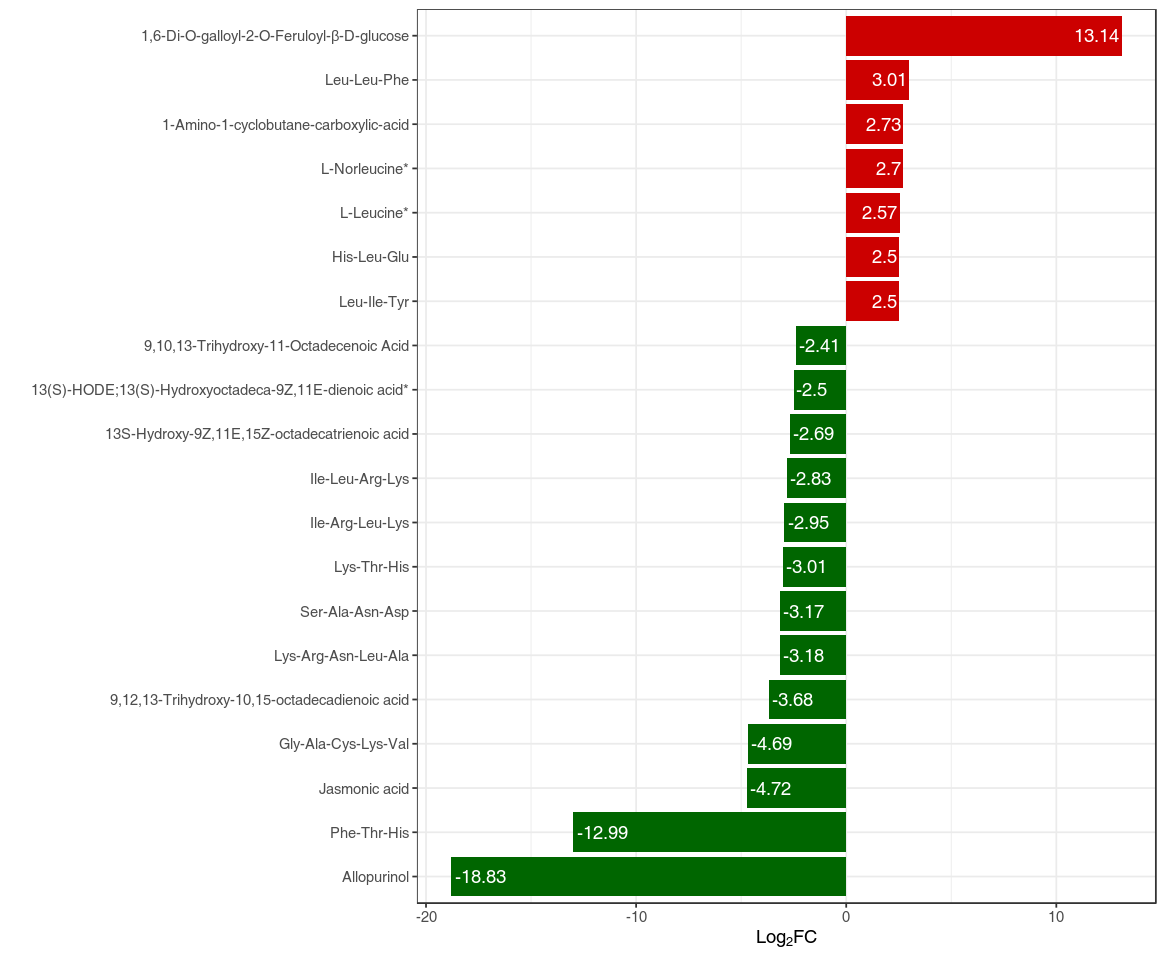

Supplement: Supplementary file 1 [file DataSheet1.zip › Supplementary Figure and Table/Supplementary Figure S5/Tainong/TC-M_vs_TW8-M_TopFcBarChart_Compounds.png]

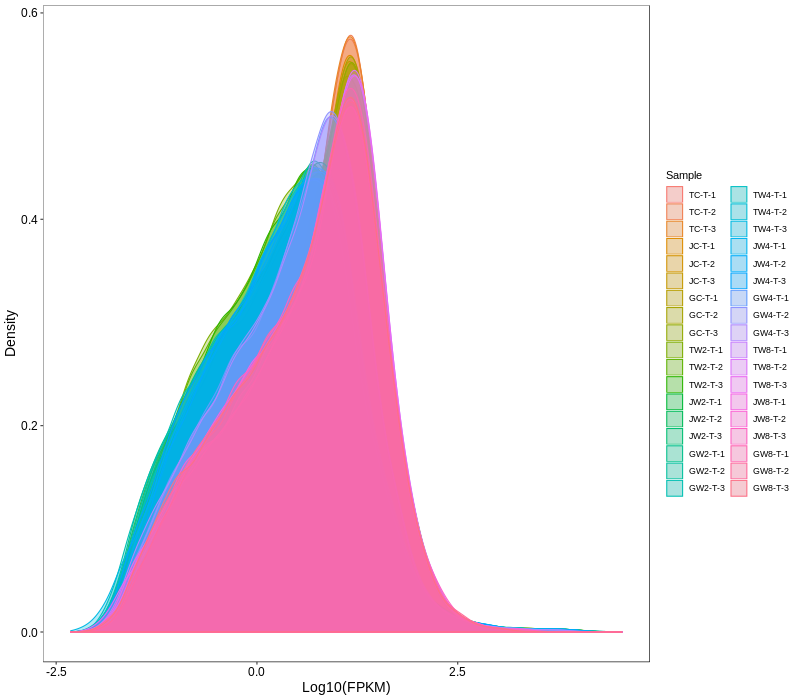

Supplement: Supplementary file 1 [file DataSheet1.zip › Supplementary Figure and Table/Supplementary Figure S6.png]

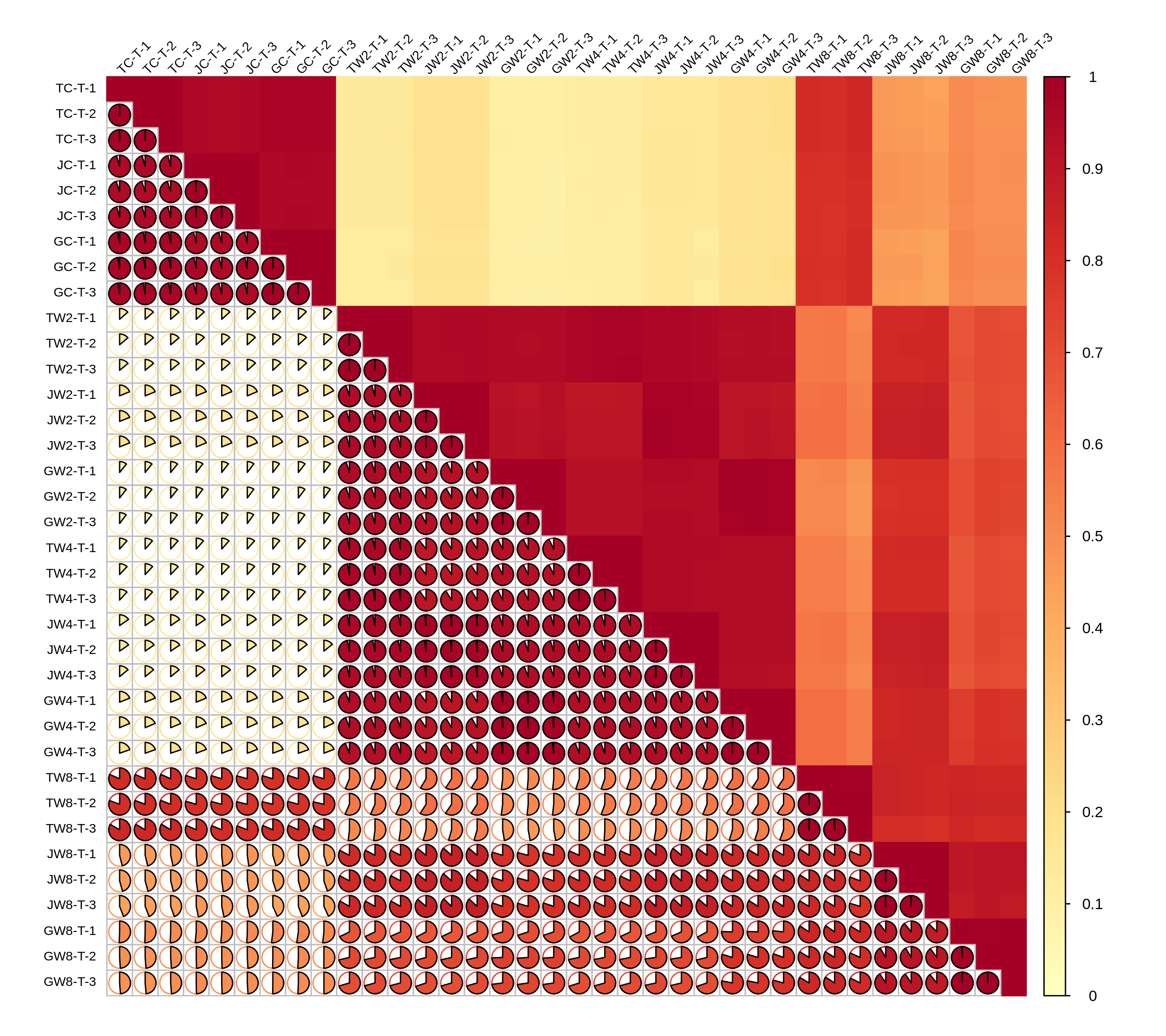

Supplement: Supplementary file 1 [file DataSheet1.zip › Supplementary Figure and Table/Supplementary Figure S7.png]

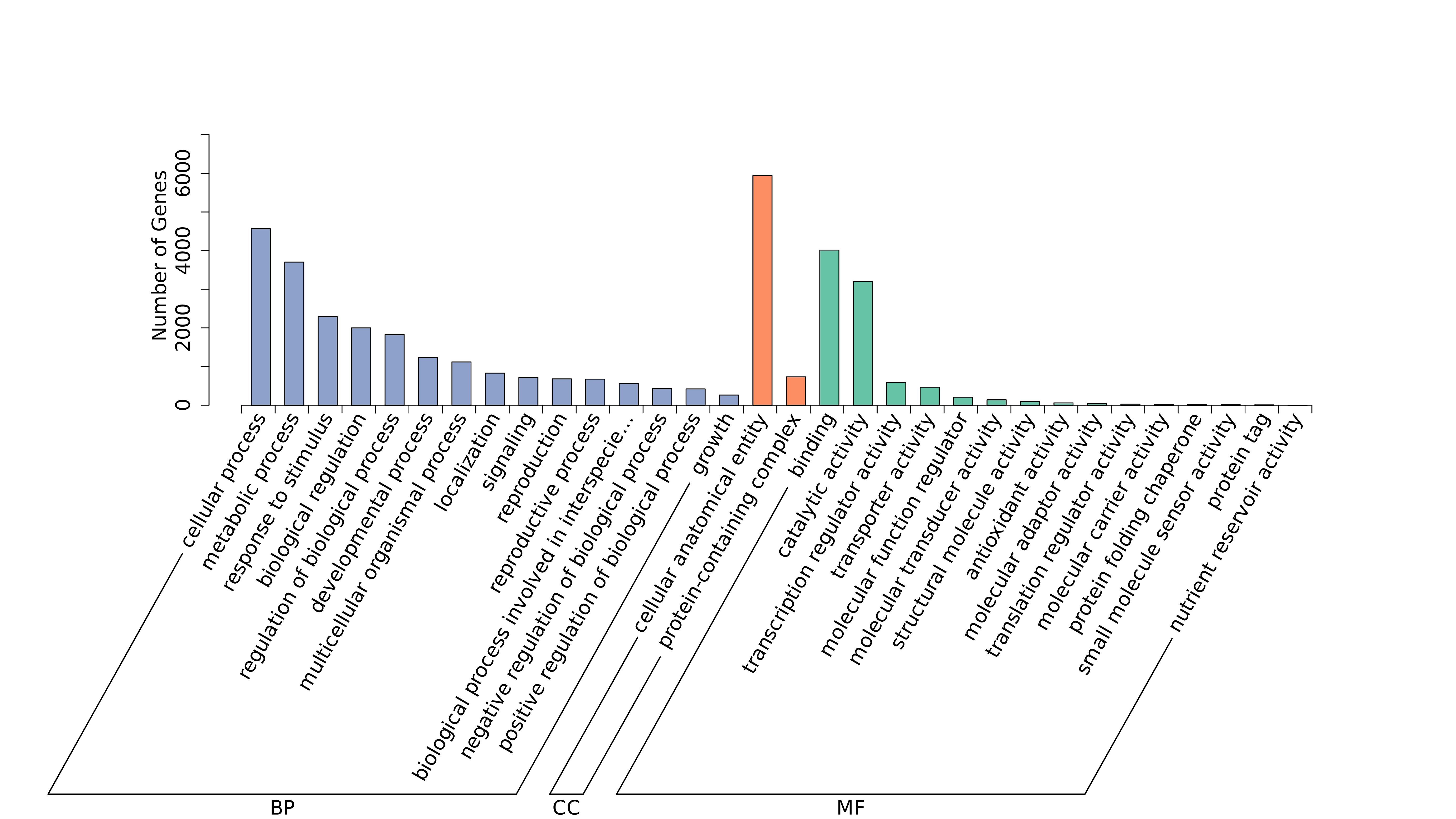

Supplement: Supplementary file 1 [file DataSheet1.zip › Supplementary Figure and Table/Supplementary Figure S9/Guiqi/GC-T_vs_GW4-T.GO.classification.png]

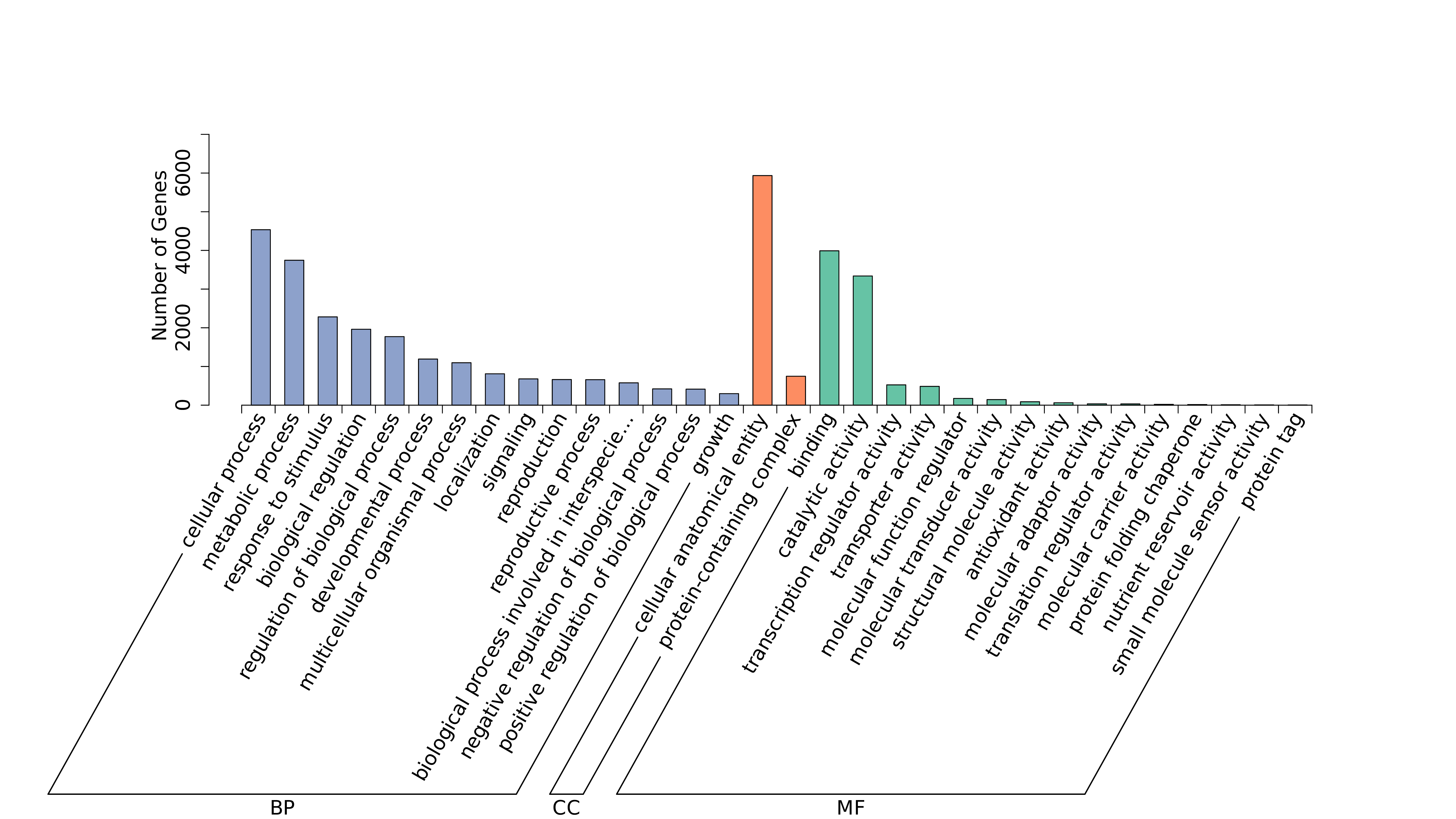

Supplement: Supplementary file 1 [file DataSheet1.zip › Supplementary Figure and Table/Supplementary Figure S9/Guiqi/GC-T_vs_GW8-T.GO.classification.png]

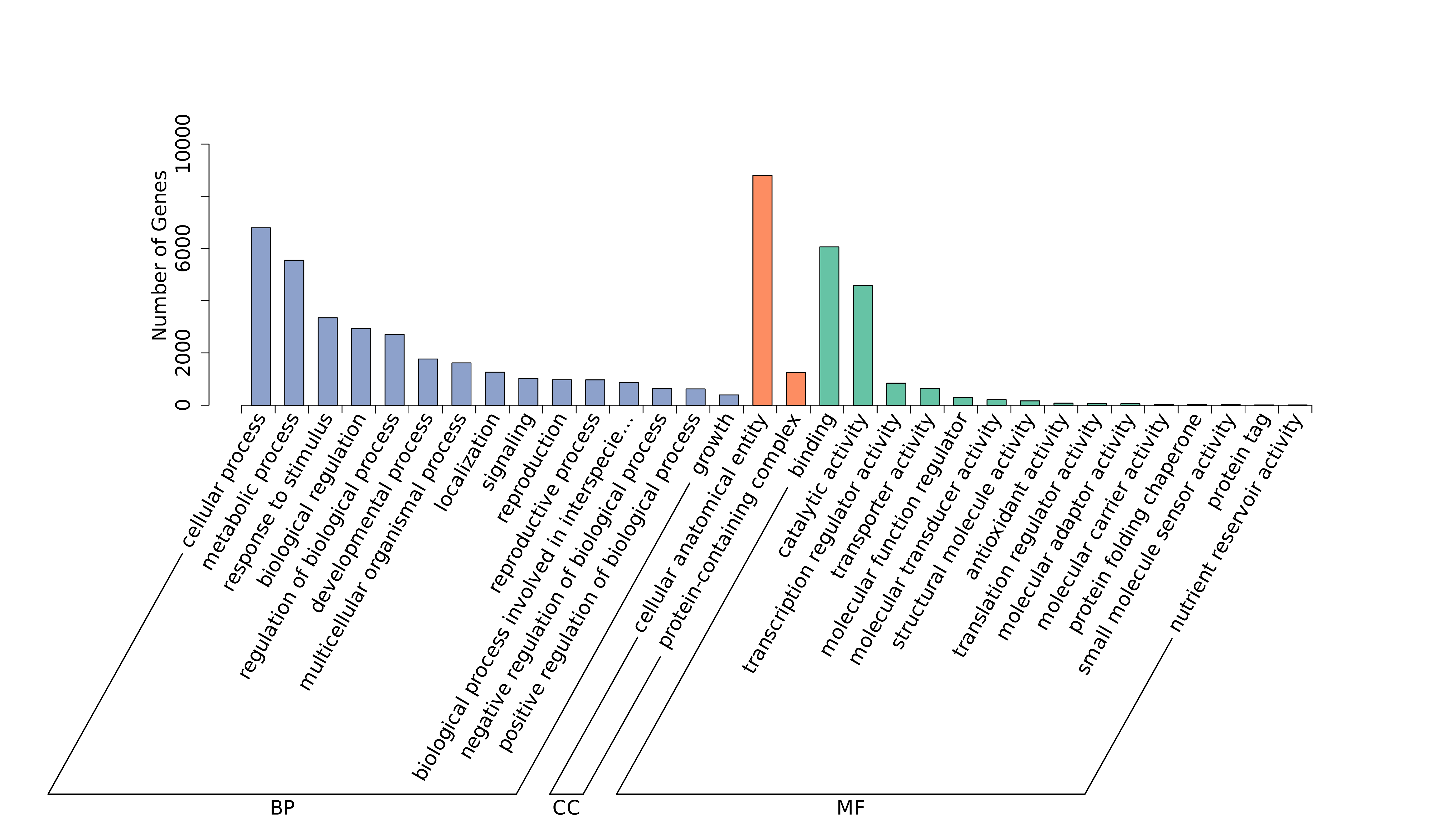

Supplement: Supplementary file 1 [file DataSheet1.zip › Supplementary Figure and Table/Supplementary Figure S9/M.indica/JC-T_vs_JW4-T.GO.classification.png]

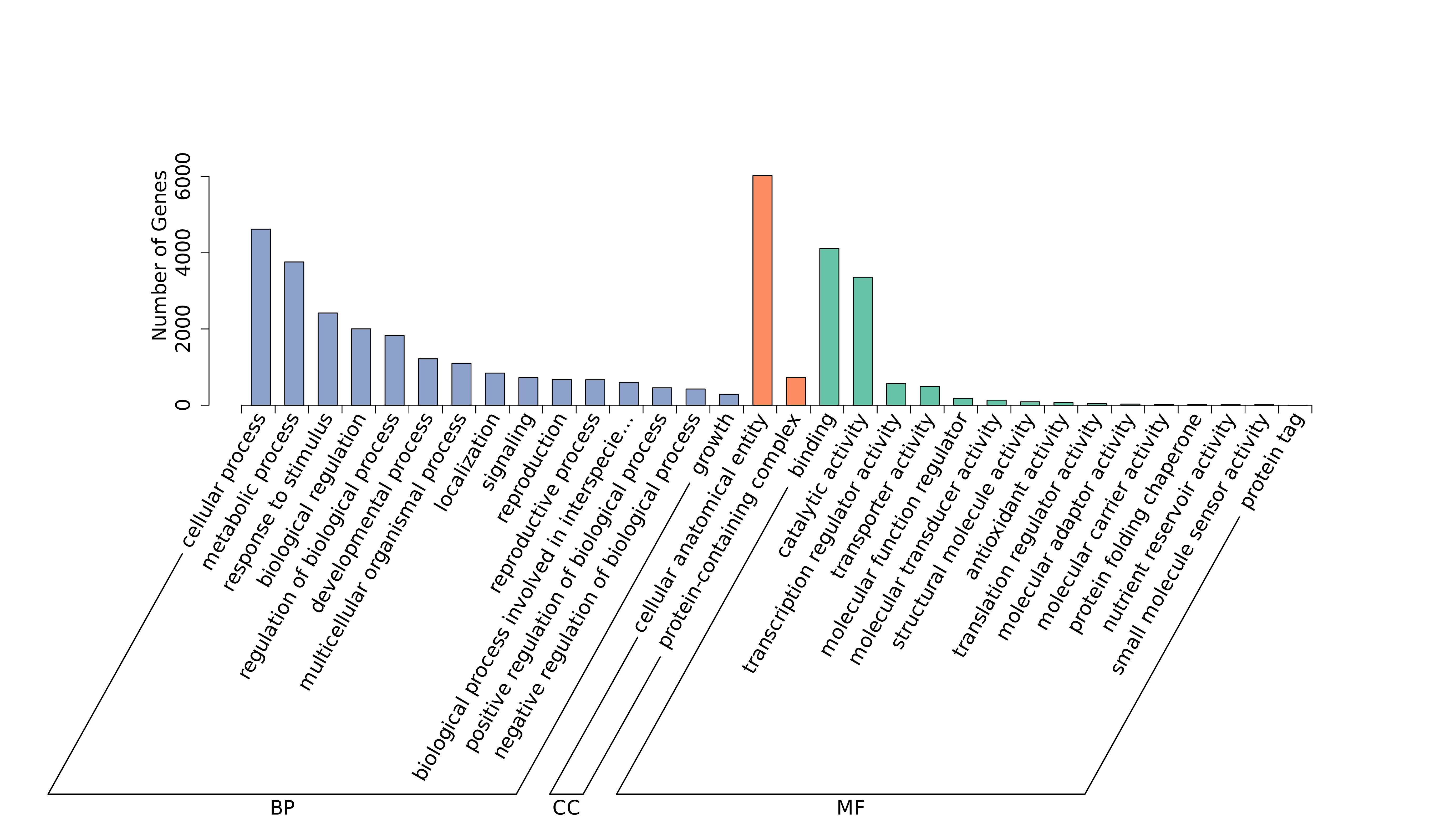

Supplement: Supplementary file 1 [file DataSheet1.zip › Supplementary Figure and Table/Supplementary Figure S9/M.indica/JC-T_vs_JW8-T.GO.classification.png]

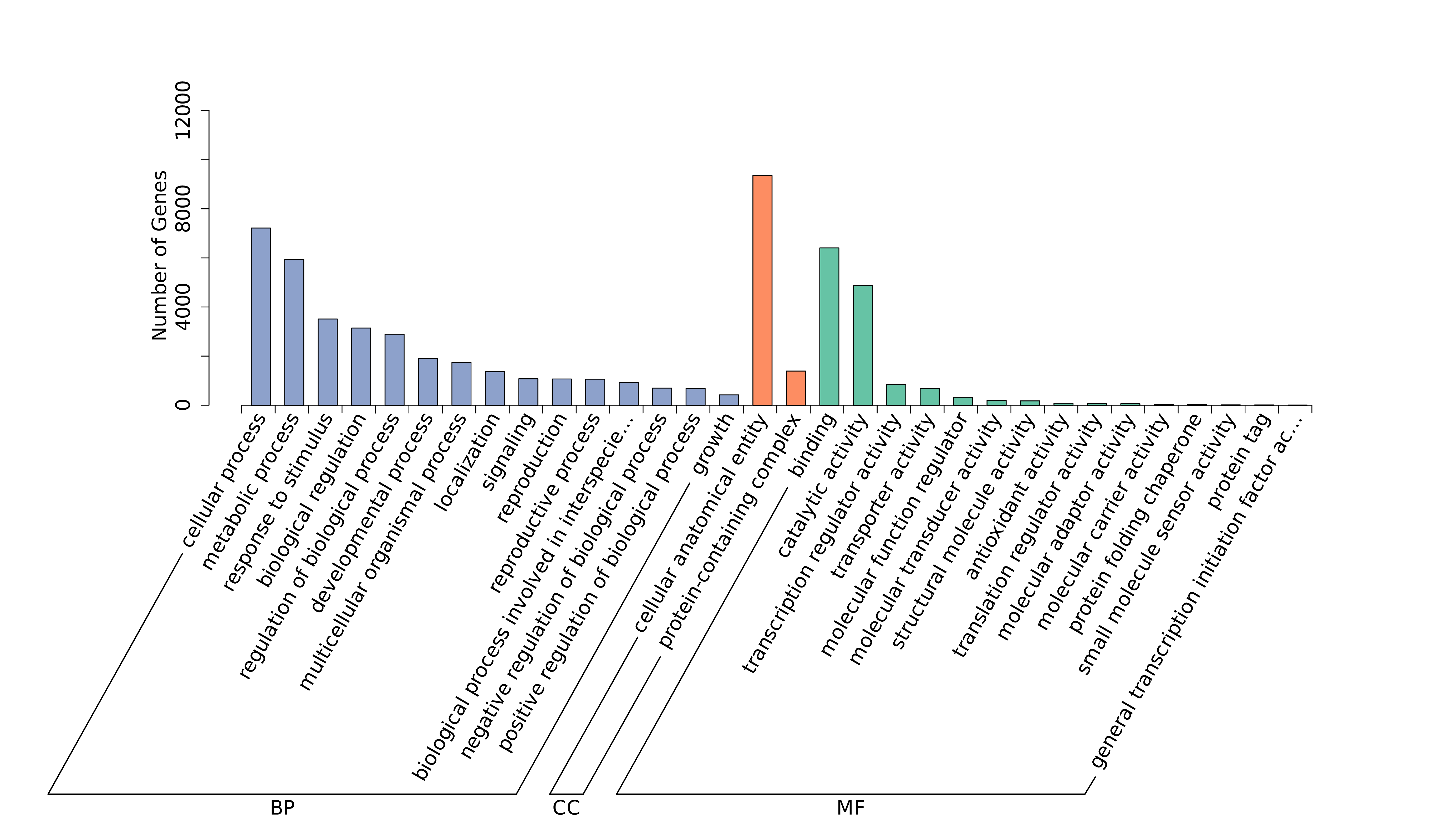

Supplement: Supplementary file 1 [file DataSheet1.zip › Supplementary Figure and Table/Supplementary Figure S9/Tainong/TC-T_vs_TW4-T.GO.classification.png]

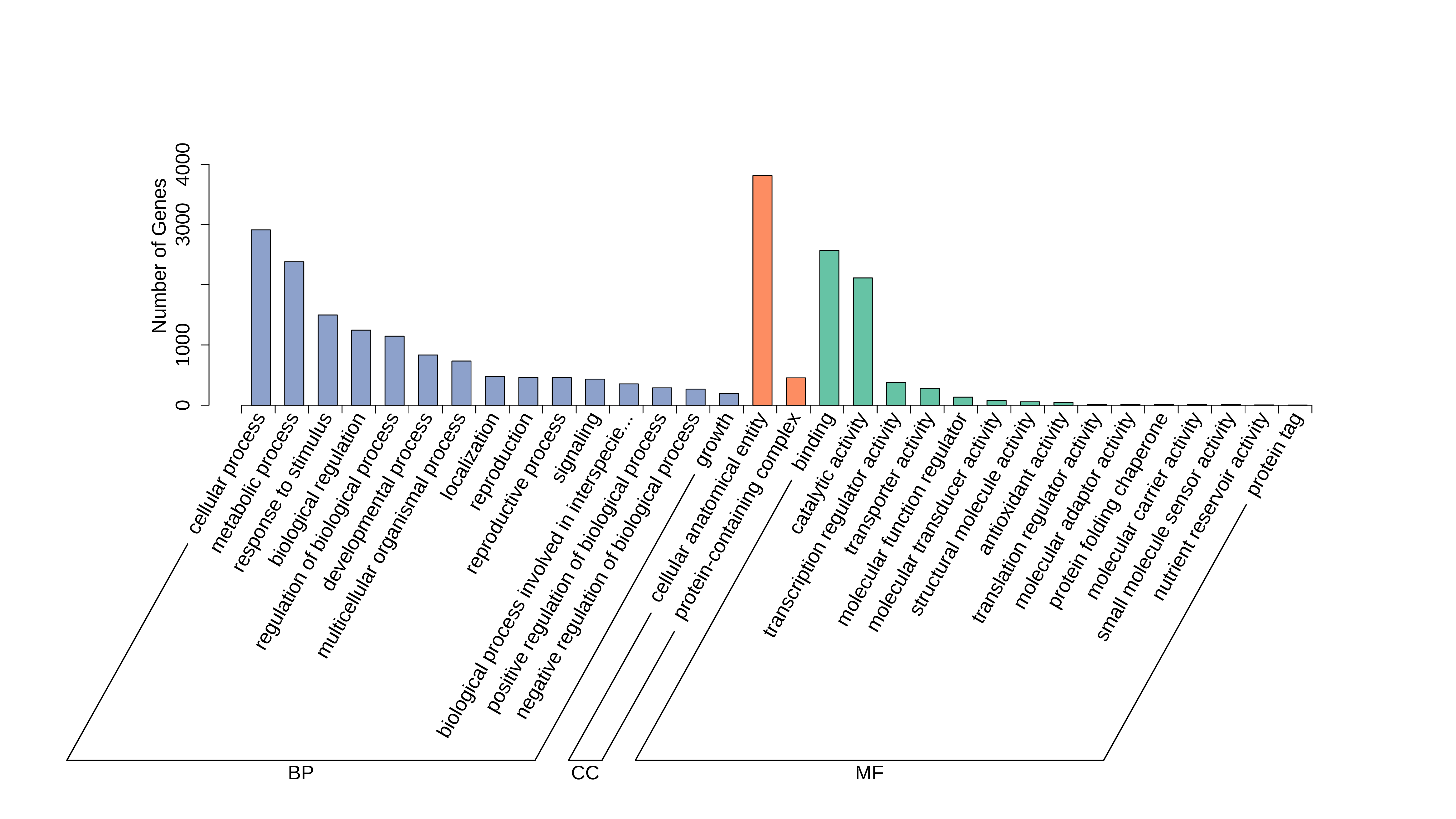

Supplement: Supplementary file 1 [file DataSheet1.zip › Supplementary Figure and Table/Supplementary Figure S9/Tainong/TC-T_vs_TW8-T.GO.classification.png]

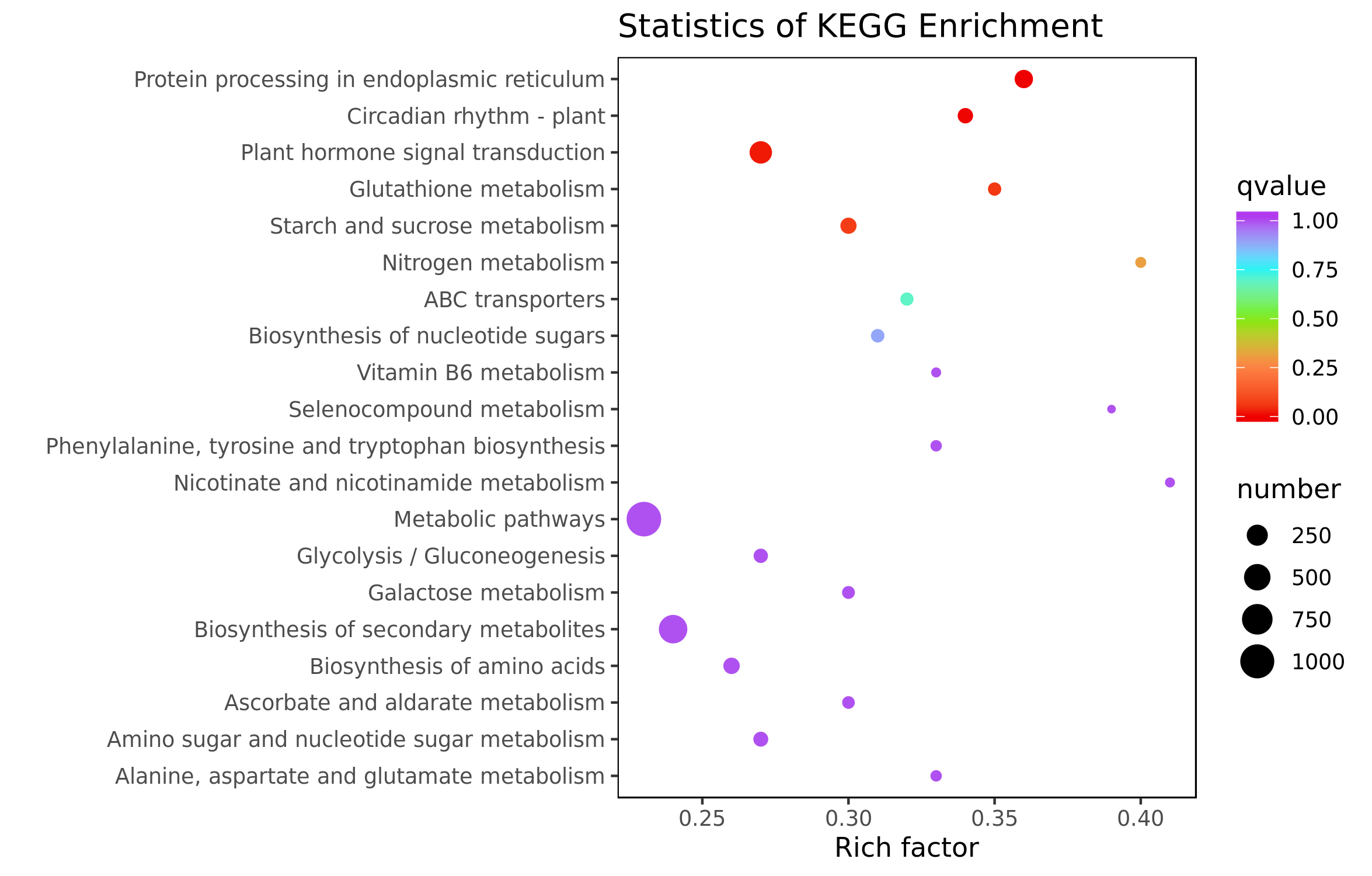

Supplement: Supplementary file 1 [file DataSheet1.zip › Supplementary Figure and Table/Supplementary Figure S10/Guiqi/GC-T_vs_GW4-T.pathwayenrichment.png]

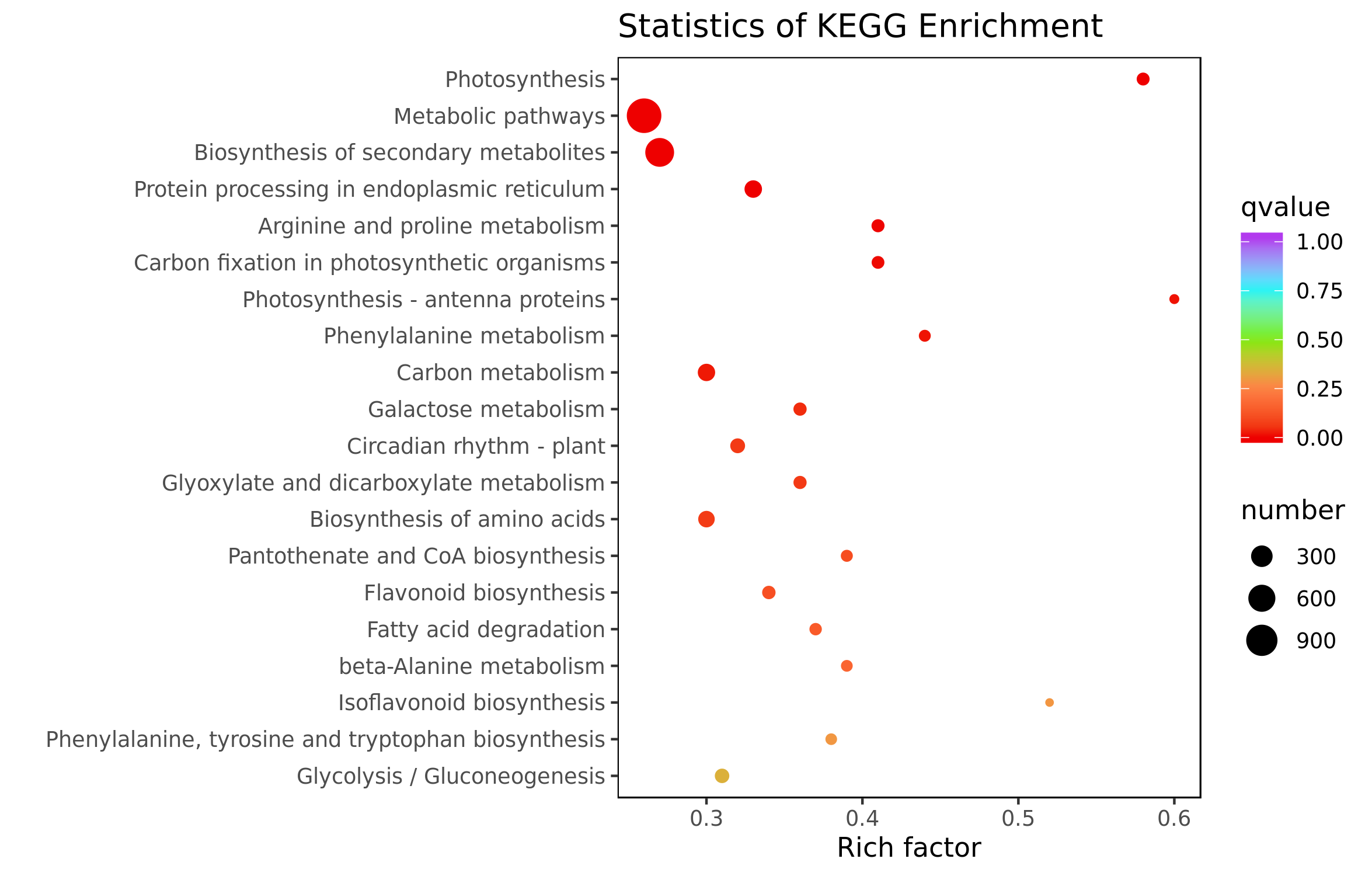

Supplement: Supplementary file 1 [file DataSheet1.zip › Supplementary Figure and Table/Supplementary Figure S10/Guiqi/GC-T_vs_GW8-T.pathwayenrichment.png]

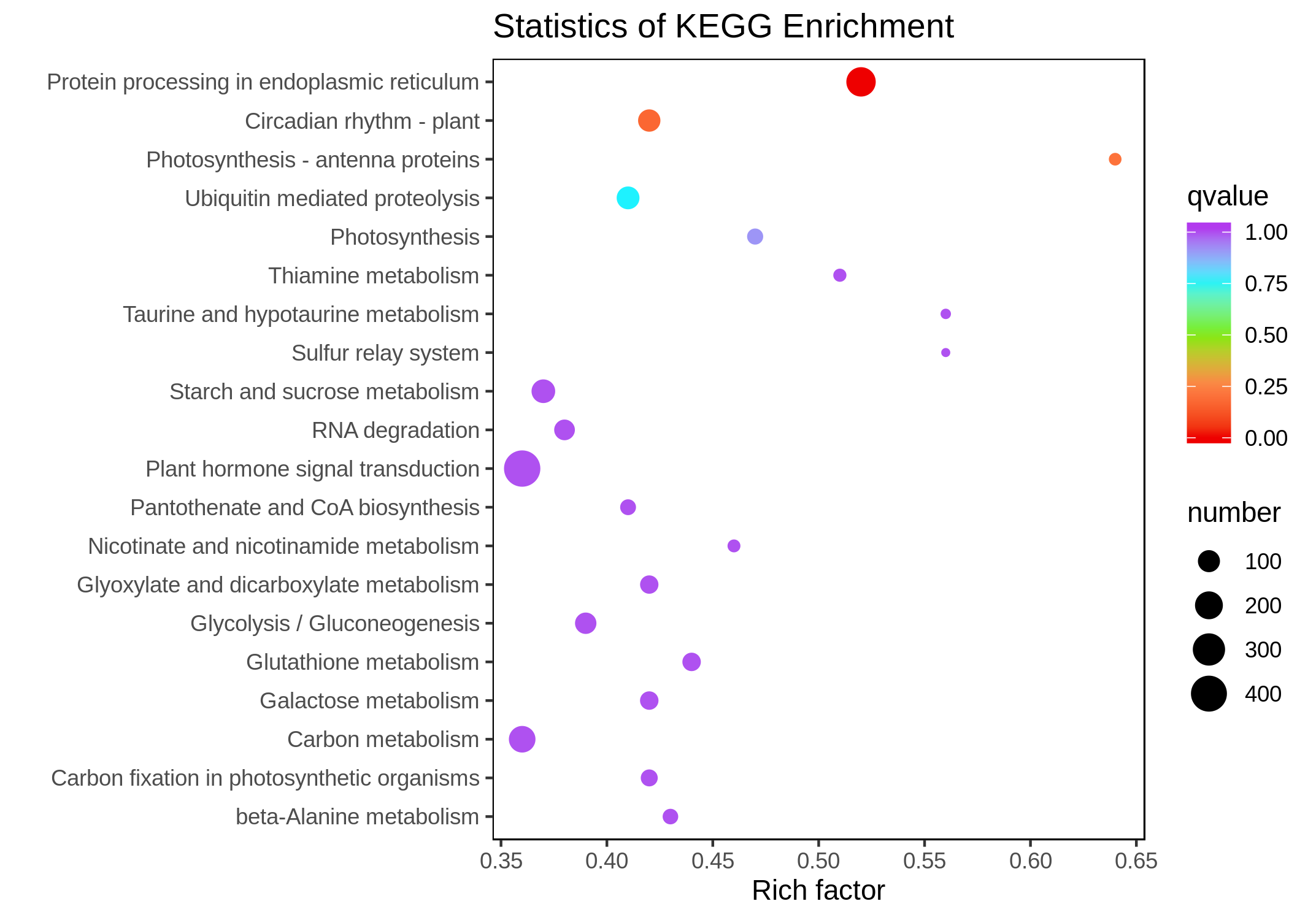

Supplement: Supplementary file 1 [file DataSheet1.zip › Supplementary Figure and Table/Supplementary Figure S10/M.indica/JC-T_vs_JW4-T.pathwayenrichment.png]

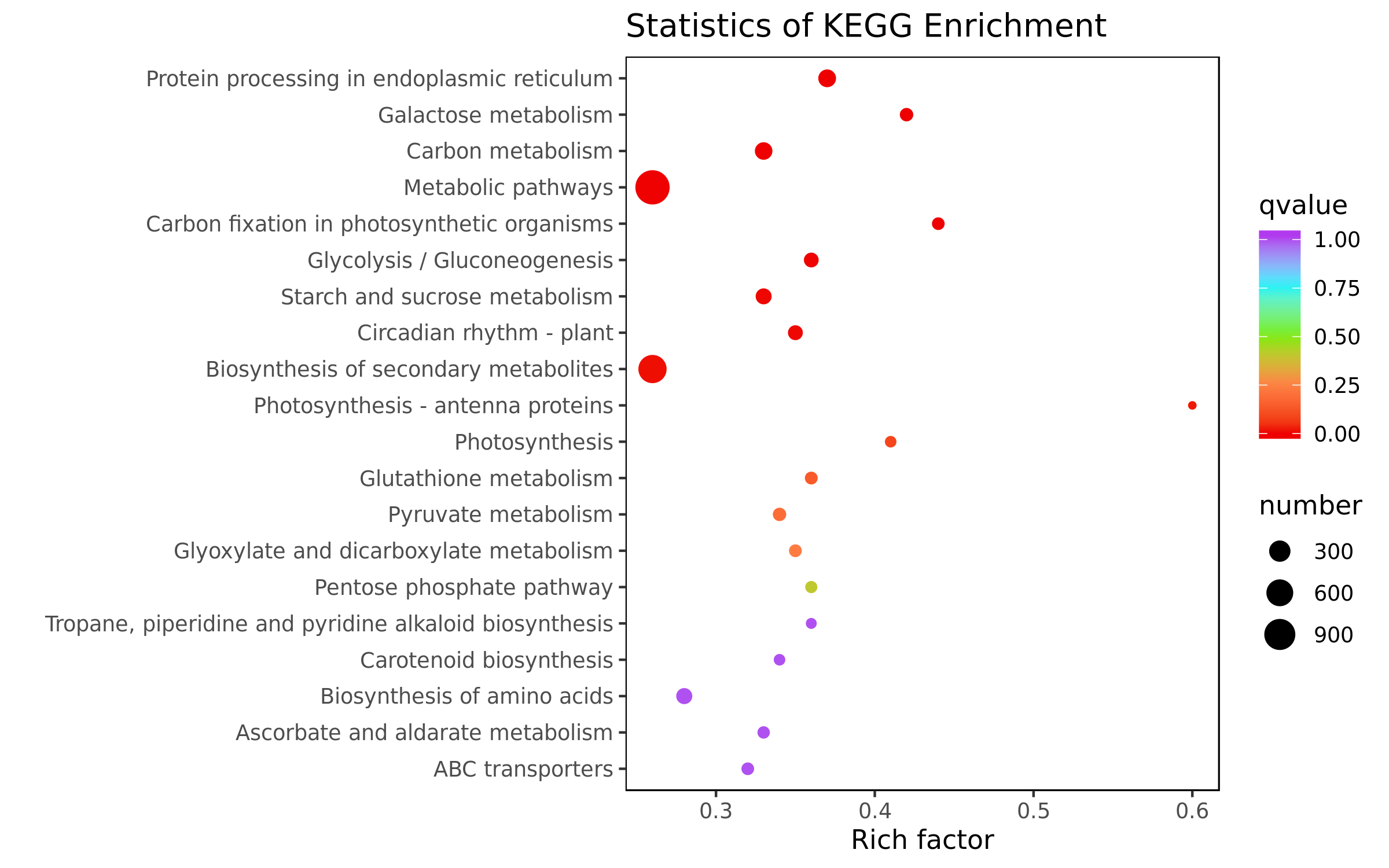

Supplement: Supplementary file 1 [file DataSheet1.zip › Supplementary Figure and Table/Supplementary Figure S10/M.indica/JC-T_vs_JW8-T.pathwayenrichment.png]

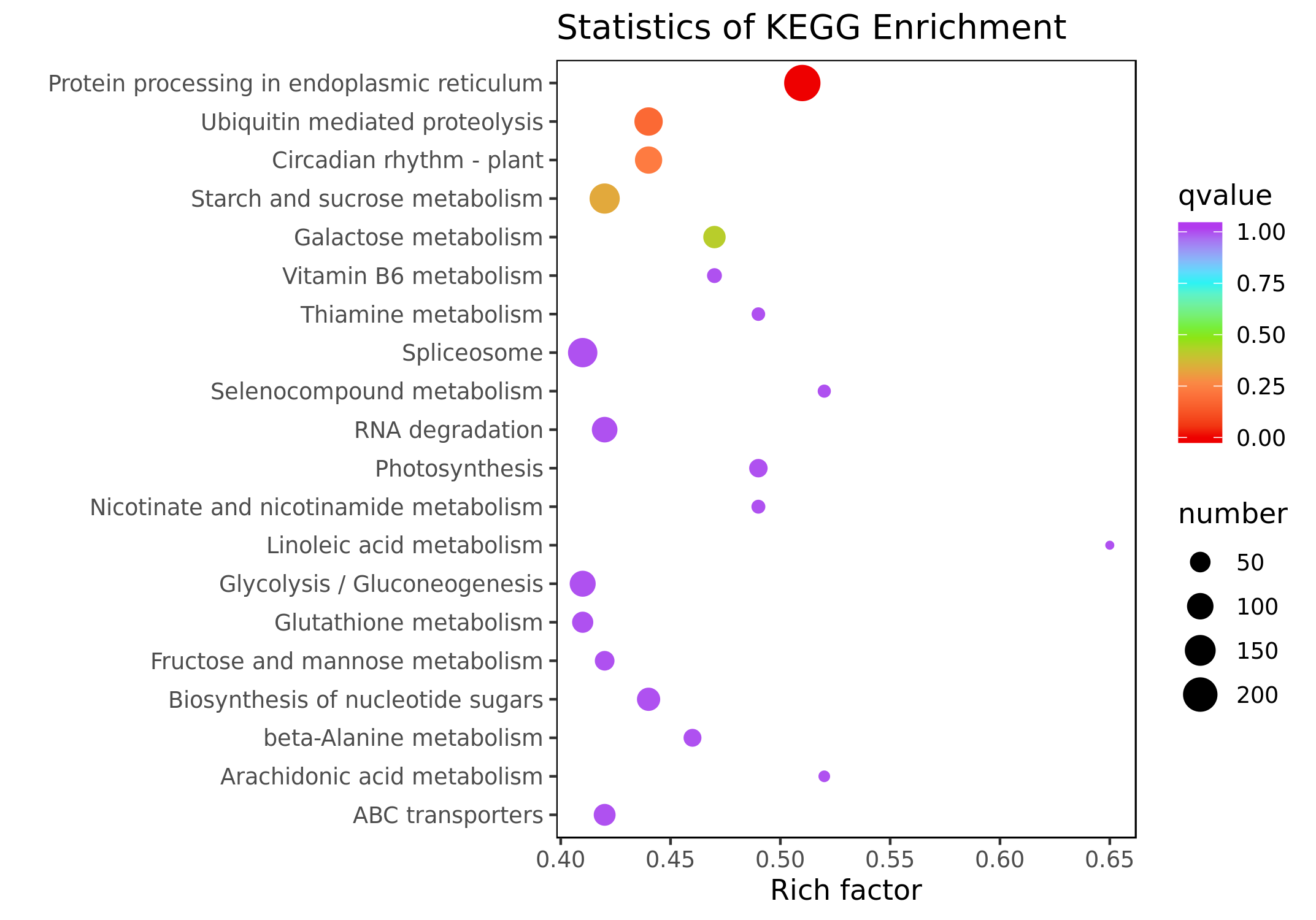

Supplement: Supplementary file 1 [file DataSheet1.zip › Supplementary Figure and Table/Supplementary Figure S10/Tainong/TC-T_vs_TW4-T.pathwayenrichment.png]

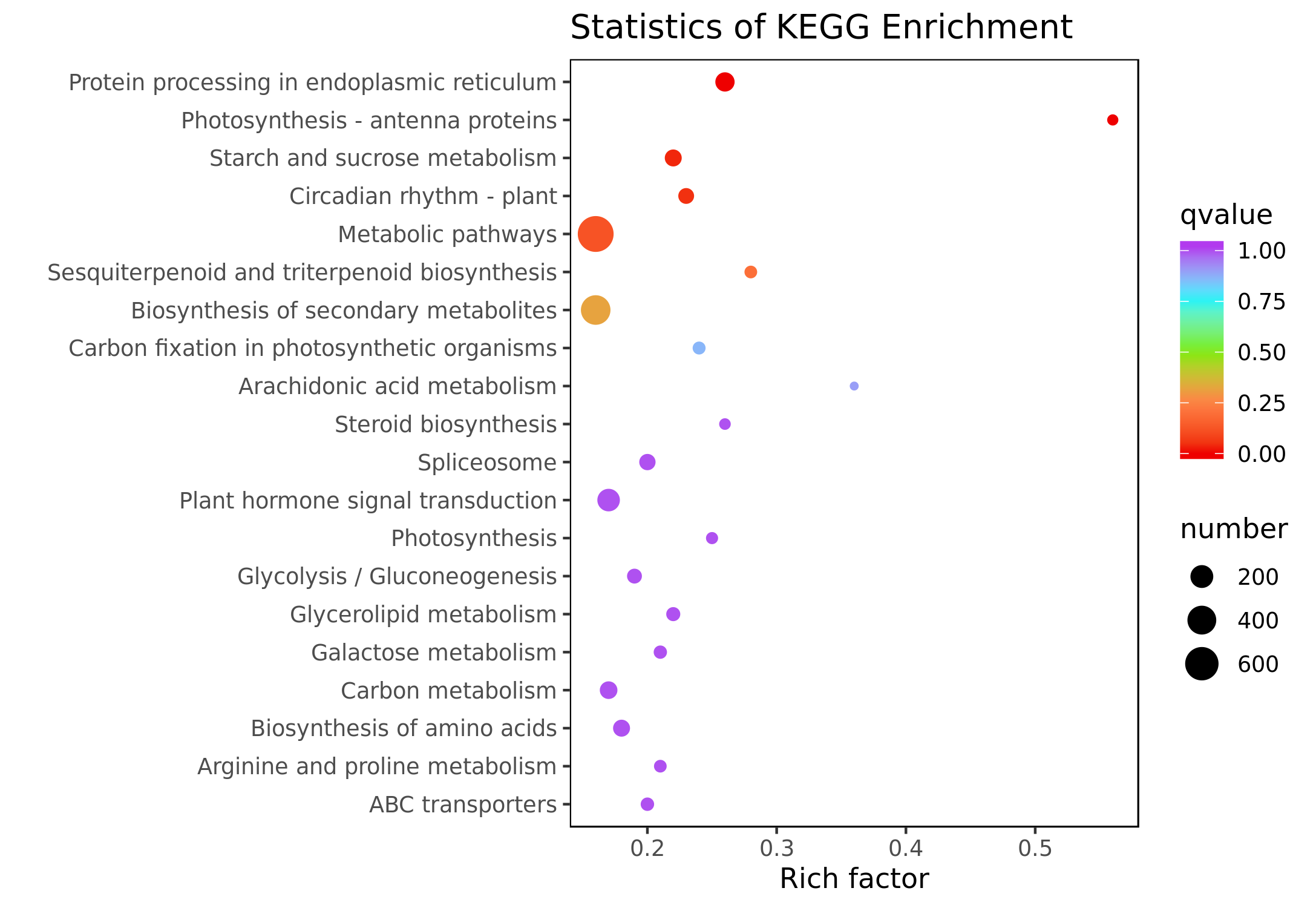

Supplement: Supplementary file 1 [file DataSheet1.zip › Supplementary Figure and Table/Supplementary Figure S10/Tainong/TC-T_vs_TW8-T.pathwayenrichment.png]

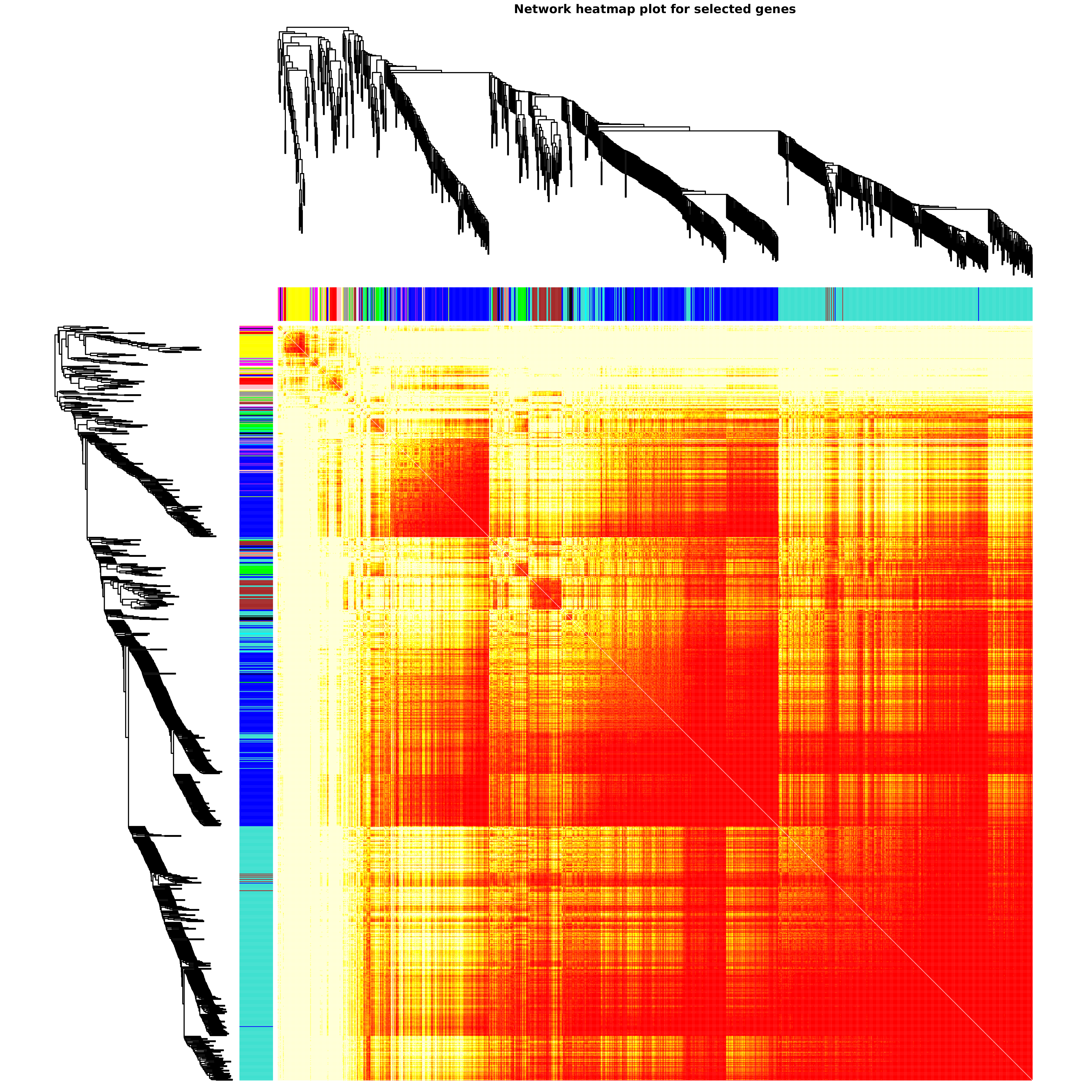

Supplement: Supplementary file 1 [file DataSheet1.zip › Supplementary Figure and Table/Supplementary Figure S11.png]

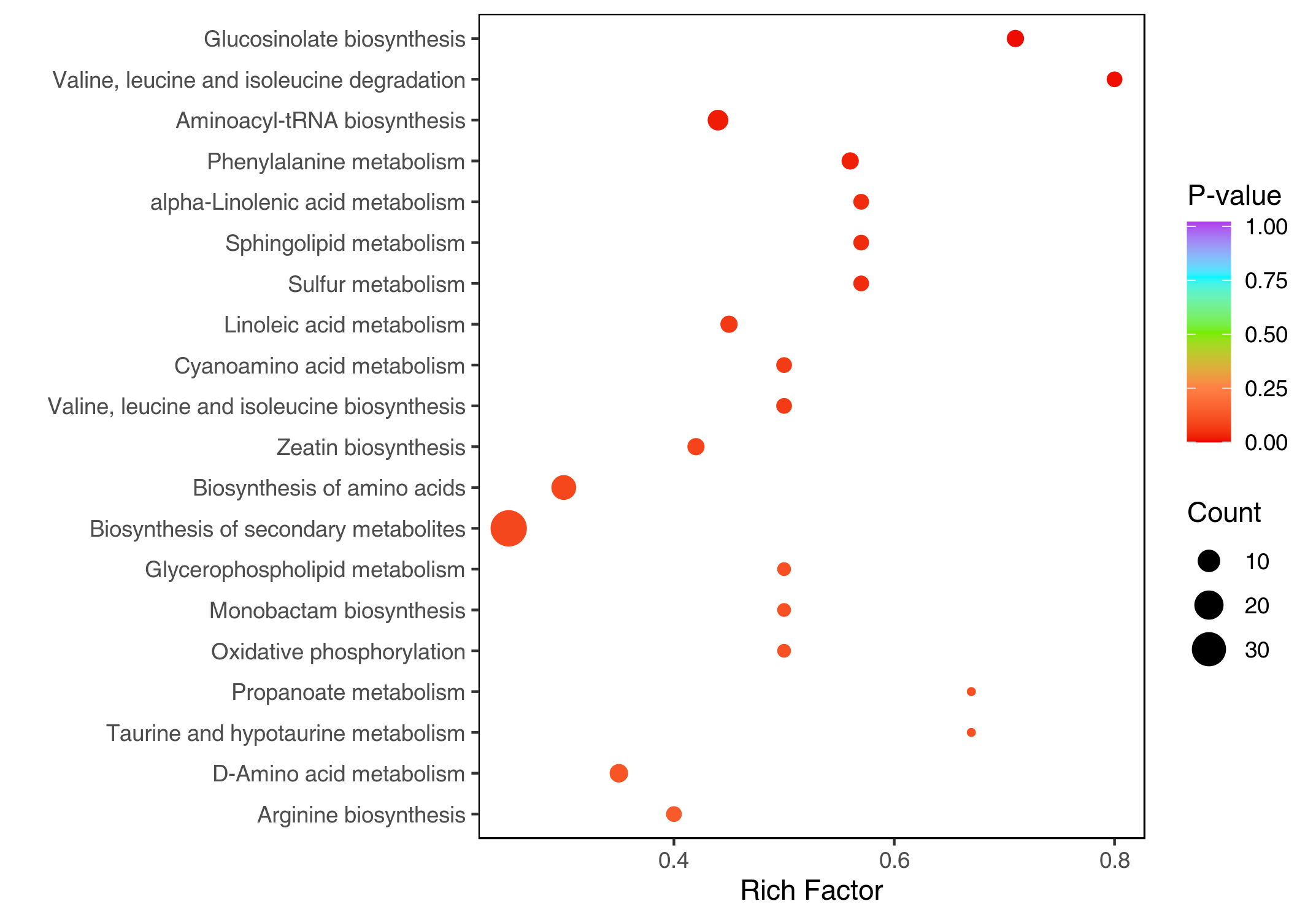

Supplement: Supplementary file 1 [file DataSheet1.zip › Supplementary Figure and Table/Supplementary Figure S4/Tainong/TC-M_vs_TW2-M_KEGG_Enrichment.png]

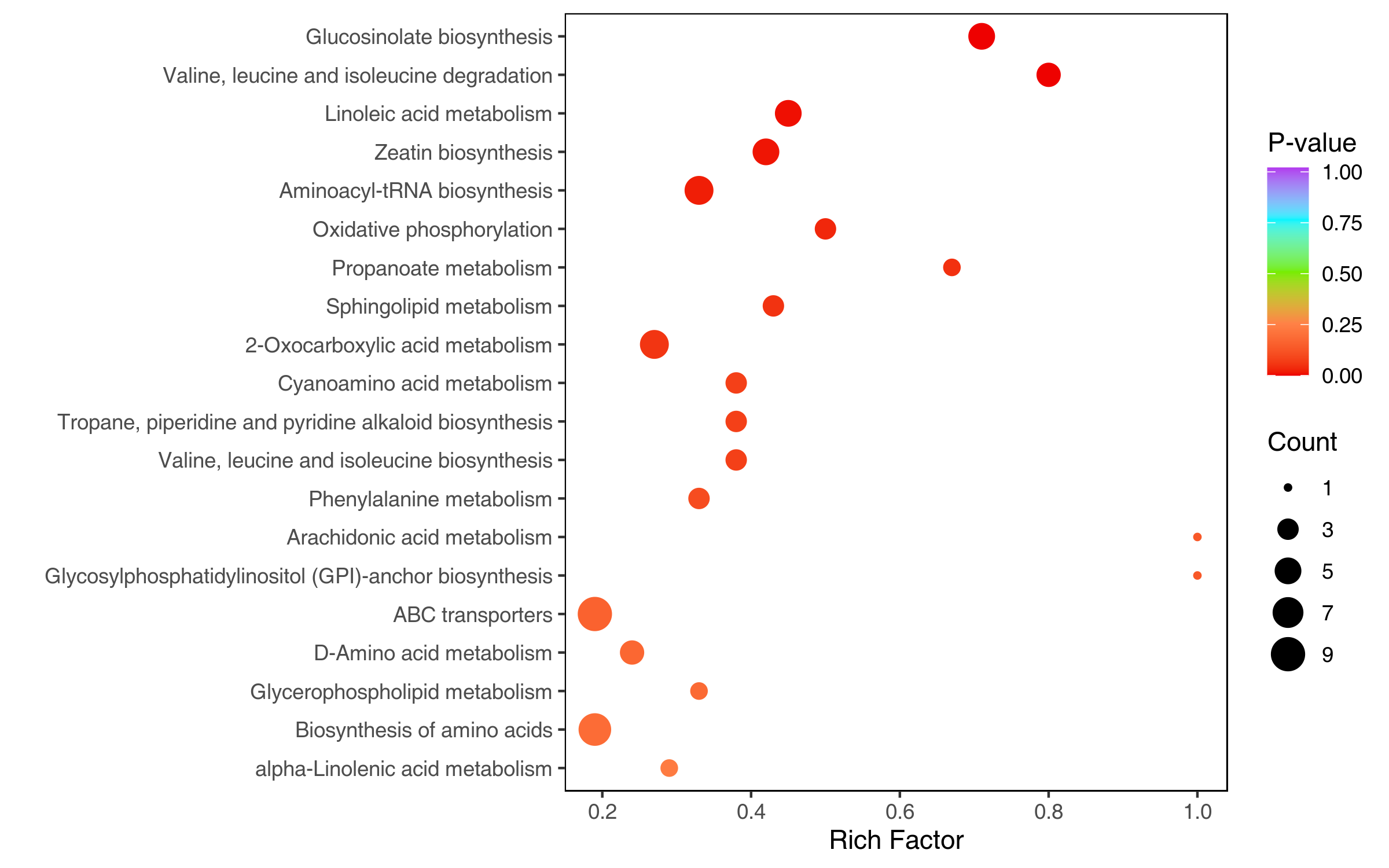

Supplement: Supplementary file 1 [file DataSheet1.zip › Supplementary Figure and Table/Supplementary Figure S4/M.indica/JC-M_vs_JW2-M_KEGG_Enrichment.png]

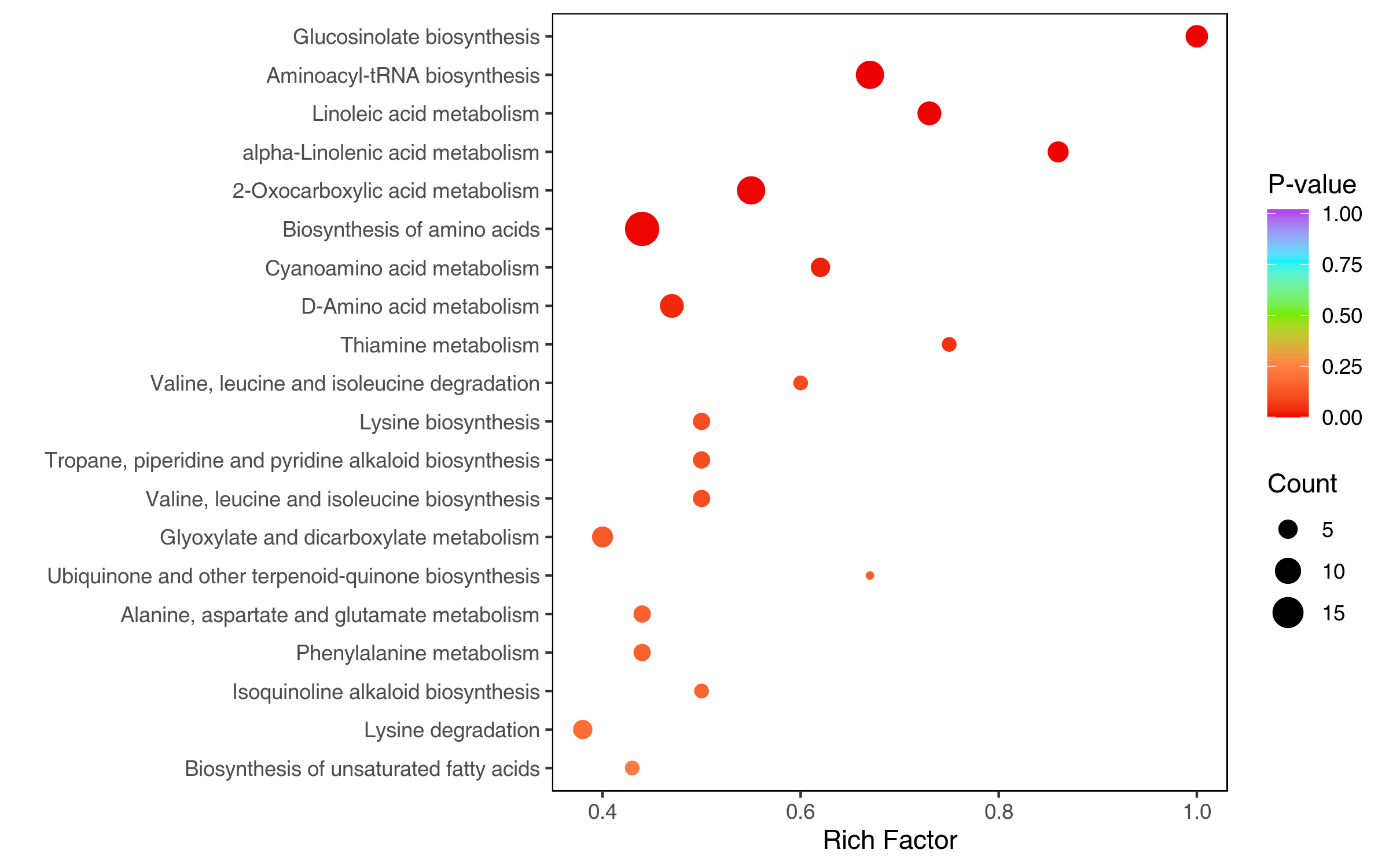

Supplement: Supplementary file 1 [file DataSheet1.zip › Supplementary Figure and Table/Supplementary Figure S4/Guiqi/GC-M_vs_GW2-M_KEGG_Enrichment.png]

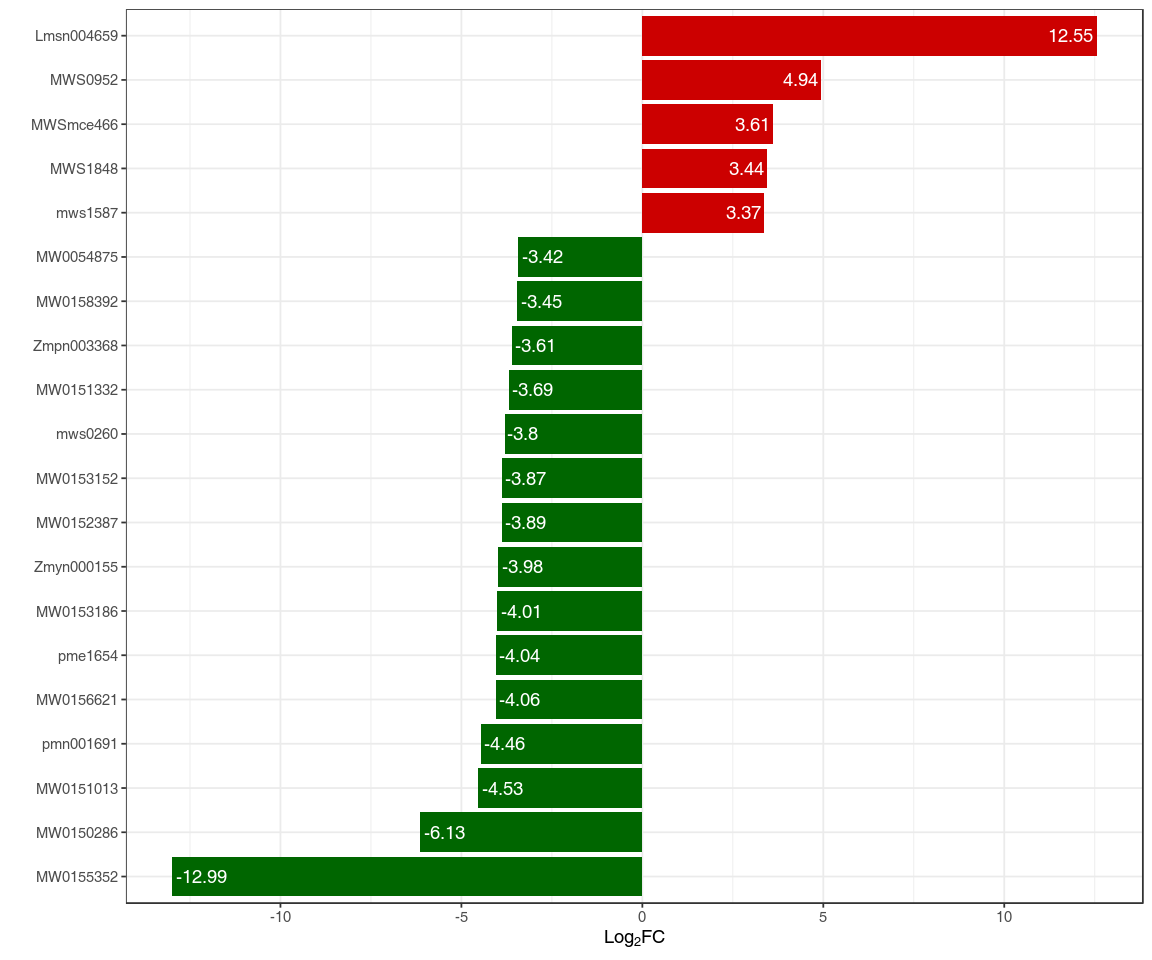

Supplement: Supplementary file 1 [file DataSheet1.zip › Supplementary Figure and Table/Supplementary Figure S5/Tainong/TC-M_vs_TW2-M_TopFcBarChart_Index.png]

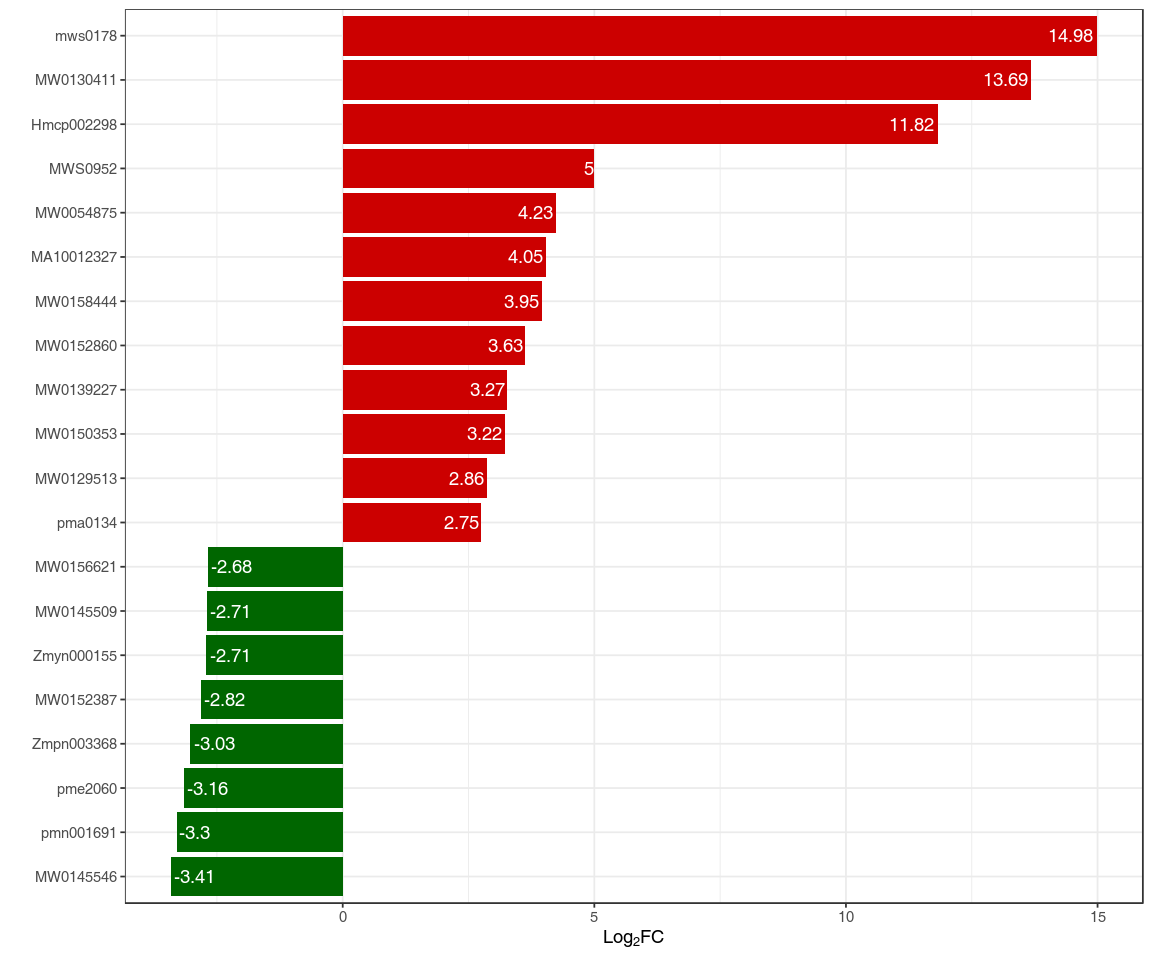

Supplement: Supplementary file 1 [file DataSheet1.zip › Supplementary Figure and Table/Supplementary Figure S5/M.indica/JC-M_vs_JW2-M_TopFcBarChart_Index.png]

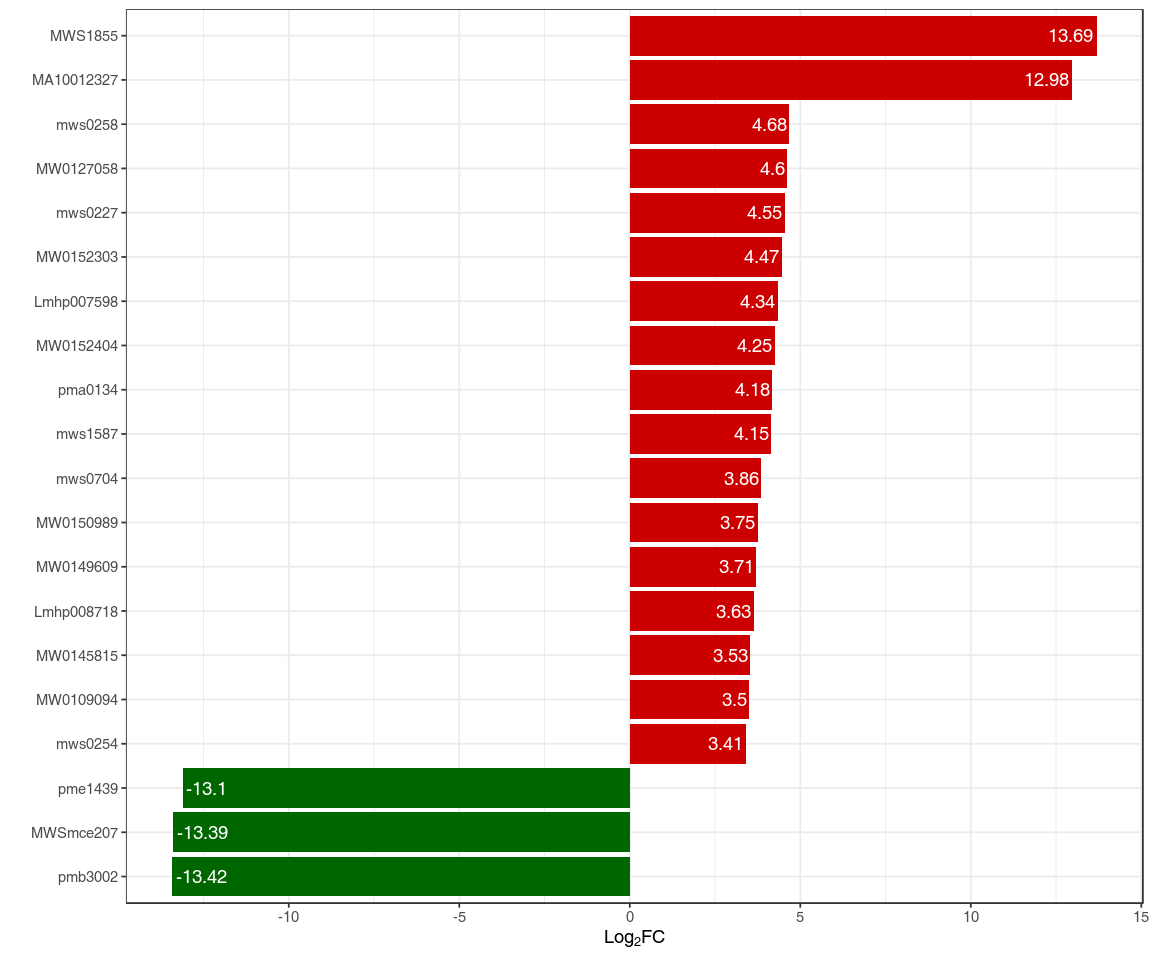

Supplement: Supplementary file 1 [file DataSheet1.zip › Supplementary Figure and Table/Supplementary Figure S5/Guiqi/GC-M_vs_GW2-M_TopFcBarChart_Index.png]

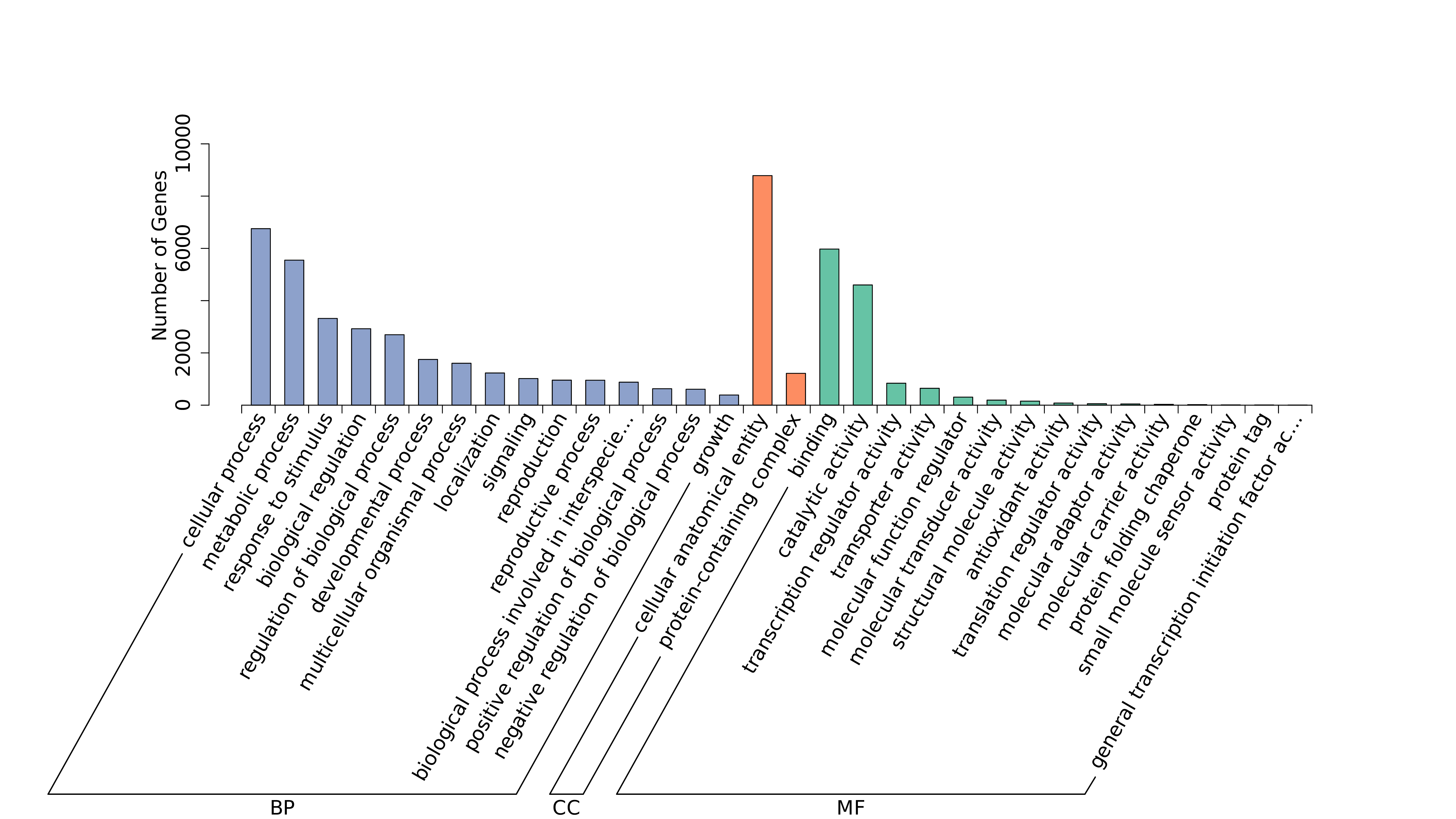

Supplement: Supplementary file 1 [file DataSheet1.zip › Supplementary Figure and Table/Supplementary Figure S9/Tainong/TC-T_vs_TW2-T.GO.classification.png]

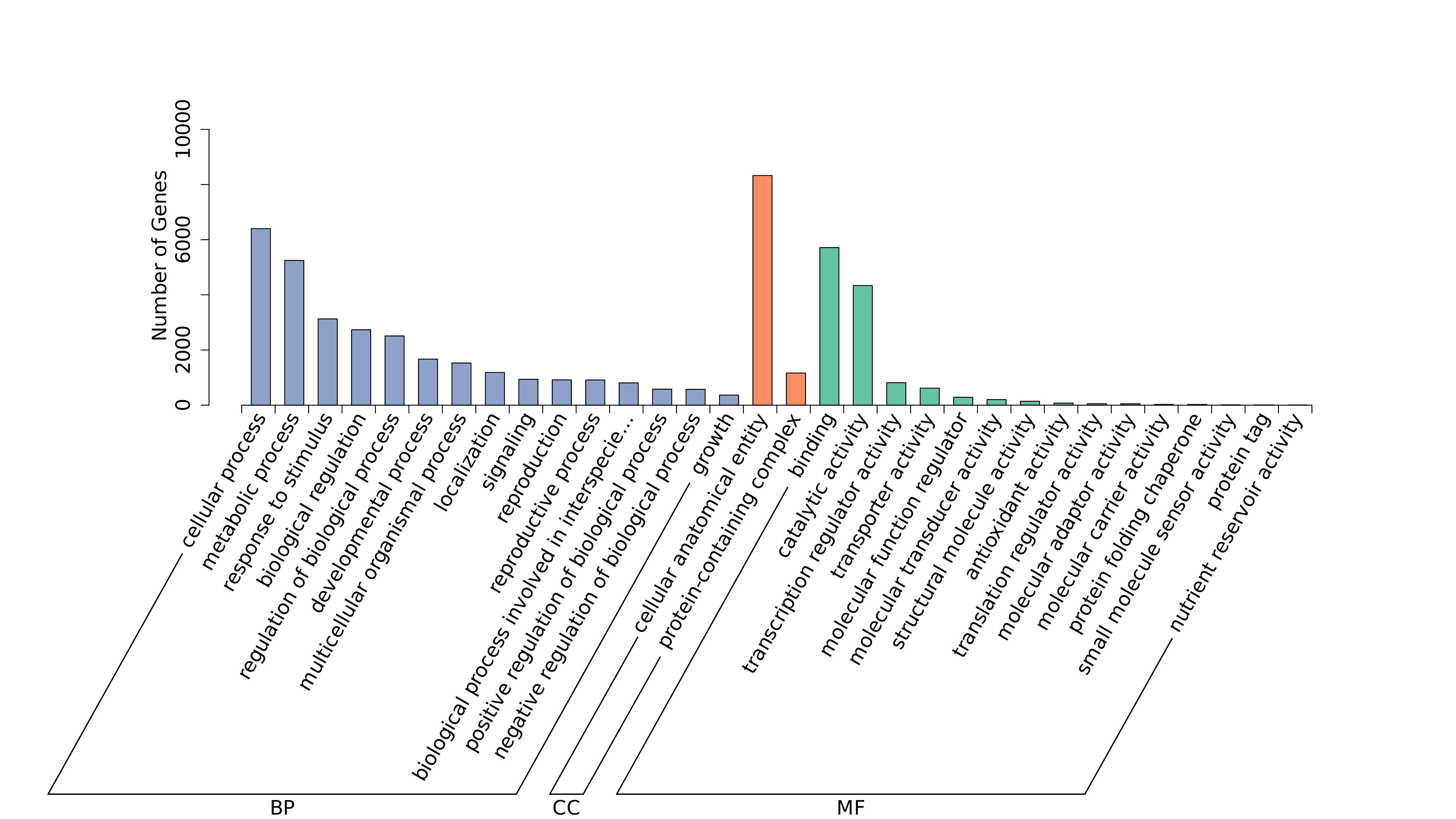

Supplement: Supplementary file 1 [file DataSheet1.zip › Supplementary Figure and Table/Supplementary Figure S9/M.indica/JC-T_vs_JW2-T.GO.classification.png]

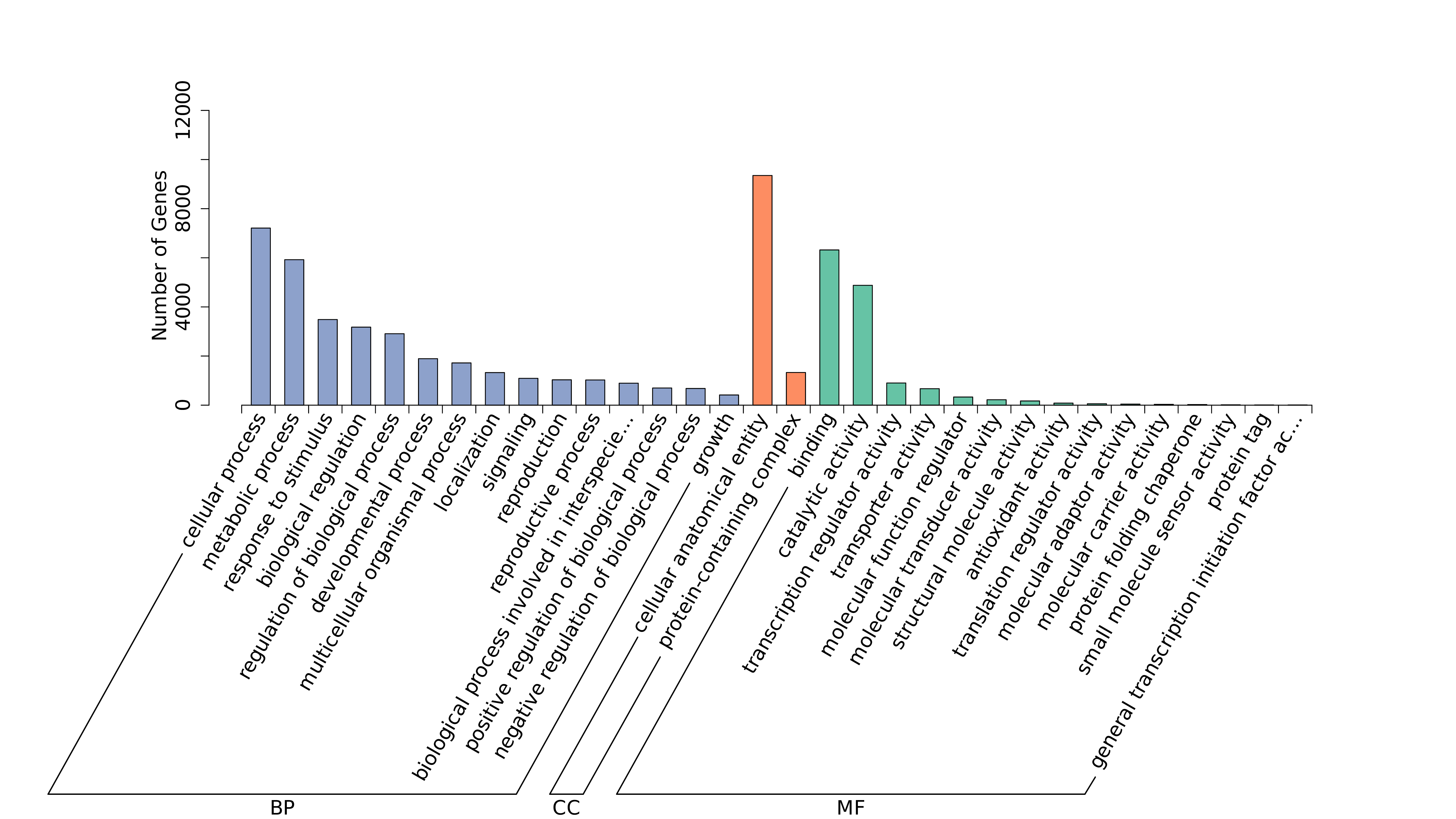

Supplement: Supplementary file 1 [file DataSheet1.zip › Supplementary Figure and Table/Supplementary Figure S9/Guiqi/GC-T_vs_GW2-T.GO.classification.png]

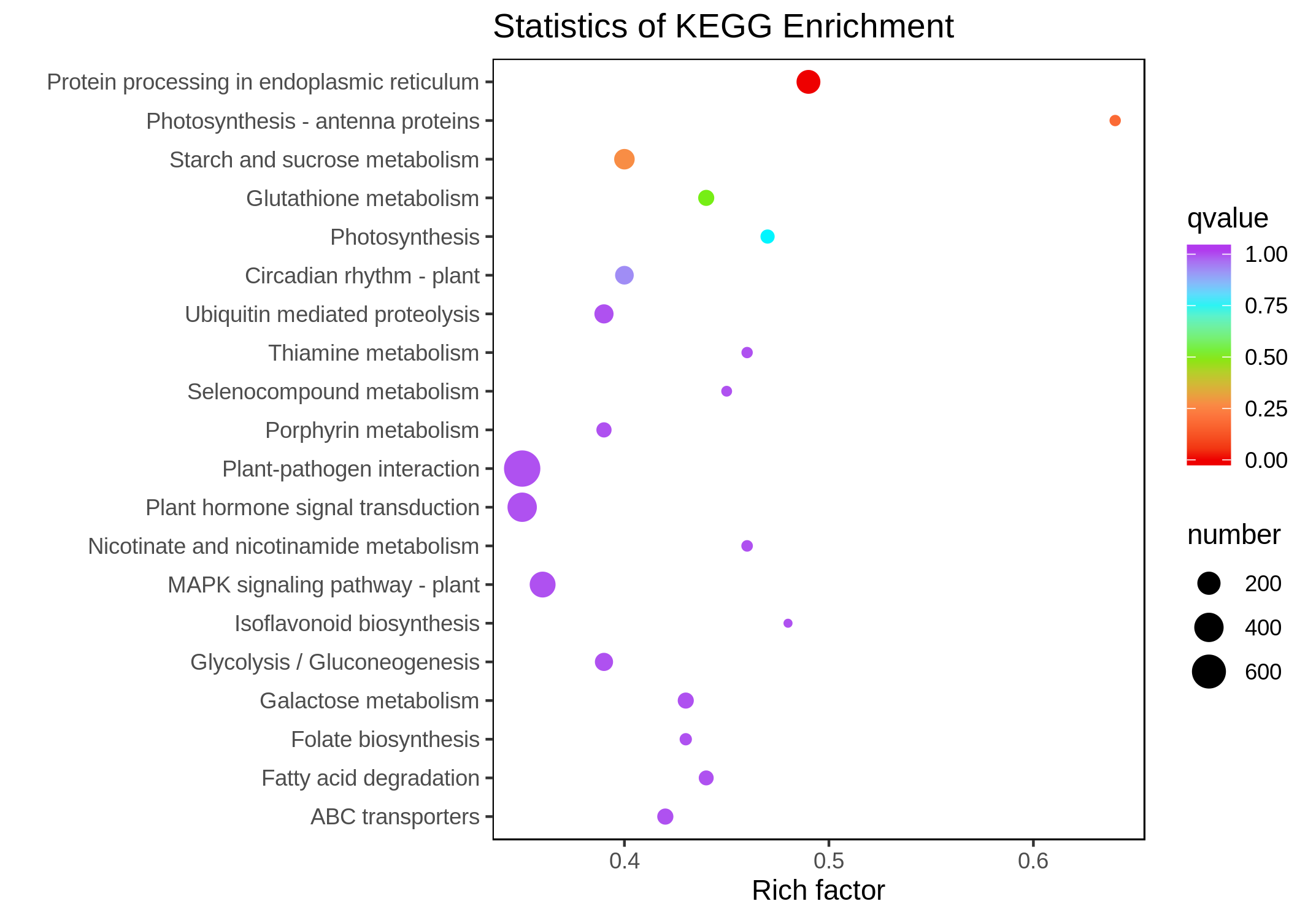

Supplement: Supplementary file 1 [file DataSheet1.zip › Supplementary Figure and Table/Supplementary Figure S10/Tainong/TC-T_vs_TW2-T.pathwayenrichment.png]

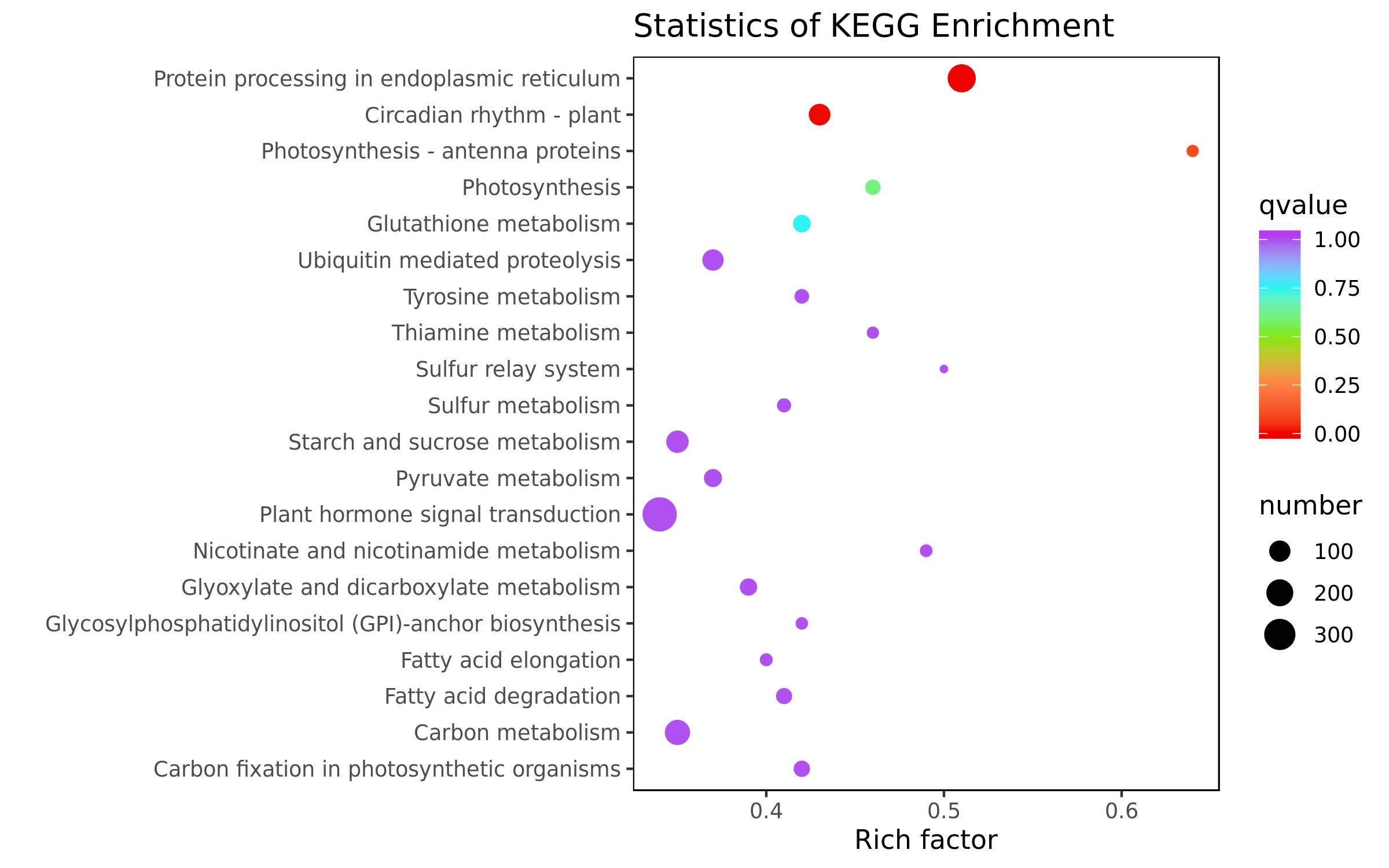

Supplement: Supplementary file 1 [file DataSheet1.zip › Supplementary Figure and Table/Supplementary Figure S10/M.indica/JC-T_vs_JW2-T.pathwayenrichment.png]

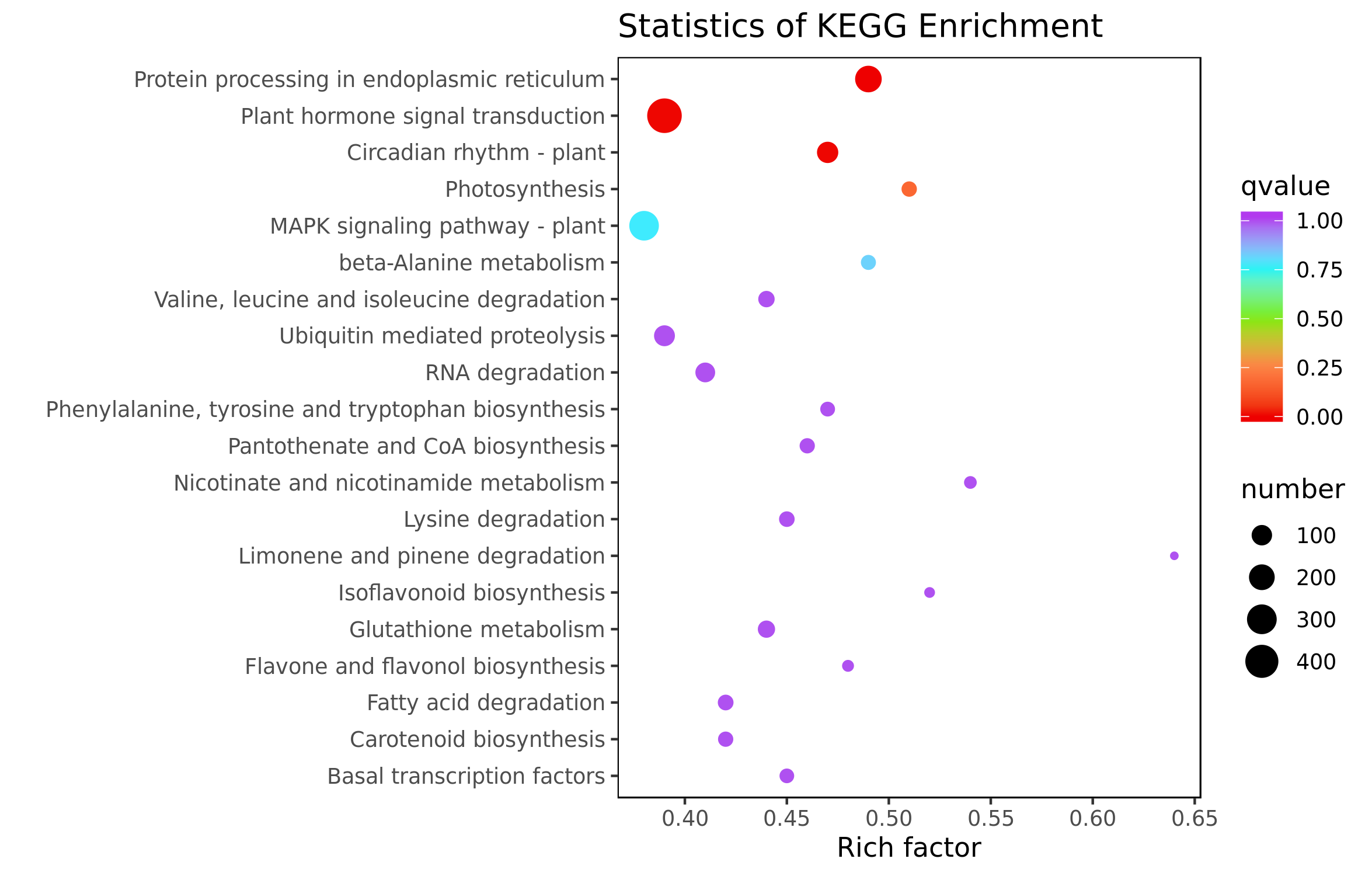

Supplement: Supplementary file 1 [file DataSheet1.zip › Supplementary Figure and Table/Supplementary Figure S10/Guiqi/GC-T_vs_GW2-T.pathwayenrichment.png]

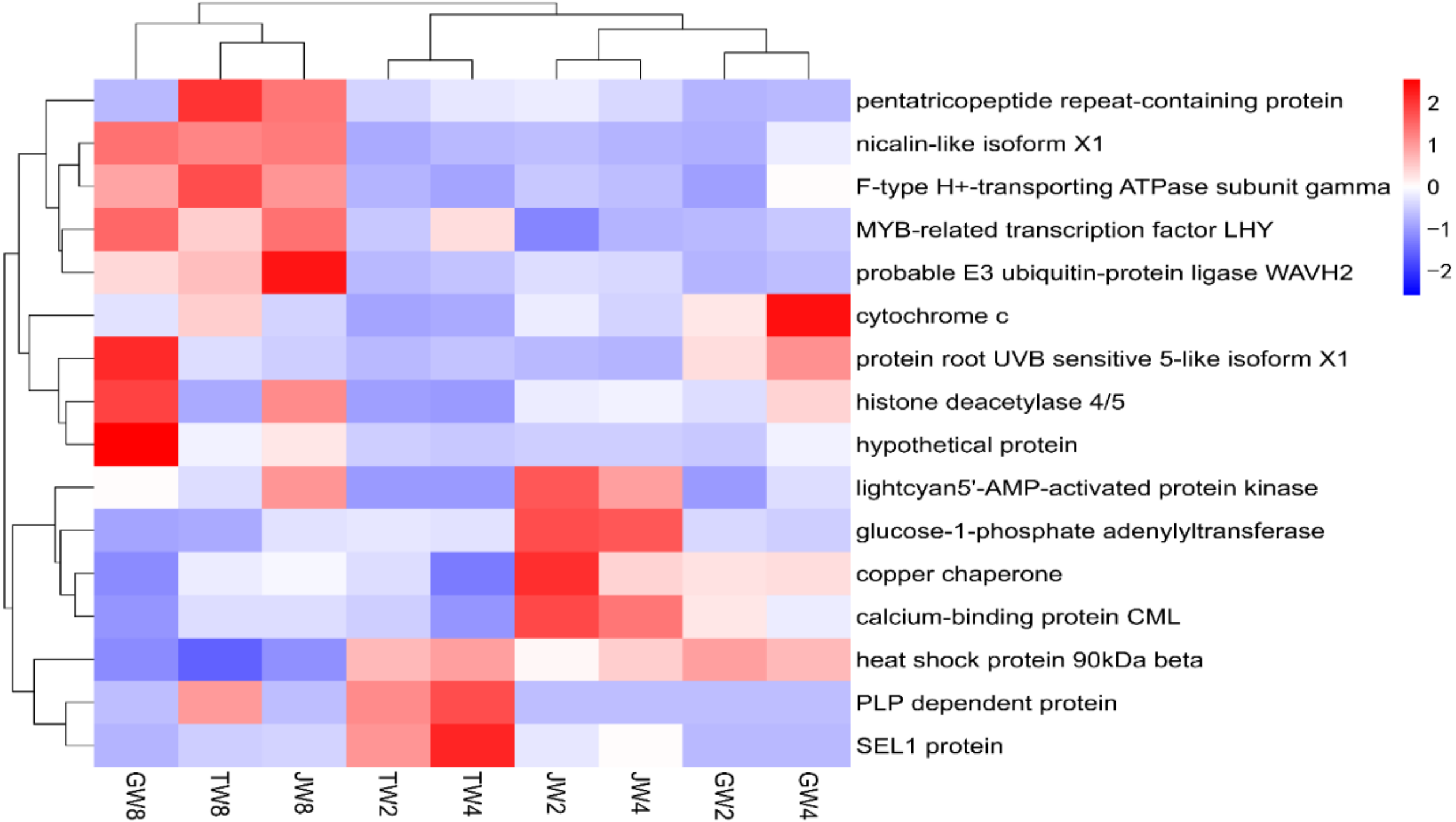

Supplement: Supplementary file 1 [file DataSheet1.zip › Supplementary Figure and Table/Supplementary Figures S14.pdf]
